# Chiral-Modified Nucleoside Analogues: From Bioactivity to Therapeutic Applications

**DOI:** 10.3390/ph19071131

**Published:** 2026-07-22

**Authors:** Anna A. Kozlova, Valentina N. Borokh, Vladimir E. Oslovsky, Cyril S. Alexeev, Mikhail S. Drenichev

**Affiliations:** Engelhardt Institute of Molecular Biology, Russian Academy of Sciences, Vavilova Street 32, Moscow 119991, Russia; ann.kozlova.a@gmail.com (A.A.K.); borokh-96@mail.ru (V.N.B.); vladimiroslovsky@gmail.com (V.E.O.); micelle@mail.ru (C.S.A.)

**Keywords:** chirality, nucleoside, biological activity, selectivity, toxicity, synergism, receptors, enzymes

## Abstract

Nucleosides are extensively employed for the development of pharmaceuticals, chemotherapeutic agents, and bioregulators. **Background/Objectives**: The introduction of an additional chiral functionality into a carbohydrate or heterocyclic base fragment may increase the selectivity of interactions with nucleos(t)ide-metabolizing enzymes and receptors and, in some cases, lead to more specific physiological activities. **Methods**: An improvement of the selectivity of nucleoside-based drugs can be achieved either by the chemical modification of a carbohydrate or a base constituent or by a combination of these two approaches. Additionally, stereospecific enzymatic cleavage of nucleos(t)ide prodrugs containing biodegradable substituents can reduce cytotoxicity and enhance bioavailability. **Results**: A series of enantiomerically pure nucleosides modified at the ribose or heterocyclic base were obtained by chemical and enzymatic methods. Novel antiviral or anticancer active compounds, inhibiting viral or cellular enzymes or activating cellular nucleoside kinases have been found among chemically synthesized derivatives. Some exhibit strengthened “ligand–receptor” interaction, acting on receptors of the purinergic signaling system. During recent extensive structure–activity studies, several drugs and their prototypes have been proposed for the treatment of viral infections: the 2′C-fluoromethyl derivative of sofosbuvir (anti-SARS-CoV, preclinical), VV-261 (SFTSV, phase I clinical), balapiravir (dengue, phase I clinical), mericitabine (approved drug for HCV), and lumicitabine (approved drug for RSV and HMPV). **Conclusions**: Modern literature data within the scope of the present review suggest that direct modification of nucleosides with various chiral functionalities can be considered as an approach to increase their efficacy, specificity and selectivity.

## 1. Introduction

**Figure 1:**
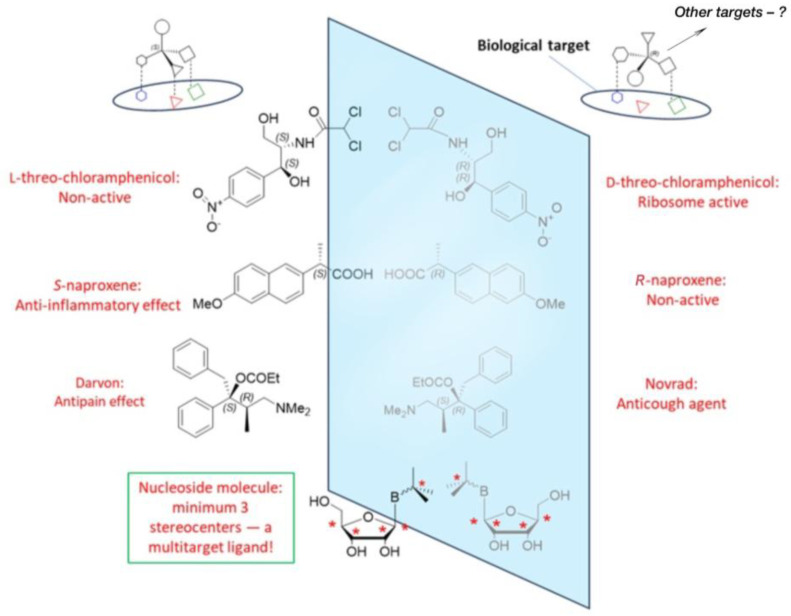
Examples of stereochemical modification and biological activity. Functional groups are highlighted by colored areas, and all chiral centers are marked with asterisks (*).

The majority of biologically active compounds interact with biological targets by non-covalent bonding. Most often, receptors with a characteristic spatial structure for the binding site participate as specific biological targets for interaction with low-molecular-weight ligands. Hence, there is a close relationship between a ligand’s structure and its pharmacological activity, reflecting a stereospecificity at target receptors. This relationship is due to the spatial availability of functional groups in a biologically active molecule with complementary sites on a target molecule. One enantiomer of a drug is likely to interact with a target molecule much better than the other, reflecting the “hand-and-glove” analogy. For example, the chloramphenicol D-threoisomer is used to treat of infections caused by Gram-positive and -negative bacteria by inhibiting peptidyltransferase activity in the 70S ribosome, while L-threo and D/L-erytroisomers do not bind with the 70S ribosome and possess no biological activity (Figure 1) [1]. In the case of naproxen, only the *S*-enantiomer decreases inflammation [2]. Often different enantiomers manifest different therapeutic properties. For example, the drug “Darvon” is a painkiller, while its enantiomer, known as “Novrad” appears to possess anti-cough efficacy [2]. Multiple examples of this have been shown, such as changes in taste, smell and other physiological properties that depend on the optical configuration of biologically active molecules [3,4,5]. Chirality influences a compound’s bioavailability and metabolism in cells and tissues. Two enantiomers may differ in binding affinity; therefore, therapeutic application of an optically pure compound can increase its selectivity and reduce the undesirable side effects caused by impurities of other optical isomers.

The rejection of racemic mixtures can also promote the rational usage of pharmaceuticals, which is important for overcoming drug resistance [6]. Since the end of the 20th century, there has been an increase in the number of synthetic drugs in the form of single enantiomers. Despite the growing interest in and increasing share of optically pure drugs, mixtures of stereoisomers are found in many therapeutic groups [1,7]. The opportunity to use drugs based on optically pure compounds, in contrast to drugs whose active substances are a mixture of enantiomers, was approved by the Food and Drug Administration (FDA) in 1992 [8]. Their development was driven by the fact that the two enantiomers of a given compound often exhibit different biological activities, evidenced by pharmacological, toxicological and pharmacokinetic studies proving that various stereoisomers undergo different metabolic pathways. One of the best examples of differences in properties between two enantiomers of a given compound is the thalidomide tragedy [9]. In 1999, the first example of the so-called “chiral switch” was observed, which is defined as the introduction of an optically pure drug that was previously produced as an equimolar mixture of enantiomers. As reported in 2006, 56% of drugs on the pharmaceutical market had chirality, and 88% of them were launched in the form of racemates [10]. By introducing chiral moieties, more effective binding to biological targets can be achieved, leading to improved pharmacological action. One problem linked to the application of enantiomerically (optically) pure biologically active compounds is that regioselective synthetic procedures must be developed and optimized when introducing chiral substituents into a core molecule. In this regard, chemical and enzymatic modification of natural compounds such as nucleosides is considered as a fruitful approach to drug design. Natural nucleosides contain between four and six stereoisomeric centers (Figure 1); various modifications at a heterocyclic moiety due to metabolic conversions as in the case of minor tRNA components, result in a number of centers at the upper end of this range. These reaction centers generate great structural diversity and can strengthen the binding affinity of nucleoside analogues for protein receptors.

The relevance of this review is linked to the following key aspects: it covers biological activity towards various targets and structure–activity relationships of nucleos(t)ide analogues containing an additional chiral substituent. The present review consists of five main sections, the first briefly details the general mechanisms of action of stereospecific nucleoside analogues, and the second, third and fourth concern structure–activity relationships and describe the problem of the increase in selectivity increase in different forms of chemically modified nucleosides and their analogues. Some novel stereoselective chemo-enzymatic approaches are also discussed there. The final section covers increases in bioavailability and describes several recently discovered prodrug forms.

## 2. Targeting General Metabolic Pathways in Nucleos(t)ide Derivatives

### 2.1. Nucleic Acid Biosynthesis and Nucleoside-Converting Enzymes

**Scheme 1:**
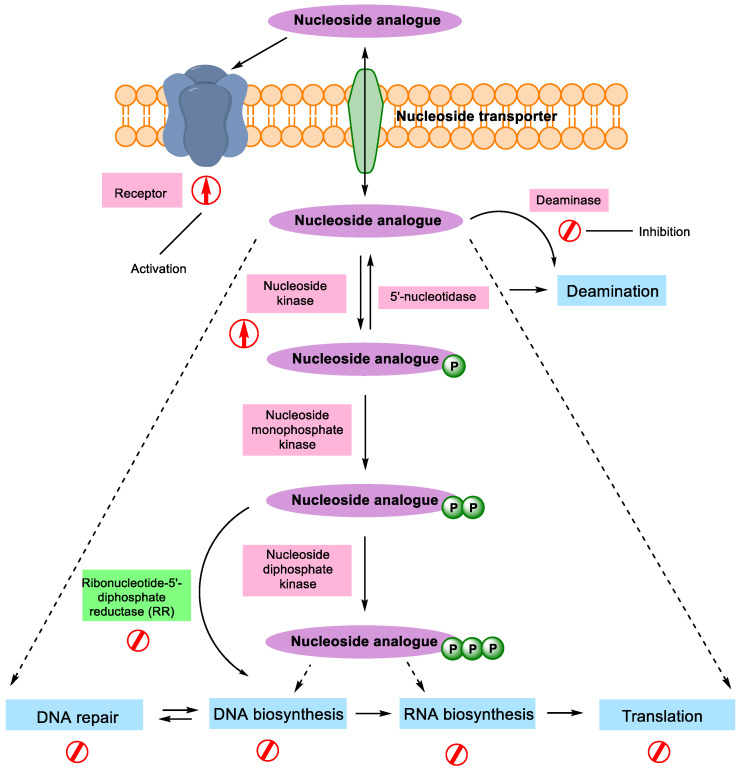
Activation/inhibition of metabolic pathways by nucleoside analogues bearing chiral groups.

The general mechanism of nucleoside action involves their 5′-triphosphorylated (nucleotide) forms. Nucleosides enter the cell through nucleoside transporters. This ensures their movement into the cytosol [11], where they gradually undergo phosphorylation steps, as depicted in Scheme 1 [12]. The first phosphorylation step is rate-limiting and controlled by two enzymes—nucleoside kinase (nucleoside phosphorylation) and 5′-nucleotidase (monophosphate dephosphorylation reaction). The second step proceeds under the action of nucleoside monophosphate kinase, and may be rate-limiting in several nucleosides, such as pyrimidine nucleosides [13]. The third phosphorylation step takes place under the action of nucleoside diphosphate kinase. Phosphorylated nucleosides can further interact with viral or host enzymes either by incorporation into DNA/RNA by changing their properties, or through direct inhibition of nucleic acid (NA)-converting enzymes.

**Figure 2:**
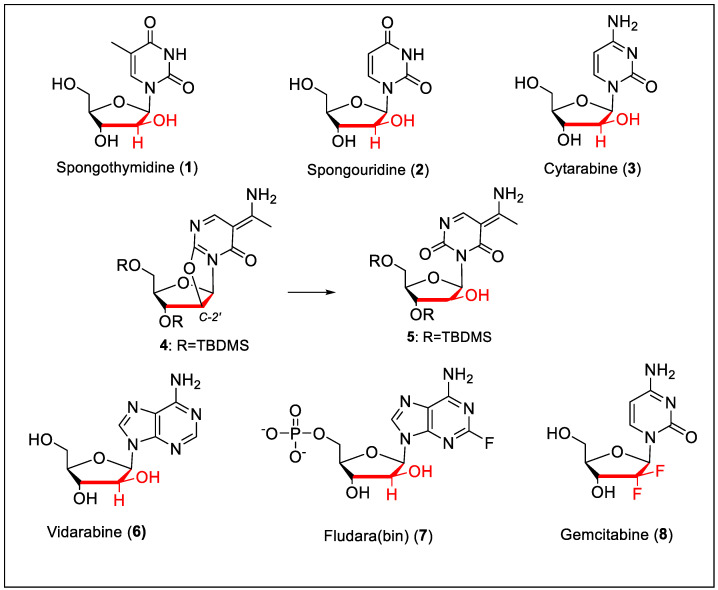
Naturally occurring arabinonucleosides and their fluorinated derivatives. Key structural fragments are highlighted in red.

Slight, “one-point”, selective modification of nucleoside molecules *via* reversion of a bond’s configuration at one carbon center in a glycoside moiety can be used for drug design from natural compounds. Detailed “one-point” modifications of a carbohydrate moiety in a nucleoside structure were described in an excellent review article [14]. This type of modification changes the nucleoside properties and conserves their ability to bind to native nucleos(t)ide-converting enzymes. Examples of the modification of pentose moieties in nucleosides include the most well-known transformation route, resulting in arabinonucleosides. The nucleosides **1**,**2** were isolated for the first time from marine sponges *Tethya crypta* in the middle of the 20th century [15] and have since become the base structures for the synthesis of the potent antitumor drugs cytarabine **3**, the anti-herpes drug vidarabine **6,** the potent cytostatic drug fludarabin **7**, and a fluorinated and phosphorylated analogue of the latter and antitumor drug gemcitabine **8** (Figure 2). The drugs **6**–**8** displayed cytostatic activity and affected the DNA-synthesizing capacity of cells [16,17]. The molecular basis of arabinonucleosides’ action is strongly associated with conformational changes inside the cycle, caused the *O*(2′)-hydroxyl group in arabinose, which is reveresed compared to ribose. This exerts significant influence on the conformational properties of ribose rings with a high amounts of *C*(3′)-endo conformers. In adenine-based nucleosides, the energy barrier between *anti–syn* base interconversions of the base is considerably higher in arabinoadenosine (*ara*-A) than in adenosine, even for C(2′)-endo sugar pucker. The higher conformational stability of *syn*/*anti* forms may facilitate an energetically favorable interaction with some cellular enzymes [18,19].

Various approaches have been tested to improve the pharmacodynamic properties of arabinonucleosides. Chemical modification of the pyrimidine moiety with additional lipophilic groups led to biological activity against both leukemia cells and human T-lymphocytes at concentrations of 18 and 30 µM, respectively (see compounds **4**–**5** in Figure 2). Nevertheless, compounds of this class are significantly inferior to the lead compound cytarabine. Additional cycle formation between the 2′-hydroxyl group and keto-functionalities of the pyrimidine base, which is characteristic of arabinonucleosides, probably increases biochemical reactivity and thereby leads to higher toxic effects in cells (compound **4**, Figure 2) [20].

It has been reported that there is less cleavage of the *N*-glycosidic bond in nucleosides with a fluorine in the 2′*C*-position as well as less deamination under the action of enzymes that play a significant role in the deactivation of modified analogues [16,21]. Placing a fluorine atom, as well as oxygen-containing substituents such as methoxy and methoxyethyl, at the C2′ position promotes pentafuranose puckering toward the *C*3′-endo state, which can stabilize NAs’ duplex formation, inhibiting NA biosynthesis [22].

**Figure 3:**
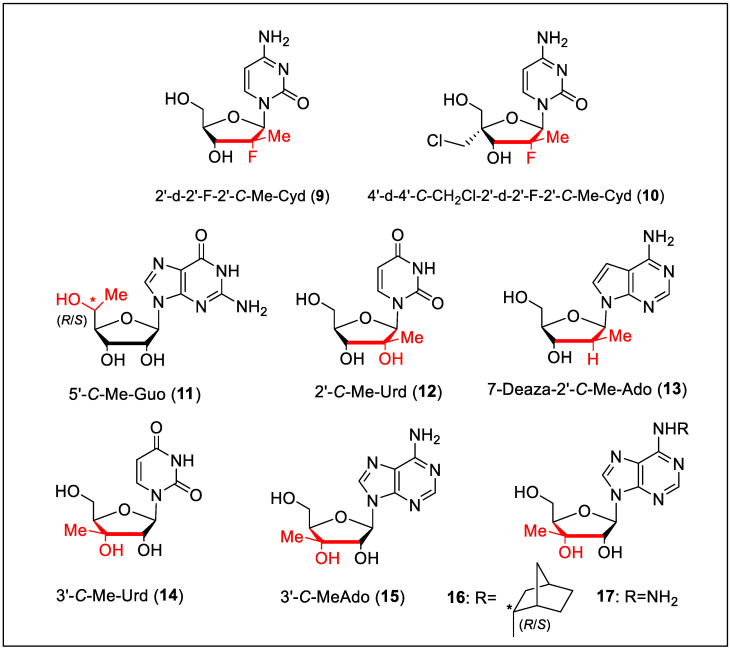
*C*-Methyl derivatives of arabinonucleosides and related compounds.

“One-point” modification *via* the introduction of a *C*-methyl group into pentafuranose leads to diverse biological activity due to conformational changes in the resulting nucleosides (Figure 3). The introduction of *R*- or *S*- 5′-*C*-methylguanosine (**11**) into RNA strands decreases the thermal stability of RNA duplexes and increases RNA stability during exonuclease-catalyzed hydrolysis [23].

Potent inhibition of hepatitis C virus (HCV) polymerase NS5B is induced by 2′-*C*-methyl nucleoside analogues (Figure 3), while 7-Deaza-2′-*C*-methyladenosine (**13**) and 2′-deoxy-2′-fluoro-2′-*C*-methylcytidine (**9**–**10**) suppress the replication of RNA and DNA viruses [24,25]. It has been shown, that on a molecular level, 2′-*C*-methyl derivatives **9**–**10** inhibit polymerase synthesis of viral NAs in HCV and respiratory syncytial virus (RSV) [26].

Examples of the wide-ranging biological activities of *C*-methylnucleosides (Figure 3), including anticancer and antiparasitic activities, have been published elsewhere [27,28,29,30,31]. Notably, the precise mechanism of 2′-*C*-alkylcytosine action is associated mostly with competitive inhibition of cellular DNA-polymerases [30], while antitumor activity of 2′-*C*-methyladenosine is associated mostly with inhibition of ribonucleotidediphosphate reductase enzyme (RR) and is difficult to elucidate, due to its limited metabolic stability [28]. RR-inhibition leads to cellular toxicity through biochemical formation of 5′-diphosphate forms, as depicted in Scheme 1. Further experimental efforts have revealed that 3′-*C*-methyladenosine is an alternate antitumor compound that acts on RRs. A series of *N*^6^- and *C*2-substituted derivatives of 3′-*C*-methyl adenosine **17** have shown modest antitumor activity on various cell lines, with *N*^6^-norbornyl derivative **16** identified as a hit compound [29]. Replacing the bulky norbornyl substituent with a sterically compact and reactive amino group in **17** led to a significant increase in antitumor activity [31], possibly due to better intracellular phosphorylation and improved ability to interact with RR.

**Figure 4:**
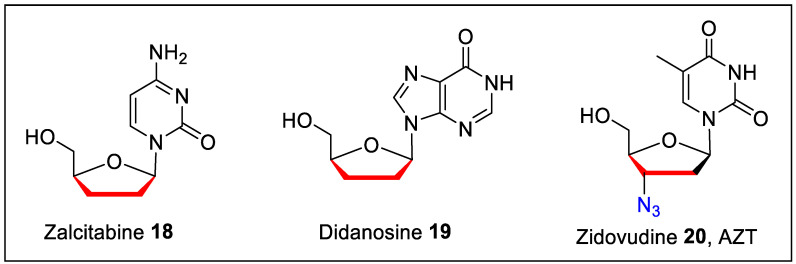
Structural features of zalcitabine, didanosine, and zidovudine.

Combining modifications at the 2′*C* and the 3′*C* positions appears to be a particularly effective method for developing antiviral drugs (Figure 4). The first successful drugs obtained according to this method were 2′,3′-dideoxycytidine (ddC, zalcitabine **18**), 2′,3′-dideoxyinosine (ddI, didanosine **19**) and 2′,3′-dideoxy-3′-azidothymidine (AZT, zidovudine **20**). These drugs exhibit a chain termination mechanism and were successfully used against HIV in the second half of the 20th century, which at that time had become widespread in human populations [32].

### 2.2. Receptors and Signaling

**Figure 5:**
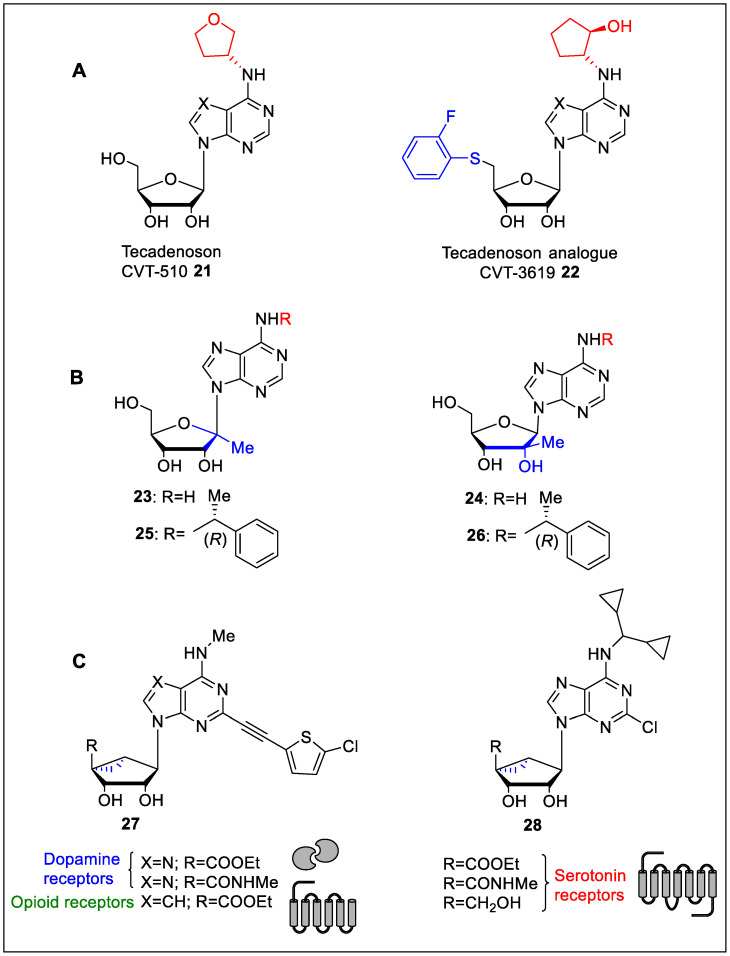
Modified nucleosides as receptors’ ligands. (**A**) AR-binding drugs; (**B**) Structure-activity relationship in AR-binding activity; (**C**) Examples of modified nucleoside structures interacting with other types of receptors.

The discovery of the previously unknown ability of adenosine and adenosine triphosphate (ATP) to bind with specific receptors, later defined as components of the purinergic signaling system, opened up an opportunity for the use of nucleosides and their analogues as bioregulators [33]. A purinergic signaling system consists of adenosine receptors (ARs) and also some purine-activated P_2_X-receptors; the latter alter the ion conductivity of cellular membranes. In mammals there are four types of ARs—A_1_, A_2a_, A_2b_, and A_3_—and several subtypes of P2Y and P2X [34]. Activation of ARs and P2Y through binding with a ligand leads to conformational changes in the associated G-protein, which inhibits or stimulates adenylate cyclase’s ability to synthesize cyclic adenosine monophosphate (cAMP), thus further triggering intracellular signal cascades, as evidenced by experimental data. At a glance, A_1_ and A_3_ ARs are mostly linked to G**i** (***i***nhibiting) protein subunits. Therefore, the action of A_1_ and A_3_ agonists mostly inhibits adenylate cyclase activity and, hence, decreases the activity of protein kinases. On the contrary, A_2ab_ ARs act on G**_s_** (***s***timulating) proteins. A_1_AR and A_2b_R stimulation can also activate inositol triphosphate depletion pathways [35,36]. ARs exhibit different distributions in various tissues; therefore, a biologically active molecule with high specificity is needed to achieve a desirable physiological effect [35,37,38,39,40,41,42]. In this regard, some modified purine nucleosides and their analogues have been shown to be extremely selective, achieving a desired biological effect at concentrations in the nanomolar range [42]. Compounds acting on the A_1_ and A_2_ receptor groups can be considered as potential drugs, regulating neuronal and muscular tone (striated and smooth), and therefore could be used in the treatment of arrhythmia, ischemia, and glaucoma [43,44,45,46,47]. Separately taken, the A_1_ and A_2a_ receptors can act as targets for the treatment of asthma [47]. The A_3_AR can be considered as a biomarker for cancer diagnostics [34] due to its high expression level in cancer cells. It has been shown that A_3_AR agonists exert antiproliferative action on cancer cells in vitro and in vivo, blocking the cell cycles in the G_0_/G_1_-phase and inducing apoptosis [48,49]. Targeting A_2B_AR, and thus triggering cancer cell proliferation and solid tumor growth and accelerating angiogenesis and metastasis, has important potential for the treatment of various cancers [41,42]. Adenosine, a natural ligand, exhibits low affinity for ARs [50,51] and a short plasma half-life [50]. However, adenosine modification with a lipophilic moiety may lead to prolonged physiological activity, as demonstrated in the drug tecadenoson (**21**), which bears a chiral substituent at the adenine 6 position (Figure 5A) [50]. Its selective action on A_1_ receptors efficiently relieves supraventricular tachycardia without notable side effects [52,53]. Partial agonism towards A_1_AR was found in CVT-3619 (GS-9667) (**22**), an analogue of tecadenoson (Figure 5A) [54,55]. A number of studies have revealed that *N*^6^-substituted adenosine ligands, structurally related to the tecadenoson ligand and containing a hydrocarbon fragment with one or more chiral centers trigger mostly agonistic effects on ARs; this has been described in more detail in our previous review (Figure 5B) [56]. In contrast, adenosine receptors poorly tolerated modification in the carbohydrate fragment of nucleoside ligands because ribose hydroxyls participate in hydrogen bond formation with His and Ser residues in the receptor helix [37,57]. Most of ARs’ binding capacities are reduced with *C*-methyladenosine ligands, which indicates that the antitumor activity of the latter is not associated with ARs [29,58]. It was found that the 1′*C*, 2′*C,* 3′*C* and 4′*C* -methyl modification in adenosine resulted in a decrease in affinity (Figure 5B). However, the combination of the 1′*C* or 2′*C*-methyl group in the pentafuranose moiety with substitution at the purine *N*^6^-position partially restored their affinity to A_1_ and A_2A_ARs (Figure 5B, compounds **25** and **26**). This may be due to the tendency of the 1′(2′)-*C*-methylcarbohydrate moiety for *N* (“North”)-puckering, which was shown to favor binding with ARs. A_3_ARs tolerated only a combination of the *N*^6^-(*R*-phenylethyl) substituent and the 1′-*C*-methyl group [37].

Some adenosine analogues possess cytostatic and anticancer activity acting on a set of regulatory cellular proteins [59]. The corresponding structurally optimized nucleoside derivatives may interact with target proteins that normally do not bind to nucleosides. This can be achieved by replacing of the ribose in the adenosine molecule with structurally rigid fragments that lean towards *N*-conformation and bearing polar functional groups. Antagonists of opiate receptors and modulators of serotonin and dopamine receptors, as well as novel anticancer compounds, have been discovered based on this replacement (Figure 5C) [60,61,62].

### 2.3. Other Targets: Combined Therapy

Nucleoside-based drugs can have toxic effects due to the inhibition of enzymes that metabolize natural nucleosides. This is usually the result of prolonged exposure to these drugs during the treatment of latent and chronic infections. Resistance occurs when mutations accumulate in a pathogenic genome, leading to the formation of drug-resistant strains [63,64]. The combined use of several drugs targeting different replication pathways may significantly enhance the efficacy of antiviral therapy through a synergistic effect while reducing on patient toxicity by lowering the drug dosage.

Cancer cells are characterized by a disbalance of key metabolic pathways, catalyzed by CTP-synthetase, thymidylate synthase, dihyrofolate reductase, IMP-dehydrogenase, ribonucleotide reductase, DNA polymerase and DNA methyl transferase, SAH-hydrolase, which are significantly activated in some transformed cells [63,64,65,66]. In some cases, cytotoxicity is highly possibly associated with the inhibition of SAH-hydrolase by some synthetic L-nucleosides [67,68,69,70]. The use of antimetabolic drugs, which inhibit a range of hyperactive enzymes that cause intensive cell differentiation, may sufficiently increase the efficacy of anticancer therapy. Additionally, nucleotide antimetabolites may be considered as inducers of immune response, as was shown for several L-derivatives [71]. Experimental data show that nucleotide antimetabolites, in combination with differentiation inducers, can significantly increase the effectiveness of anticancer therapy [14], for example, this includes puromycin and analogues inhibiting protein biosynthesis [60]. The recruitment of the diasteromeric drug Fludara **7** in combination with cyclophosphamide for the treatment of chronic lymphocytic leukemia resulted in a significantly higher complete remission rate and overall response rate compared with the use of fludarabine alone [72]. The use of a combination of nucleoside and non-nucleoside RT-inhibitors in HIV-treatment afforded reduced dosing frequency for the first FDA-approved triple combination of efavirenz with tenofovir and emtricitabine [73]. Modern clinical trials aim to improve the safety and tolerability of the combination of efavirenz with tenofovir and lamivudine in treatment-naïve patients with HIV [74].

In cancer cells, DNA-repair enzymes prevent the action of DNA-alkylating and DNA-injuring drugs. Therefore, the therapeutic outcome is determined by the combination of chemotherapeutics and DNA repair inhibitors; this may be a novel basis for efficient anticancer treatment. A promising example is tyrosyl-DNA phosphodiesterase-1 (Tdp1), which plays a key role in the removal of Top1-DNA adducts stabilized by Top1 inhibitors, such as topotecan and its clinical analogues. The general mechanism of topotecan’s action is the stabilization of covalent complexes of Top1 tyrosine and the 3′-DNA end [75]. Cell lines with a high level of Tdp-1 expression have low sensitivity to Top-1 inhibitors including topotecan [75,76,77,78,79,80,81]. Tdp1 inhibition can therefore significantly strengthen the action of clinically used Top1 inhibitors and thus increase the efficiency of antitumor therapy. Some lipophilic nucleoside derivatives bearing additional chiral centers appeared to be good Tdp1 inhibitors, exhibiting various mechanisms of action depending on the configuration of their chemical bonds [82].

## 3. Structure–Activity Relationship 1: Widening a Carbohydrate Functionality

### 3.1. L-Nucleosides

In the early research by Schinazi and colleagues (1992–1993) [83,84,85], it was shown that in several cases, L-nucleosides are even more rapidly phosphorylated compared to D-analogues and produced a more pronounced inhibitory effect on human immunodeficiency virus reverse transcriptase (HIV RT) and/or herpes B virus (HBV) DNA polymerase [6]. The discovery of nucleosides L-oxathiolane and L-dioxolane triggered the syntheses and biological activity studies of a series of their analogues. Several excellent reviews are devoted to the structural features of stereochemically modified nucleoside-based drugs and prototypes [86,87,88,89], but a comparison of their structural and biological activity may be beneficial for further consideration and drug design. Therefore, we have summarized their features in Table 1 for clarity.

L-(−)-derivative **29** is more active and less toxic than its D-(+)-form **30** ((+)-BCH-189), as was shown in the studies by Schinazi (1992) [83,84,90]. Lamivudine (3TC, **29**) has also been shown to be more active against hepatitis B virus (HBV) [90], compared to the D-(+)-enantiomer **30**. The different activities and toxicities of these two enantiomers are explained by their metabolic and pharmacodynamic characteristics. Both enantiomers are substrates of the deoxycytidine kinase, which converts them into monophosphates. Subsequent phosphorylation is carried out by nucleotide kinases. Lamivudine (3TC) has a greater affinity for deoxycytidine kinase, which results in more effective binding with the target at the first step of phosphorylation. This is why lamivudine (3TC) has greater activity compared to its D-form. It has been reported that both triphosphates acted as analogues of deoxycytidine triphosphate (dCTP), competitively inhibiting HIV RT or HBV DNA polymerase, and both could be incorporated into viral DNA, causing chain termination at the replication step [86,89]. Additionally, L-enantiomers appeared to be more stable to metabolic deamination of the cytosine moiety compared to their D-analogues [106]. The docking studies revealed that the triphosphate form of 3TC is appropriate for interactions with HBV RT: the heterocyclic base was placed near Met204, while the oxathiolane ring occupied the small hydrophobic pocket in the dNTP binding site. The positively charged amino acid backbones Lys32 and Arg41 are involved in ionic interactions with triphosphate groups. The relevance of this docking model is proven through the replacement of key amino acids, located close to lamivudine, with those, leading to mutability and drug resistance and thereby disrupting ligand–receptor interactions [107].

The introduction of fluorine atoms into nucleoside carbohydrate residues modulates their biochemical properties [21]. Emtricitabine **31** (Emtriva^®^, FTC) is a 5-fluorinated lamivudine (3TC) analogue [85,108,109]; this type of L-nucleoside drug is comparable to 3TC in terms of its antiviral activity, resistance and cellular metabolism. The L-enantiomer (FTC) proved to be 100 times more effective than its D-analogue **32** (Table 1) [83] and possessed the same effectiveness against HBV as lamivudine (Table 1). The 5-triphosphate form of FTC (**31**) showed 10-fold tighter binding than triphosphates of 3TC during reverse transcription catalyzed by HIV-1 RT, which prevented high mutation frequency both in the viral genome and infected cells [83] compared to lamivudine, as demonstrated by in vitro experiments on PBM cells [85].

L-Troxacytabine (**33**, Troxatyl^®^, L-OddC) and its 5-fluorinated analogue (not shown) possessed high cell toxicity despite their pronounced antiviral effect. For the former, this is due to inhibition of DNA polymerases by L-OddC-5′-triphosphates formed during intercellular phosphorylation. L-OddC was the first L-nucleoside analogue to display antitumor activity. In comparison with the D-form (**34**), troxacitabine is not degraded by deoxycytidine deaminase, which can prolong its effect as a drug. L-OddC suppresses the reproduction of DNA viruses and possesses a pronounced cytotoxic effect on cancerous cell lines [92]. The antiviral activity of L-OddC is sensitive to its chemical modification, as the structurally similar 5-azacytidine forms of L-OddC possess less pronounced antiviral effects, but are also less toxic to cells [93].

1-(β-*D*-2′-Fluoro-2′-deoxyarabinofuranosyl)-5-methyluracil (D-FMAU, **51**) effectively inhibits HBV replication but unfortunately displayed neurological toxicity [109], while L-FMAU (**50**, Levovir^®^, Revovir^®^) appeared to be both an active inhibitor of HBV and a non-toxic compound for host cells [100] (Table 1). The difference in the actions of D-FMAU and L-FMAU may be explained by their differing metabolic conversions in cells. Both nucleosides convert to the corresponding 5′-triphosphate forms (D-FMAUTP and L-FMAUTP). The first phosphorylation step proceeds upon binding of D-FMAU and L-FMAU to different cellular proteins: D-FMAU is phosphorylated by deoxypyrimidine kinase while L-FMAU phosphorylation is catalyzed by thymidine kinase or deoxycytidine kinase. Subsequent phosphorylation may be facilitated by different cellular kinases. Both D-FMAUTP and L-FMAUTP inhibit HBV DNA polymerase, but D-FMAU also inhibits DNA polymerase Y, causing mitochondrial toxicity. Selectivity towards the viral enzyme is a key property of L-FMAU, which makes it a candidate for the treatment of HBV-caused infections [86]. Additionally, L-FMAU can suppress reproduction of the Epstein–Barr virus (EBV) [110]. L-FMAUTP is not a substrate of EBV DNA polymerase, but may inhibit DNA elongation and its 3′-5′-exonuclease activity in a non-competitive manner [110]. It is interesting to note that L-FMAU did not manifest significant activity against herpes virus (HSV) in contrast to D-FMAU [111]. L-FMAU also does not suppress HIV-1 replication [100], even if anti-HIV activity is characteristic of nucleoside inhibitors of HBV [103,111]. Testing of a series of compounds revealed that 2′-fluorinated 2′,3′-didehydro-2′,3′-dideoxynucleosides exhibited high anti-HIV and anti-HBV antiviral activity on various cell lines [103], with L-isomers being more active and less toxic than the corresponding D-isomers. 4′-Thionucleosides as FMAU analogues (see Table 4), in which an oxygen atom was substituted with a sulfur one, also retained their antiviral activity and low cytotoxicity [21].

Unsaturated 2′-fluorinated nucleosides have shown strong anti-HIV and antiviral activity against HBV, with the L-enantiomers **52**, **54** being more effective and less toxic than their natural D-analogues **53**, **55** (see Table 1).

L-d4C (**41**) and its fluorinated derivative L-Fd4C (**52**) and its 2’-fluoro analogue L-Fd4FC (**54**) are potent compounds against HIV and have very strong anti-HBV activity, but display significantly lower anti-HIV activity compared to elvucitabine (L-d4FC, **49**) [97,102,103]. They also have low toxicity of up to 100 µM in PBM, Vero and CEM cells. Nevertheless, 2′,3′-didehydro-2′,3′-dideoxy-2′-fluoro-D-cytidine **53** (D-Fd4C) exhibits comparable anti-HIV activity and lower anti-HBV activity compared to L-analogue **52** [102]. It also has lower toxicity in several cell lines. Structurally rigid L- and D-cytidine analogues **43**–**44** exhibit drastically low activities [98], while structurally rigid L- and D-thymidine analogues **45**–**46** had prominent anti-HIV activity, even higher than that of several commercial drugs. D-Thymidine analogue **46** displayed even higher anti-HIV and anti-HBV activity than L-isomer **45.** Both compounds displayed low cytotoxicity on the MT4 cellular test system. The purine analogues **58**-**59** were practically inactive compared to **45**-**46**. Various modifications with cyanide groups at position C-3′ of a carbohydrate-mimicking residue (compounds **47**, **48**, **60**) drastically decreased toxicity [99]. Replacement of the pyrimidine residue in **52** or **54** with a purine residue, as in **56**, did not significantly increase the antiviral effect or cytotoxicity [98]. Adenine-derived L-Fd4A (**56**) inhibited HIV-1 and HBV reproduction in a micromolar range of concentrations without notable cytotoxicity, but displayed 10 times less activity as an HIV inhibitor and a 3 order of magnitude lower activity as an HBV inhibitor compared to the most active compound, **54.** Neither L-Fd4C (**52**), nor L-Fd4A (**56**) exhibits any effect on the mitochondrial DNA of CEM cells under prolonged exposure.

Despite some contradictory data in Table 1, the general mechanism of action for L-nucleosides features intracellular phosphorylation as a basic molecular mechanism of their action. In a biochemical sense, a “non-natural” carbohydrate fragment structure is not compatible with the majority of cellular enzymes, e.g., RR, which is considered as a target for cytotoxic nucleoside analogues [28,29]. Therefore, L-nucleosides are more often recognized by less-specific viral enzymes. For example, D- and L-5-halogen and 5-halogenvinyl-containing nucleosides and their dioxolane analogues have also shown activity against several DNA viruses. Among them, 5-iodo-L-dioxolanouridine (L-I-OddU, **35**) should be mentioned as the most potent non-toxic and highly selective agent against several DNA viruses. In the case of EBV, its molecular mechanism of action may be associated with the selective 5′-phosphorylation of L-I-OddU to L-I-OddUMP with viral thymidine kinase. In this pathway, L-I-OddU acts as a selective alternative substrate that may affect toxicity only in infected cells [97]. In several cases, L- and D-nucleoside analogues can be phosphorylated by different cellular kinases, further participating in interactions with a set of different proteins. Several L-2′-deoxynucleosides of thymidine (**37**, Telbivudine, Tyzeka^®^, Sebivo^®^, L-dT), cytidine (**39**, L-dC) and adenosine (β-L-dA) possess anti-HBV activity without pronounced cytotoxic effects on normal mammalian cell lines [86,95]. During phosphorylation, L-dT and L-dC convert to their triphosphate forms in the presence of various kinases: deoxycytidine kinase, thymidine kinase-1 and thymidine kinase-2. Unlike Telbivudine (L-dT), whose only metabolite is its triphosphate analogue (L-dTTP), L-dC is metabolized to both L-dCTP and L-dUTP. L-Deoxynucleoside triphosphates (L-dNs-TP) inhibit HBV replication without noticeable effects on healthy cells (Table 1), and they also inhibited woodchuck hepatitis virus (WHV) DNA polymerase at concentrations in submicromolar and micromolar range, respectively [86,95]. Nevertheless, despite showing success as drug prototypes in cell experiments, telbivudine and FMAU appeared to show side effects, such as myopathy, peripheral neuropathy and neurotoxicity, during clinical trials [21,112]. This physiological response indicates the existence of unknown targets for L-nucleosides.

The combined use of several drugs targeting different replication pathways may significantly enhance the efficacy of antiviral therapy due to synergistic effects and may mitigate toxic effects due to a reduced dosage. The use of 3TC (**29**) has a disadvantage, as point mutations in HBV DNA polymerase and RT (M184V) HIV type 1 lead to drug resistance. However, this disadvantage may be overcome by using lamivudine in combination with AZT **20** and protease inhibitors, e.g., nelfinavir, indinavir or ritonavir. Treatment of cells with 3TC triggers the formation of TC-resistant HIV strains, bearing the M184V mutation in RT [89]. Nevertheless, the use of 3TC in combination with RT inhibitors such as AZT has been shown to reduce the formation of both TC- and AZT-resistant viral strains [86]. The discovery of L-oxathiolan and L-dioxolan nucleosides as biologically active compounds has led to studies of non-natural L-nucleoside analogues based on the known anti-HIV drugs in a combination with other medications. For example, conformationally rigid analogues of “d4”, 2′,3′-didehydro-2′,3′-dideoxy-derivatives should be mentioned here. These compounds inhibit the growth of healthy cells at concentrations below 20 µM. When elvucitabine was combined with various anti-HIV drugs, increased activity was observed. Both compounds, when separately taken, inhibit the growth of normal cells, with elvucitabine acting on mitochondrial DNA. Combinations of Elvucitabine with d4T or AZT resulted in synergism, and in the case of ddC/ddI, in an additive action [96]. As was reported in the above-mentioned study, the enzyme responsible for the initial phosphorylation of elvucitabine appeared to be cytoplasmic deoxycytidine kinase, which is in accordance with the L-nucleoside phosphorylation mechanism.

In a series consisting of both D- and L-nucleosides compounds, antiviral activity can be detected, with L-deoxyforms of natural nucleosides displaying high anti-HIV and anti-HBV activities and often low toxicities. This antiviral effect is sensitive to modifications of the glycosyl moiety, especially when replacing the deoxyribose ring with a flexible (thioxo)lane ring. In this case, L-nucleoside forms acquire a higher antiviral activity compared to D-forms and may even possess different mechanisms of biological action. On the contrary, structurally rigid D-configuration glycosyl analogues display more pronounced effects compared to those in the L-configuration. Some biological effects may be heightened upon addition of fluorine atoms to the glycosyl moiety and/or pyrimidine rings or upon using different combinations of compounds.

### 3.2. Nucleotide and Nucleotide-like Chiral Prodrugs

One of the main limitations of the therapeutic application of nucleoside-based drugs is the low rate of the 5′-monophosphorylation step. Taking this into account, the approach of recruiting monophosphates of modified nucleosides was proposed, but was hindered by the limited catabolic stability of nucleotide antimetabolites [113]. Replacing the 5′-phosphate group with a 5′-phosphonate group facilitated greater metabolic stability and prolonged the action of nucleotide-like drugs (cidofovir **61**, adefovir **62** and tenofovir **63**, Table 2) [114].

Cidofovir (**61,** Vistide^®^, *S-*HPMPC) is a cytosine derivative that contains an acyclic fragment with a free primary hydroxyl group and displays activities against a wide range of DNA-containing viruses (i.e., polyoma, papilloma, adeno-, pox-virus, herpes simplex virus (HSV, type 1 and type 2), varicella zoster virus (VZV), human cytomegalovirus (HCMV) and EBV). This drug is mainly used to treat HCMV retinitis in AIDS patients, but it is also a drug that can be used as short-term smallpox prophylaxis against biological weapons [115,116]. Unfortunately, cidofovir, like almost all of the acyclic nucleoside phosphonates, is associated with serious side effects due to its toxicity; as such, its use is limited to short-term therapy. Adefovir (**62**, PMEA) was used to treat patients with HIV, but it exhibited any acceptable results only at sufficiently high doses. Also, they demonstrate significant advantages in biological activity against viruses, the use of cidofovir and adefovir is accompanied by a number of side effects, including nephrotoxicity and impaired renal function. One of the most widely used phosphonate nucleoside drugs is tenofovir, discovered by Erik DeClercq and Antonín Holý’s research groups (**63**, *R*-PMPA). It is an adenosine analogue used for the treatment of HIV as well as chronic HBV infections [114,120]. To increase its bioavailability, the method of prodrug development was used. The well-known prodrugs were tenofovir disoproxil fumarate (**64**, Viread^®^, TDF) and tenofovir alafenamide (**65**, TAF), which had displayed greater efficacy in anti-HIV therapy compared to tenofovir [119,120,121,122]. In the docking studies, tenofovir 5′-triphosphate was shown to interact with HIV and HBV reverse transcriptase in quite a similar manner to 3TC, with the Lys residue forming an ionic bond with the γ-triphosphate group. This contact is probably one of the most important interactions, as the known drug-resistant mutations disrupt ionic contacts with the phosphate group [107]. In analogous mechanisms of interaction, the combination of tenofovir with the 3TC analogue emtricitabine has been proposed for combined antiviral therapy, as has been done for the drug Truvada, which is commonly used in medicinal practice for the treatment of HIV [123].

In addition to antiviral activity, several other acyclic nucleoside phosphonates, including nucleoside analogues containing the 9-(3-hydroxy-2-phosphonylmethoxypropane) fragment, such as HPMPA derivatives (Table 2), have displayed potent activity against various types of *Trypanosoma*, which causes African sleeping disease. The most effective of the nucleoside derivatives appeared to be adenosine analogues containing 9-(3-hydroxy-2-phosphonylmethoxypropane) **66**, especially the *S*-enantiomer ((*S*)-HPMPA), which demonstrated strong activity against *T.b. rhodesiense* with an EC_50_ of 0.028 µg/mL [124].

### 3.3. Chiral Acyclic Fragment Mimicking Pentafuranose Functionality

**Scheme 2:**
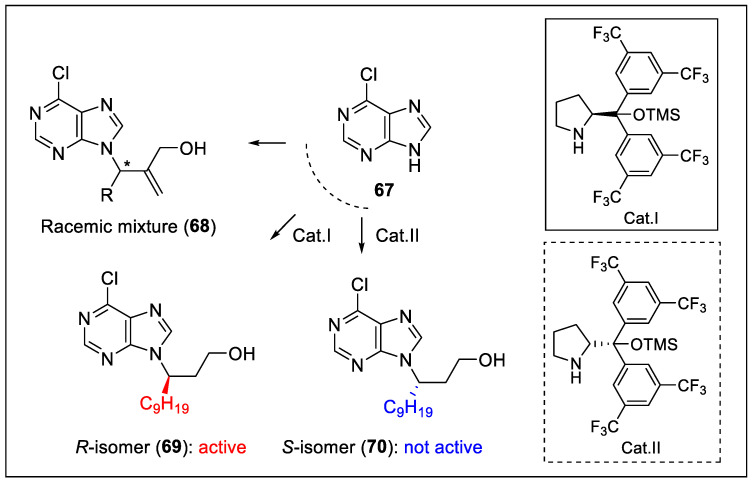
Synthesis of some chiral purine derivatives bearing a linear fragment.

A series of novel lipophilic acyclic nucleoside analogues was later developed by Hao and colleagues (2021) [125] to elucidate the effect of stereochemistry on anticancer activity (Scheme 2). In the constructed compounds, various alkyl groups or a C=C double bond at the C2′ position in the side chain was introduced at the C1′ position to form a chiral center. The C2, C6, and C8 positions of the purine ring were also modified with other functional groups.

The first group of compounds was obtained *via* the asymmetric Michaelis addition of the purine base to α,β-unsaturated aldehydes in the presence of *R*- or *S*-prolinol (Cat. I and II) (Scheme 2). The introduction of a double bond near the chiral center was achieved *via* the condensation of a purine with Morita–Baylis–Hillman (MBH) adducts.

Studies of antitumor activity on several cell lines have shown that introducing a linear alkyl substituent R in purines at the C2, C6, and C8 positions has a moderate effect on their antitumor activity. Increasing the length of the alkyl chain at the C-9 position initially led to an increase in anticancer activity, followed by a decrease upon further elongation. Derivatives with the *R*-configuration at the C1′ position of the alkyl chain exhibited several-fold higher anticancer activity than their C1′ optical antipodes, while the presence of an olefin double bond at the C2′ position of the acyclic side chain decreased antitumor activity [125].

3(*R*)-(6-Chloro-9H-purine-9-yl)Dodecane-1-ol (**69**) was selected from the synthesized compounds as the most active analogue against HCT-116 and SW480 colon cancer cells, with IC_50_ values of 0.89 and 1.15 μM, respectively. It was shown that the lead compound induced cell apoptosis, as evidenced by a significant increase in the expression of pro-apoptotic proteins Bax and p53 and the protein fragment following caspase cleavage of PARP. In vivo studies have shown that **69** can inhibit tumor growth and cause tumor necrosis, with no effect on the body weight of mice, indicating powerful antitumor activity and low toxicity. The LD_50_ value of this compound in vitro was comparable to the IC_50_ value of 5-fluorouracil (5-FU), which was selected as a control compound; its metabolic stability has also been assessed [125].

## 4. Structure–Activity Relationships 2: Base Modification and Combined Approaches

### 4.1. Chiral Hydrocarbon Substituent

It was shown earlier that natural compounds, *N*^6^-furfuryladenosine (**71**, KINR), *N*^6^-isopentenyladenosine (**72**, IPR), and *N*^6^-benzyladenosine (**73**, BAR) are active against enterovirus A71 (EVA71) and several stems of poliovirus and also exhibit anticancer activity as well [126,127,128,129,130,131].

**Figure 6:**
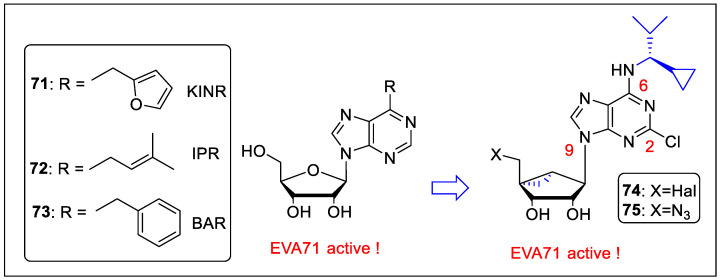
Anti-enteroviral activity of BAR and its synthetic analogues.

Replacing the ribofuranose ring with the conformationally rigid methanocarbacyclopentane and the phenyl group with chiral hydrophobic fragments at *N*^6^, as proposed by Tosh and colleagues, did not appear to enforce antiviral effect, which was 10 times lower compared to that of BAR [132]. Highly hydrophobic alkyl groups at *N*^6^ lowered the inhibitory potency, possibly due to steric hindrances, which prevents an interaction of small molecules with target-binding regions in viral NA-converting enzymes. Replacing the primary hydroxyl group in the methanocarba core with either an azido or a halogen substituent, combined with the introduction of a compact chiral fragment, resulted in compounds **74**–**75**, which exhibited consistently high and reproducible antiviral activity. These compounds displayed a *ca.* 8 times lower cytotoxicity compared to naturally occurring BAR derivatives (Figure 6).

According to the proposed SAR-model, it can be speculated that hydrophobic phenyl(alkyl) groups at *N*^6^ in combination with the bulky substituents at the position 5’ of the carbocyclic residue reduce antienteroviral activity. More effective inhibition of EVA-replication is observed for the combination of a branched chiral substituent and a 5’-truncated carbocyclic residue [132].

### 4.2. Amino Acid Conjugation

In addition to prodrug + nucleotide (“PROTIDE”) technology, the introduction of a chiral amino acid into a heterocyclic base remains an area of interest with respect to an increased affinity, which is due to multipoint fixation on target proteins. This approach was hypothesized to circumvent cytotoxicity, especially for nucleoside inhibitors of RTs [133]. As was previously shown in earlier research, acylation of atypical nucleoside drugs with hydrophobic amino acids (Val-acyclovir, Val-gancilovir, etc.) increased their bioavailability by nearly 1.6 times compared to unmodified drugs [134,135,136]. Previously reported amino acid derivatives of purine ribosides also manifested AR-stimulating and antiviral activity [137].

**Figure 7:**
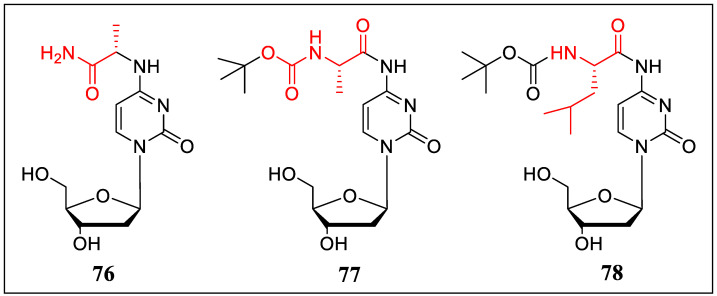
Chemically modified deoxycytidine derivatives.

In the later studies by Koplūnaitė and colleagues (2024), increased enzymatic activity of kinases DmdNK and BsdCK towards properly modified cytidine nucleosides was observed [138]. These compounds (**76**–**78**) contained the lipophilic, amino acid-like, chiral substituents at the position *N*4 of the cytosine base (Figure 7).

The primary amide group in *N*4-alanioyl-2′-deoxycytidine (**76**) was shown to be actively phosphorylated by mutant enzymes DmdNK and BsdCK. Investigations show that introducing of a compatible chiral substituent may broaden the substrate specificity of nucleoside kinases. Considering this strategy, some *N*4-substituted cytidine derivatives may be used as prodrugs, targeting the nucleosidekinase DmdNK or HSV1-TK in cancer cells; these enzymes are key components applied in suicide gene therapy [139,140,141].

### 4.3. Other Enzymatic Reactions

Nucleoside-converting enzymes participate in salvage pathways to restore NA balance and hence can also bind to chemically modified nucleoside substrates. One of the possible pathways of the enzymatic depletion in nucleoside-based drugs is the cleavage of the *N*-glycoside bond. The reverse biosynthetic process is possible, which is of importance for the development of novel selective approaches to synthesize nucleoside-based biologically active compounds.

*N-*glycoside bond formation through chemical procedures is a key step in nucleoside synthesis. This synthetic step requires the use of Lewis acids (SnCl_4_, TiCl_4_, and TMSOTf) as catalysts, which, in the case of 2′-deoxynucleosides, leads to the formation of a α/β-anomeric mixture [142,143]. In the case of arabinonucleosides, α-anomers (1,2-*trans*-configuration) are presumably formed. Enzymatic methods of *N*-glycoside bond formation can therefore compete with chemical glycosylation, as they offer more a regioselective approach and reduce the number of synthetic steps (Table 3). The use of enzymes as catalysts also represents an eco-friendly approach that avoids some poisonous reagents and solvents used in chemical synthesis.

**Scheme 3:**
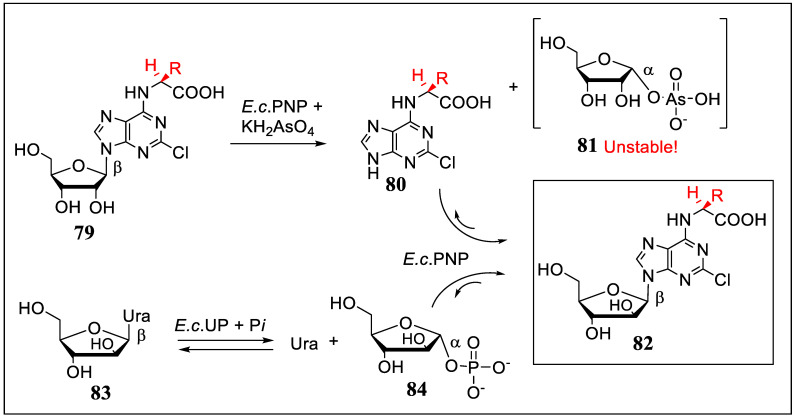
One−pot enzymatic synthesis of purine arabinonucleosides bearing amino acid residues.

Nucleoside phosphorylases (NPs) are widely used in the synthesis of nucleosides *via* a glycosylation of heterocyclic bases with pentose-1-phosphates or *via* a trans-glycosylation procedure, where the source of pentose-1-phosphate (1-α-D-arabinofuranosylphosphate, α-Ara-1-P, **84**) is a nucleoside (Scheme 3).

Nucleoside-converting enzymes strongly differ in their substrate specificity; therefore, bacterial enzymes with broader substrate specificity toward nucleosides and bases are preferable for use in synthetic procedures, leading to the desired products with various structures. Nucleoside phosphorylases from bacteria also convert arabinonucleosides with selective formation of the 1,2-*cis* configuration at the C1′-C2′ bond. This striking ability may provide an opportunity to increase the availability of expensive nucleoside drugs. The chemo-enzymatic synthesis of purine ribosides and deoxyribosides with chiral substituents and its stereoselectivity have been described in our earlier review [56]. This procedure has been successfully expanded to 9-arabinofuranosyl-2-chloropurines bearing an amino acid chiral moiety (**82**) to generate potential antiproliferative compounds (Scheme 3). In the study by Konstantinova and co-workers [147], an original one-pot method was proposed, starting from Ara-uridine (**83**) as a glycosyl donor to form α-Ara-1-P in the presence of uridine phosphorylase (*E.coli* UP). The initial *N*^6^-modified 2-chloroadenosine (**79**) was cleaved by purine nucleoside phosphorylase (*E.coli* PNP) in the presence of catalytic amounts of sodium arsenate to form a purine base as a glycosyl acceptor. These conditions shifted the equilibrium toward the formation of purine arabinose nucleosides with preferentially high yields.

Enzymatic transglycosylation usually involves two enzymes, the first cleaving the pyrimidine glycosyl donor and the second converting the purine base into the corresponding nucleoside. Optimization of the reaction conditions by replacing two enzymes with one, which would convert both the glycosyl donor and glycosyl acceptor in the case of NPs, implies the use of specifically modified substrates. It was discussed earlier that 7-Me-(deoxy)guanosine can be used as a source of (deoxy)ribose-1-phosphate in trans-glycosylation reactions due to its practically irreversible phosphorolysis. This was demonstrated for the first time in the enzymatic synthesis of the drug tecadenoson, catalyzed by *Aeromonas hydrophila* (Ah) PNP [148].

According to the novel experimental data, replacing NPs with *Lactobacillus leishmanii* deoxyribosyltransferase NDT II could be considered as an alternate, potent method for synthesizing of modified nucleosides *via* the trans-glycosylation procedure [149]. Enzymes of this class were shown to convert both purine and pyrimidine deoxynucleosides through a C1-activated deoxyribofuranose state. This procedure therefore avoids the use of labile α-D-pentofuranosyl-1-phosphates and thus can increase glycosylation yields.

To counterbalance to enzymatic synthesis, stereospecific modification of nucleoside derivatives can be considered as a strategy for drug development, through which the structure of native substrates and their binding capacities for enzymes of the salvage pathway are mimicked; this approach has been described for the 2,2′-*O*-cyclonucleoside inhibitor **89** of uridine phosphorylase and kinase [150].

**Scheme 4:**
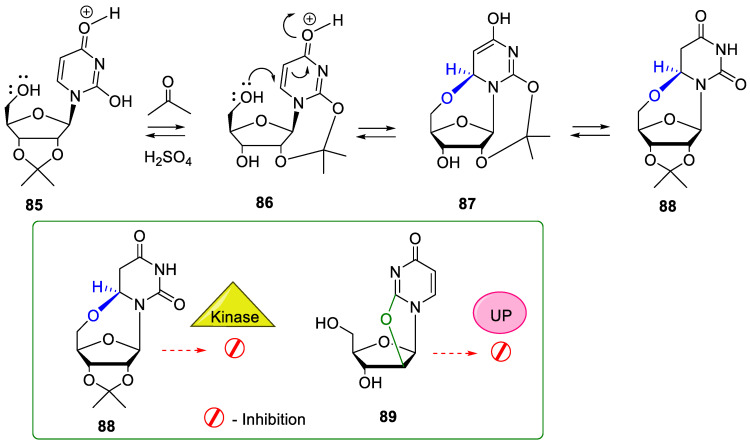
The formation of a cyclic nucleoside structure with a chiral center in the pyrimidine base.

An analysis of the reaction ability of the uridine ketal derivative revealed regioselective formation of 5′,5-cycloadducts under Michaelis reaction conditions, with the formation of a chiral center at position 6 of the pyrimidine (Scheme 4) [151]. Considering the trapping of the 5′-hydroxyl groups in the active sites of enzymes, this may reflect biological activity of cyclic pyrimidine derivative (**88**) and its analogues, for example, as nucleoside kinase inhibitors.

Replacing the native D-ribose functional fragment with L-fragment could be considered when increasing metabolic stability, as the enzymes of the salvage pathway essentially do not cleave L-nucleosides.

## 5. Structure–Activity Relationship 3: Prodrugs and Bioavailability

Selective binding plays a key role in inhibiting metabolic processes of nucleic acids and their components. Nucleoside(t)ides containing a pentafuranose fragment in the D-configuration dominate in the composition of natural NAs and thus exhibit better affinity to nucleoside-converting enzymes. However, it has been shown that some L-nucleosides can exhibit comparable biological activity (see Table 1), a more favorable toxicological profile and a greater metabolic stability [21,86,88,95]. Therefore, L-nucleoside based drugs are of interest as long-acting medications. Unfortunately, an “unnatural” configuration of a carbohydrate moiety could decrease bioavailability due to lower affinities to some cellular proteins. The general mechanism of action of Ara- and *C*-methylnucleosides and L-derivatives also appears to be based on their intracellular phosphorylation. A small, “one-point” modification can preserve their affinity to cellular and viral enzymes. Viral enzymes are characterized by widened substrate specificity, and the replacement of D-ribose with the L-ribose can decrease affinity to host protein receptors while conserving antiviral properties due to phosphorylation. The stereoselective modifications of nucleoside molecules producing various biological effects are schematically summarized in Table 4.

Many nucleoside compounds, given in Table 1 and Table 2, especially those in the D-configuration, display cytotoxic effects, pure cell penetrability or low chemical stability. These factors decrease their bioavailability and restrict penetration through the blood–brain barrier. In order to increase bioavailability, selective drug delivery systems have been proposed and explored.

**Figure 8:**
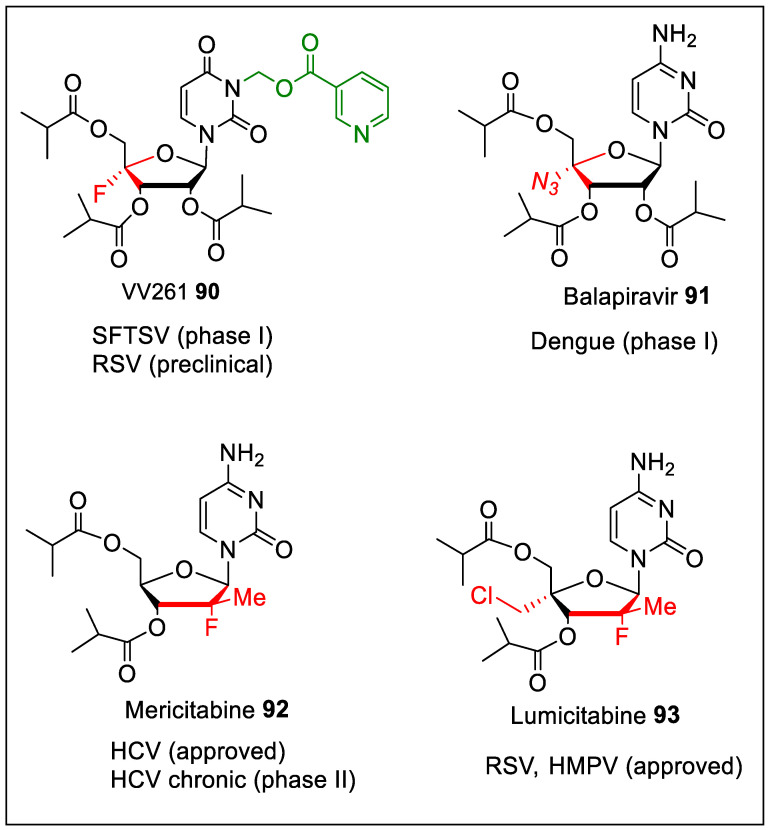
Isobutyroyl drug delivery system.

Various modifications may strengthen the chemical stability and bioavailability of some halogenated nucleoside compounds, as was recently shown for the 4′-fluorouridine prodrug VV261 (**90**) [152]. This prodrug contains three *O*-isobutyroyl groups, which are easily hydrolyzed by cytosolic esterases, and a nicotinoyloxymethyl group at the uridine position *N*3, capturing nucleoside inside the cell. According to the recent literature data, VV261 exhibits potent anti-SFTSV activity and has entered Phase I clinical trials in China for the treatment of SFTSV-induced infection [152]. It also displays anti-CHIKV-efficacy [153]. Balapiravir, an analogue of VV261, is active against dengue fever virus. Other examples include modified analogues of 2′-fluoro-2′-*C*-methyl-2′-deoxycytosine (**9**): mericitabine **92**, an isobutyroyl derivative of **9**, has remarkable activity against HCV and has been approved for medical practice, and Lumicitabine **93**, the 4′-C-chloromethyl derivative of mericitabine, has been approved for the treatment of RSV and HMPV-induced infections and suppressed RSV replication [26] (Figure 8).

**Figure 9:**
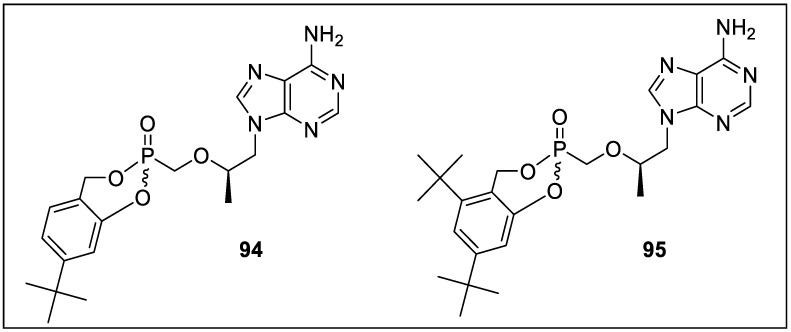
Cyclophosphonate drug delivery system.

To increase the therapeutic potential, several phosphoryl/phosphonate prodrugs have been discovered, that enzymatically convert to their di- and triphosphate forms. Nucleotides and nucleoside phosphonates are polar molecules: they cannot easily pass through the cellular membrane. Therefore, new drug delivery systems are needed to circumvent the unfavorable pharmacokinetic properties of the phosphate and phosphonate groups. To elucidate the mechanism of pH-driven nucleotide delivery into a cell, the *cyclo*-saligenylphosphate delivery system, masking charged groups, was proposed [154]. This delivery system is based on a neutral phosphate groups modified with a lipophilic membrane permeable *cyclo*-saligenyl (*cyclo*-Sal) residue, which is structurally related to acetylsalicylic acid. Depending on the functionality at its aromatic fragment, *cyclo*-Sal phosphotriesters **94**–**95** can selectively deplete inside a cell to form free mononucleotides. Using this approach, some effective phosphonate derivatives, with high anti-HIV activity have been identified (Figure 9). Nevertheless, they are less active than the corresponding analogues, tenofovir and its diisopropoxyl fumarate, but do possess an anti-HIV activity comparable with that of adefovir and *R*-HPMPA.

**Figure 10:**
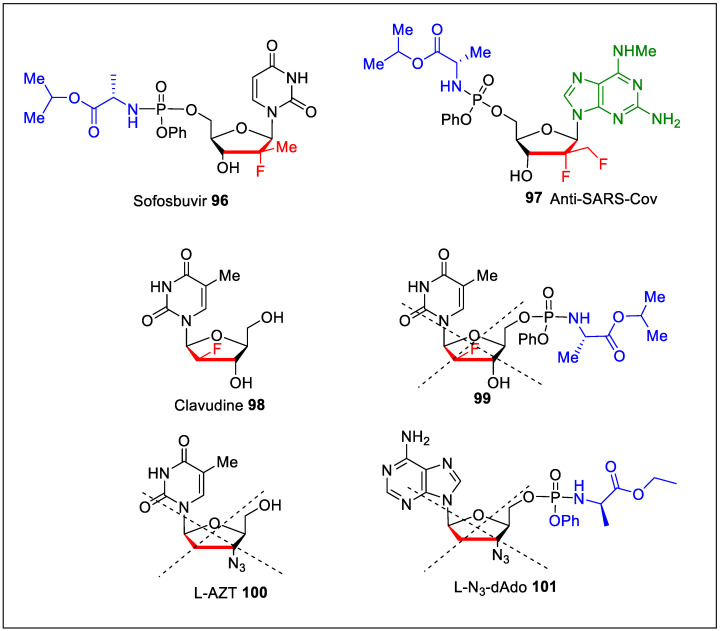
PROTIDE drug delivery system.

Considering the need to fight high viral mutability, PROTIDE technology comprises an efficient approach for a rapid elaboration of selective nucleoside-based drugs acting on viral replication in an RNA-inhibiting manner (Figure 10).

Using this technology, two antiviral drugs have been discovered—sofosbuvir (**96**) for the treatment of hepatitis C and remdesivir for the treatment of MERS and SARS-CoV2 [14,155]. The introduction of hydrophobic amino acid residues to the 5′-phosphate group of nucleosides appears to be a promising technique due to the resulting ability to penetrate and accumulate inside the cell, where it is subjected to enzymatic cleavage in the presence of esterase (C1) and cathepsin A (CTSA), forming the corresponding nucleoside-5′-monophosphates. This is why PROTIDE-drugs are effective for the treatment of infections caused by viruses injuring cells with high expression levels of CES1 and CTSA, exemplified by the treatment of HCV by the drug sofosbuvir (**96**) in hepatocytes [156]. Quite recently, a low-toxicity PROTIDE compound **97** based on the structure of the previously reported INX-189 and sofosbuvir (**96**) was found to effectively suppress various SARS-CoV2 strains at concentrations in the submicromolar range and was tolerated in rats at high doses [157]. According to the molecular docking data, Sofosbuvir interacts with the two glutamine side residues, D761 and D760, in the active site of SARS-CoV2 RT. It also exhibits binding contacts with some hydrophobic residues [158], which may provide clues regarding interactions when considering analogous antiviral activity of **97**.

PROTIDE is not a universal approach to increasing the biological activity of every nucleoside compound. Studies have revealed that PROTIDEs based on L-AZT analogues, for example, L-N3-dA (**101**), exhibit at least 3 orders of magnitude lower anti-HIV activity compared to non-modified AZT [159]. L-AZT **100** itself possessed no activity [160]. On the contrary, a monophosphate clevudine prodrug ATI-2173 (**99**) inhibited HBV-polymerase and displayed high bioavailability and safety but was terminated on Phase 2 of clinical trials due to lack of clear regulatory pathway [26].

**Scheme 5:**
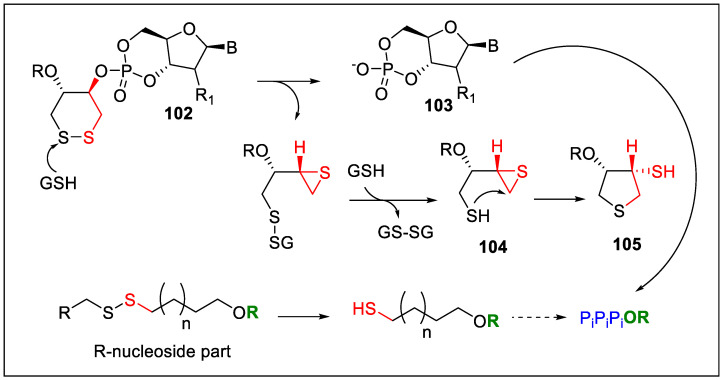
Glutathione-catalyzed depletion of disulfide-containing nucleoside chiral prodrugs in cytosol.

The glutathione-transport system is a common approach for selective drug delivery and can be used to explore modifications of nucleoside drugs of various natures. A new biosynthetic procedure, based on the glutathione transport system, has been proposed later by Butora and colleagues [161]. This strategy includes the interaction of the phosphotriester prodrug form (**102**), which contains modified cyclic disulfide functionality, with glutathione (GSH), a cytosol-specific trigger of reductive elimination (Scheme 5).

The biochemical conversion of this prodrug form was shifted toward nucleoside monophosphate by the action of NAD(P)H-dependent reductase, restoring the pool of GSH. Alternative nucleoside prodrugs bearing disulfide fragments in their lipophilic parts can be considered as drug delivery systems, acting through the glutathione oxidation/reduction metabolic pathway [156].

**Scheme 6:**
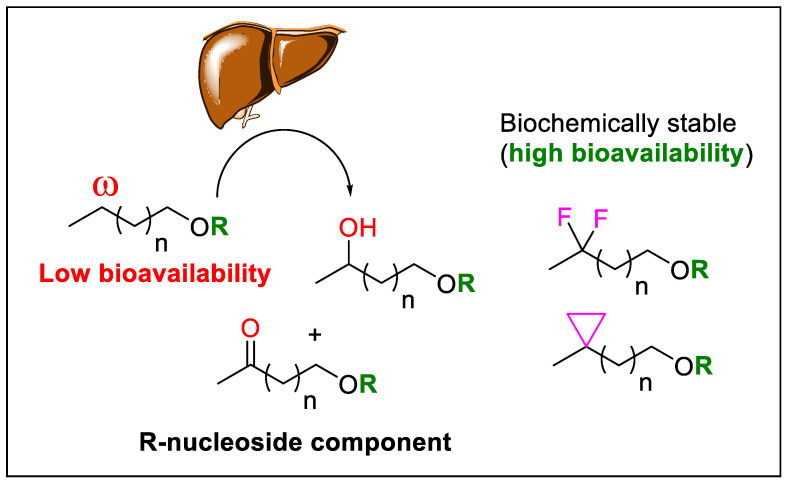
Drug oxidative metabolism in liver.

Under physiological conditions, various prodrugs are exposed to detoxification in the liver due to the omega-oxidation of their hydrocarbon moiety. This metabolic pathway significantly decreases bioavailability (Scheme 6).

To increase prodrug stability, allowing it to pass unchanged through the liver, various protection strategies *via* hydrocarbon masking groups of cyclopropenyl or difluoromethylene groups have been explored. Based on this strategy, biologically active oxidative-stable drug prototypes among acyclic nucleoside analogues can be identified [156]. Additionally, highly biochemically stable nucleic acids composed of L-nucleosides, so-called “Spiegelmers”, can attach to a range of cellular proteins and nucleic acids, and are thus being considered as potent pharmacological and drug delivery tools [88].

Some specific treatments can be identified basing on nucleoside agonists of ARs. In this case, a biological effect can be achieved by an active compound at nanomolar concentrations. The presence of a highly-lipophilic group in its structure may help them to break through the blood–brain barrier.

## 6. Conclusions

In summary, nucleoside structures represent a promising scaffold for the elaboration of novel biologically active compounds and drugs. To be an effective drug candidate, a compound, in addition to efficacy (activity), has to offer both selectivity and specificity. The former refers to a biologically active molecule’s ability to preferentially produce a particular effect, while the latter relates to the number of different effects that a drug may elicit. Practically all of the nucleoside-based drugs, along with the desired action, can cause undesirable side effects associated with cellular toxicity and metabolic detoxification in the liver and excretion by the kidneys. To increase selectivity and specificity, the introduction of chiral fragments into a nucleoside molecule or a transformation of the carbohydrate structure may be considered as a potential approach. This leads to higher selectivity towards viral enzymes, which are less specific to pentafuranose configurations. Using this strategy, some antiviral drug prototypes of L-nucleosides can exhibit comparable biological activity, a more favorable toxicological profile and a greater metabolic stability compared to widely occurring D-analogues. Several L-nucleosides display immunomodulatory potential. It has also been indicated in multiple study results that stereochemically modified nucleoside derivatives can display synergistic effects with several drugs and thereby there is potential for their use in combined antiviral or anticancer therapies to circumvent drug resistance for both drugs.

Chemical modification of the purine base with a lipophilic chiral substituent has a stimulatory or inhibitory effect on various receptors of the purinergic signaling system and can increase selectivity and/or specificity and metabolic stability. Several structurally optimized nucleoside derivatives may interact with target proteins that normally do not bind to nucleosides.

To obtain nucleoside drugs with better membrane permeability and bioavailability, several delivery systems, especially those based on chiral phosphate groups, have been successfully developed.

According to contemporary literature data, stereoselective modification of nucleosides with additional chiral groups, although not always successful, in many cases appears to be of benefit for the development of selective and specific drug prototypes. Various synthetic approaches, including enzymatic transformations and/or the introduction of specific biodegradable groups, may expand their use in clinical practice.

## Figures and Tables

**Table 1:** Comparative analysis of antiviral activity (EC_50_) and cytotoxicity (IC_50_) of D- and L-nucleoside derivatives.

L-Nucleosides				D-Nucleosides			
Compound	Virus	EC_50_, μM	IC_50_, μM	Compound	Virus	EC_50_, μM	IC_50_, μM
**29** Lamivudine (3TC) Epivir^®^ 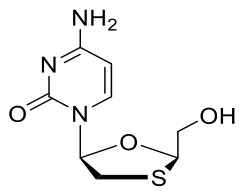	HIV-1	0.002 ^a^ [84]	>100 [83,84,89]	**30** (+)-D-BCH-189 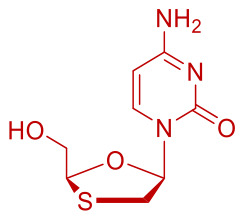	HIV-1	0.2 ^a^ [84]	2.7 ^g^ [83,84]
	HIV-2	0.02 ^a^ [83]			HIV-2	0.05 ^a^ [83]	
	HBV	0.008 ^b^ [90]			HBV	0.5 ^b^ [89]	
**31** Emtricitabine (FTC) Emtriva^®^ 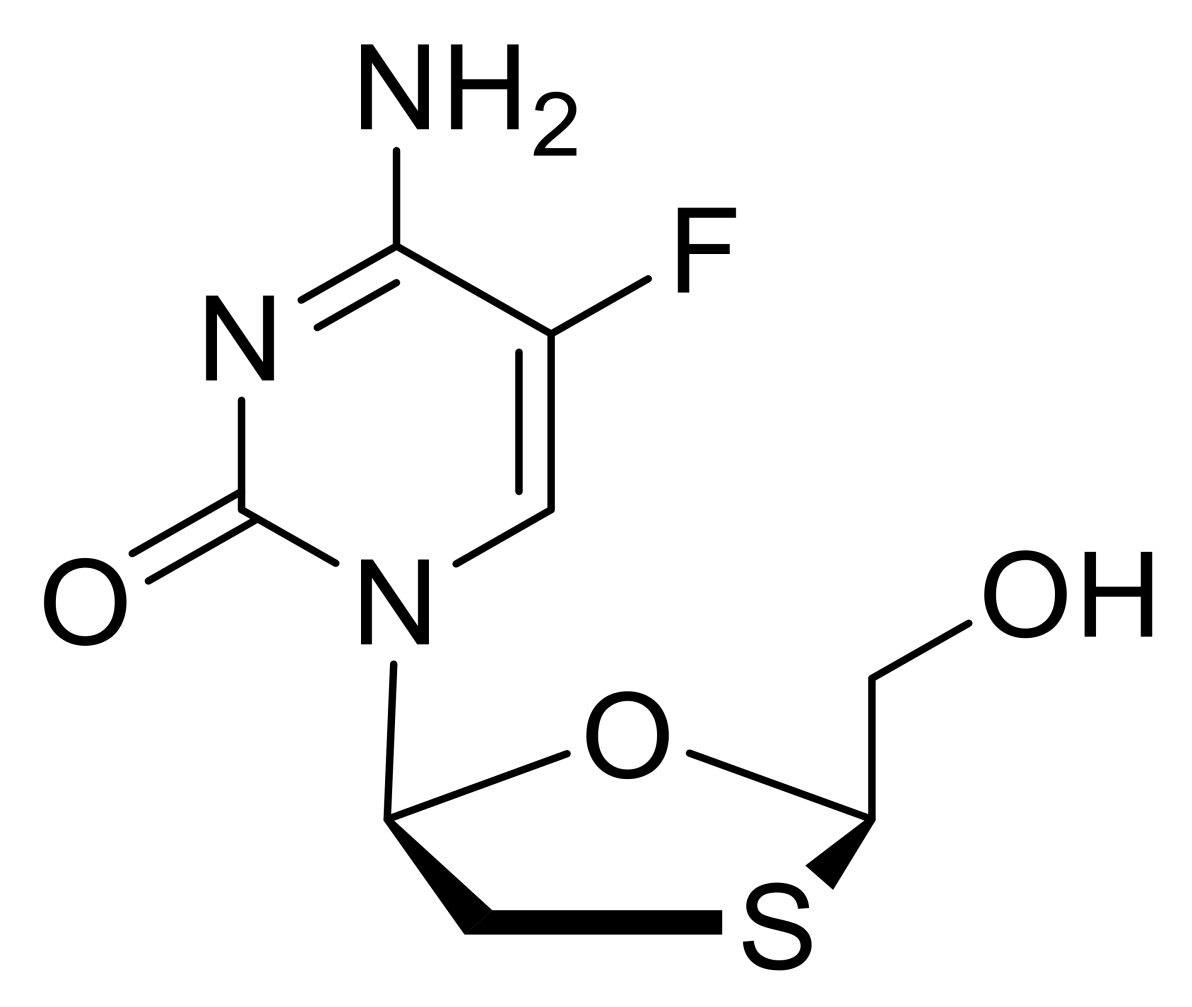	HIV-1	0.008 ^a^ [83]	>100 [83,85]	**32** (+)-D-FTC 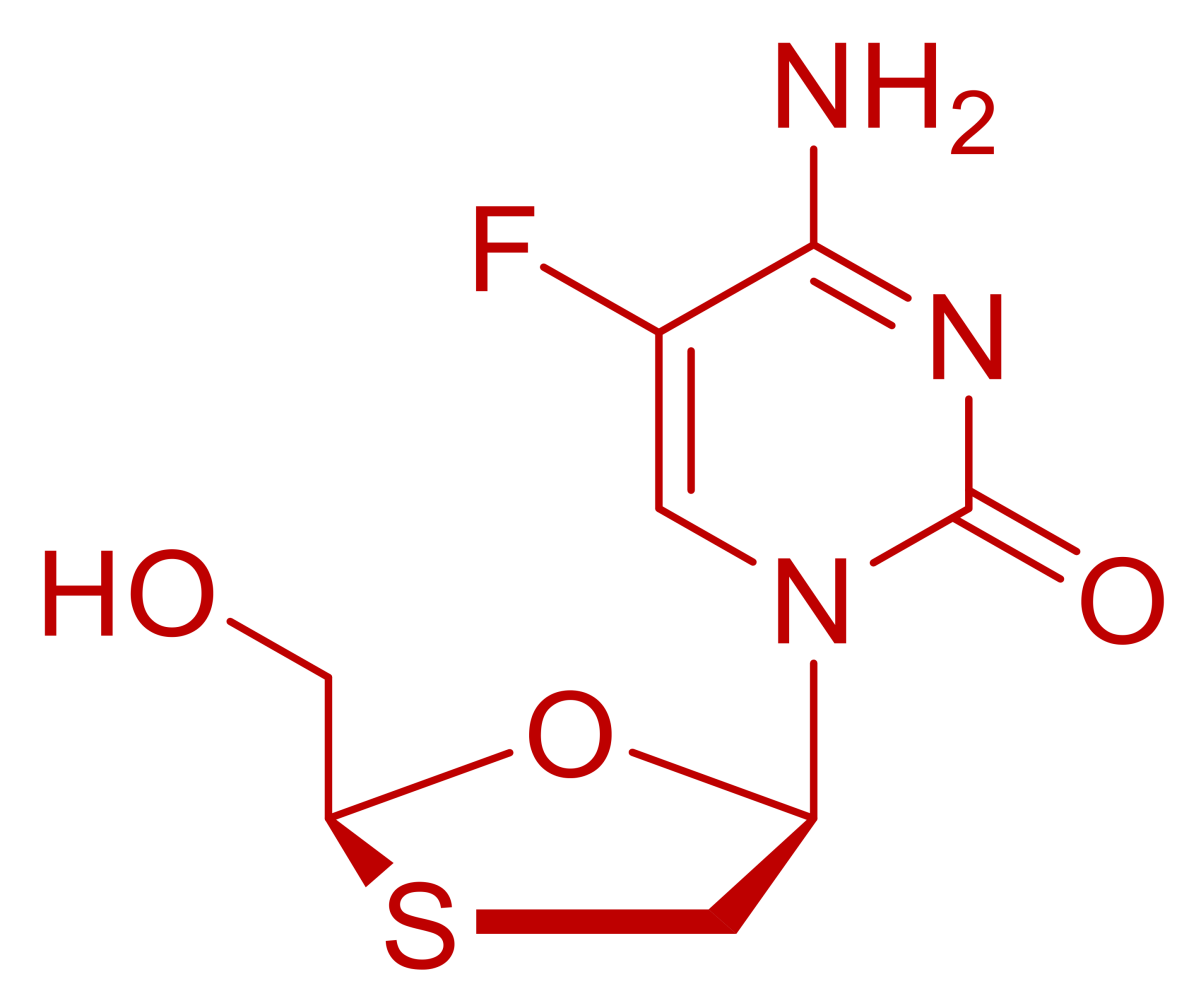	HIV-1	0.84 ^a^ [83]	>100 [83,85]
	HIV-2	0.0007 ^a^ [83]			HIV-2	0.03 ^a^ [83]	
	HBV	0.01 ± 0.005 [85]			HBV	0.96 ^b^ [91]	
**33** Troxacitabine (L-OddC) Troxatyl^®^ 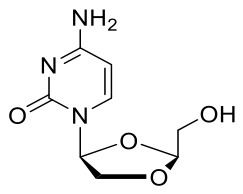	HIV-1	0.002 ^c^ [91]	0.26 ^g^ [91] 0.1 ^i^ [91]	**34** (+)-D-OddC 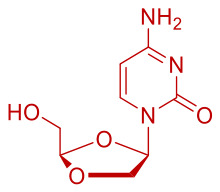	HIV-1	0.016 ^a^ [91]	12.3 ^g^ [91]
	HBV	0.0005 ^b^ [91]			HBV	0.01 ^b^ [91]	
	CA	0.060–0.3 ^d^ [92]			CA	n/d	
	HIV-1	5 [93]	17 ^h^		HIV-1	1	~30 ^h^
	HBV	0.6 [93]			HBV	6.3	
**35** L-I-OddU 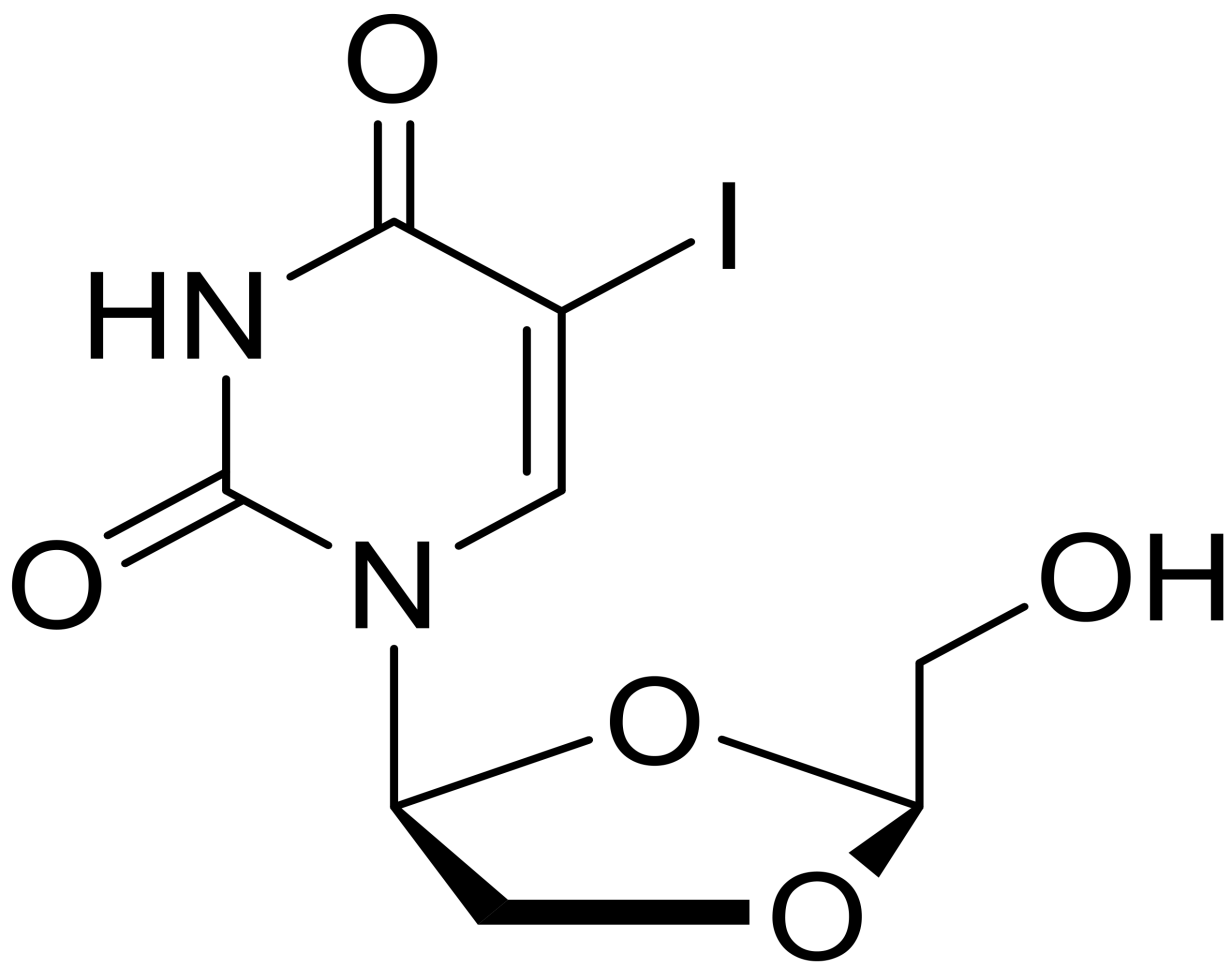	EBV	0.033 ± 0.017 ^e^ [94]	>1000 [94]	**36** D-I-OddU 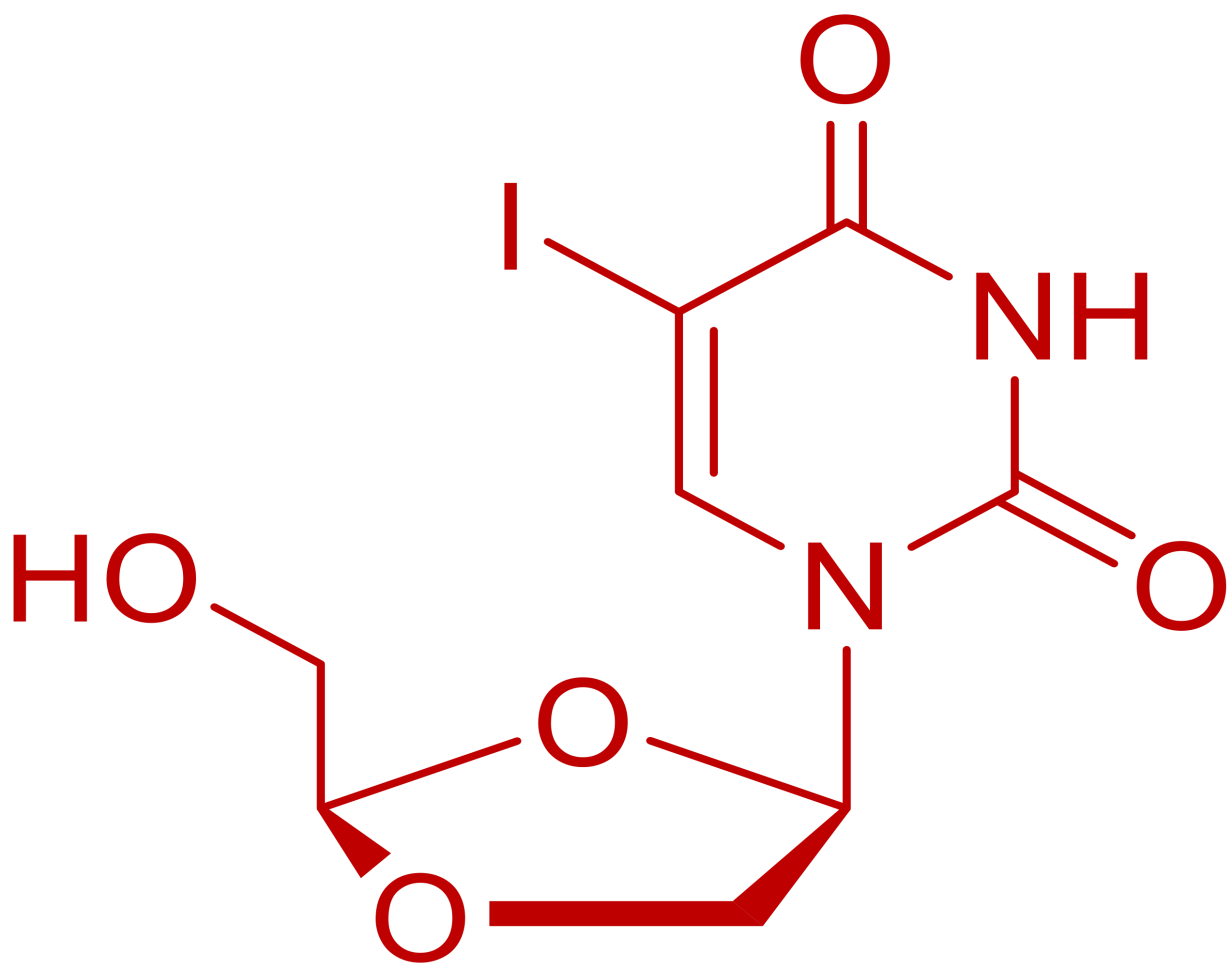	EBV	7.6 ^e^ [91]	18 ^i^ [91]
**37** Telbivudine (L-dT) Tyzeka^®^ Sebivo^®^ 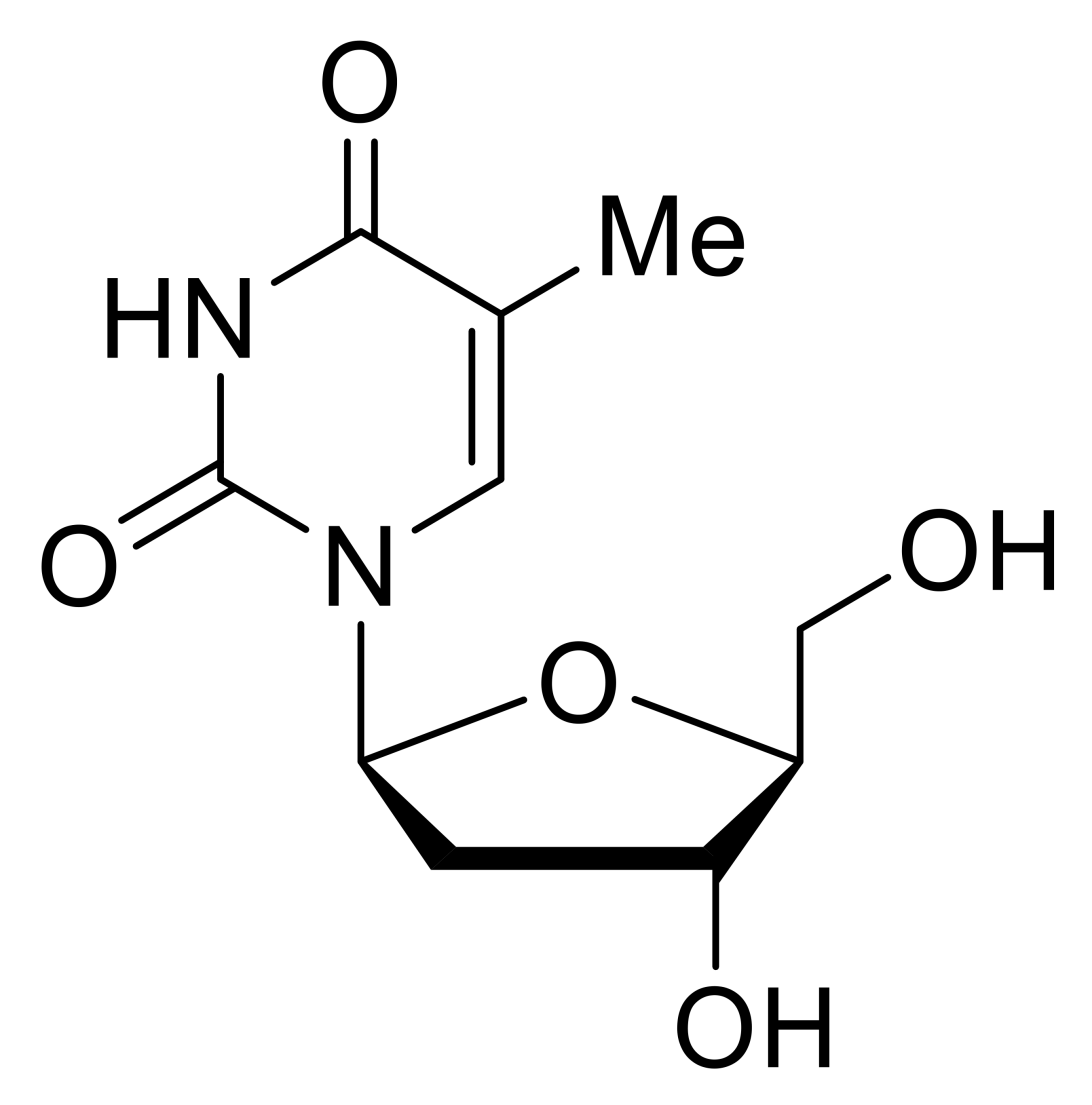	HBV	0.19 ± 0.09 ^b^ [95]	>1000 [95]	**38** Thymidine (D-dT) 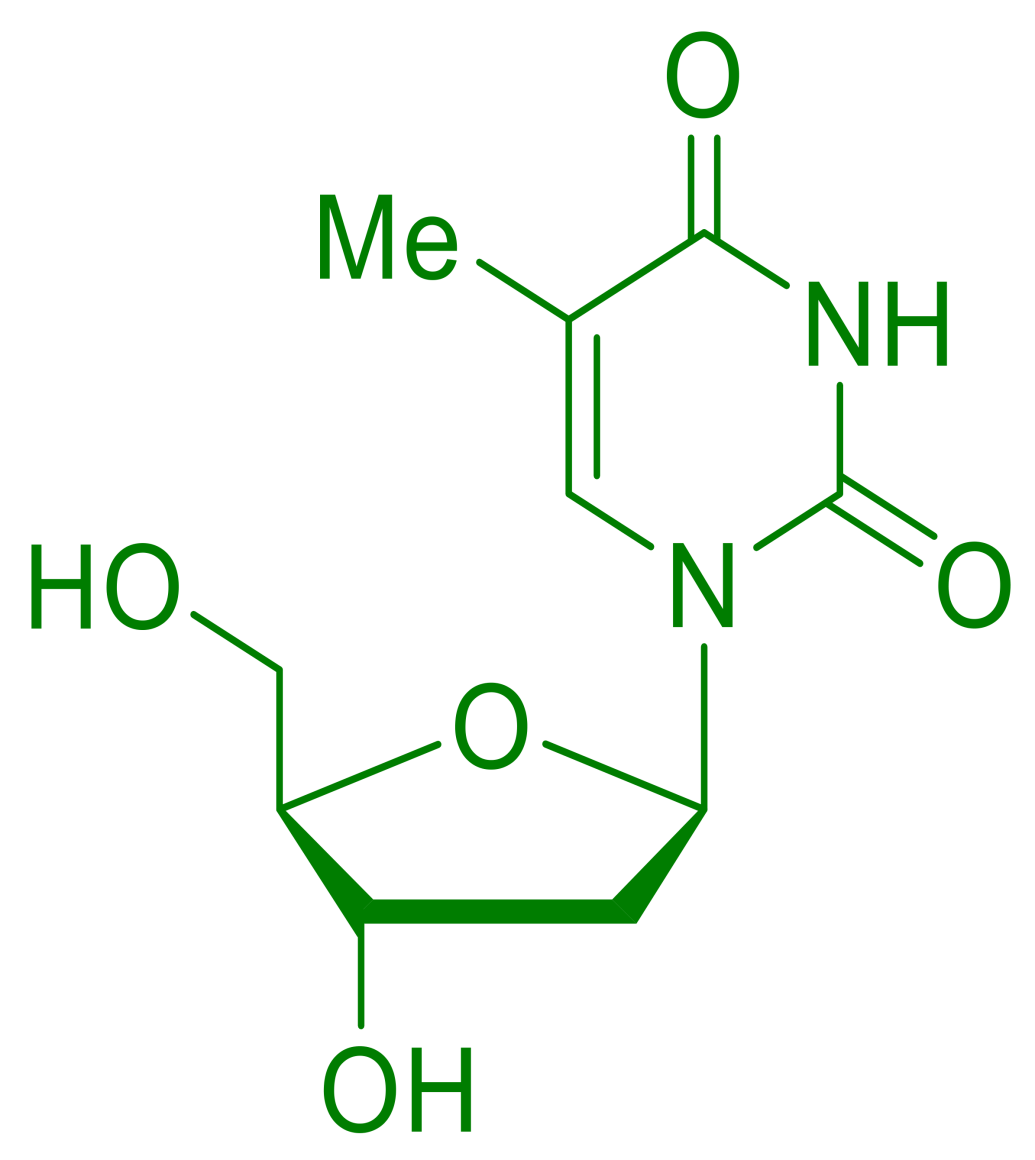	HBV	n/d	-
**39** L-dC 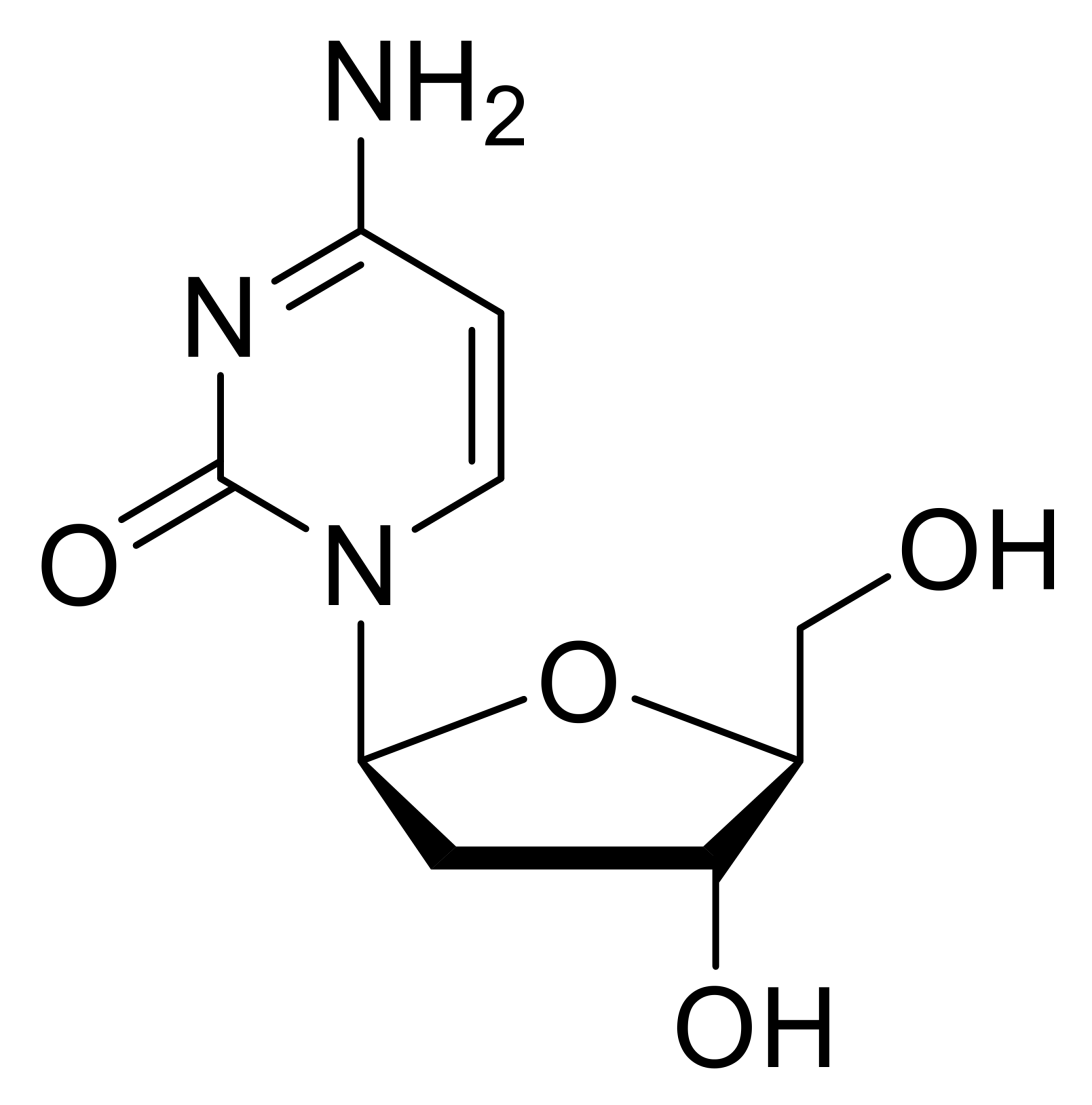	HBV	0.24 ± 0.08 ^b^ [95]	>1000 [95]	**40** 2′-Deoxycytidine (D-dC) 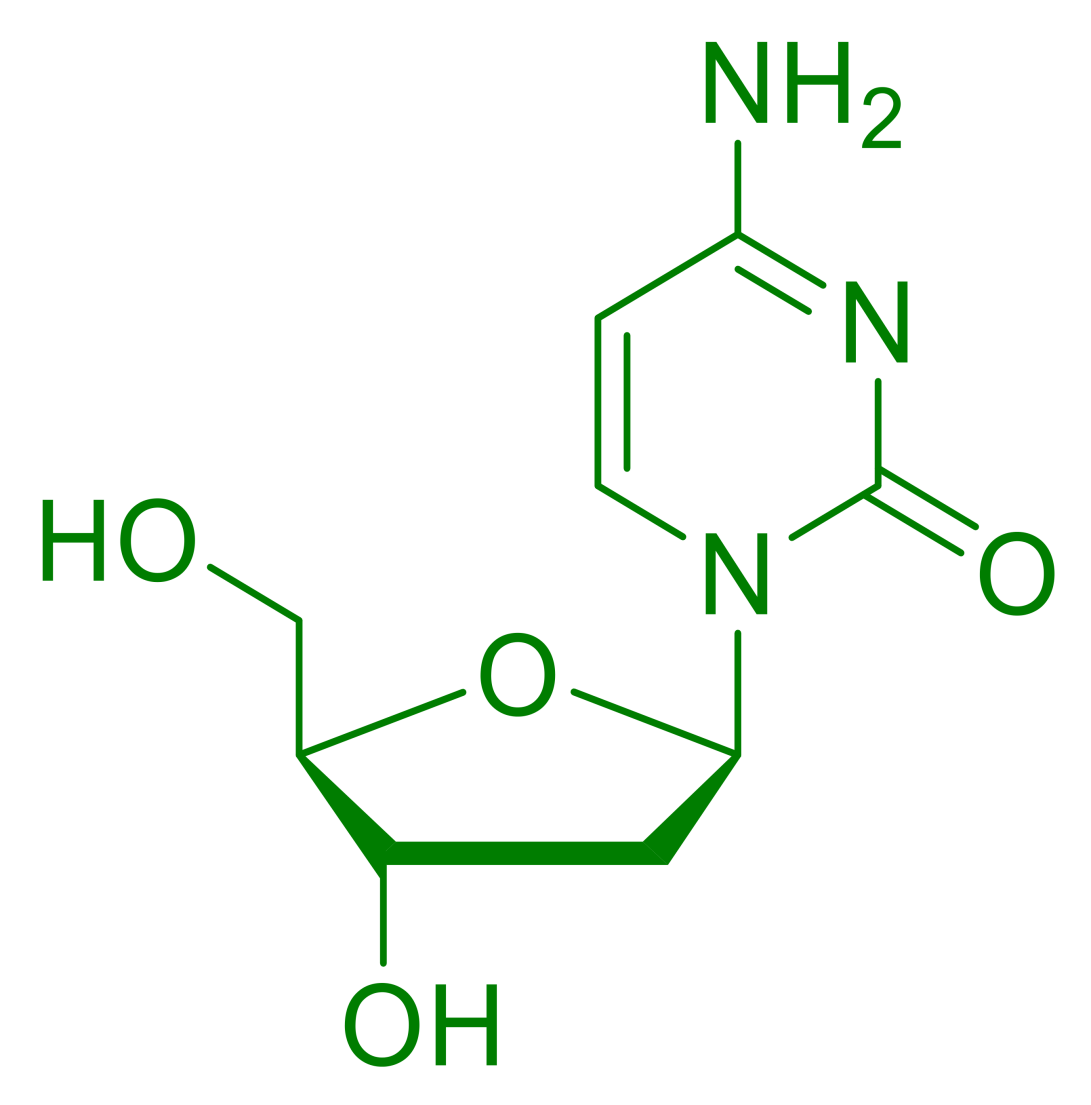	HBV	n/d	-
**41** L-d4C 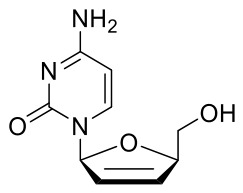	HIV	1.0 ^c^ [96]	20 ^g^ [97]	**42** D-d4C 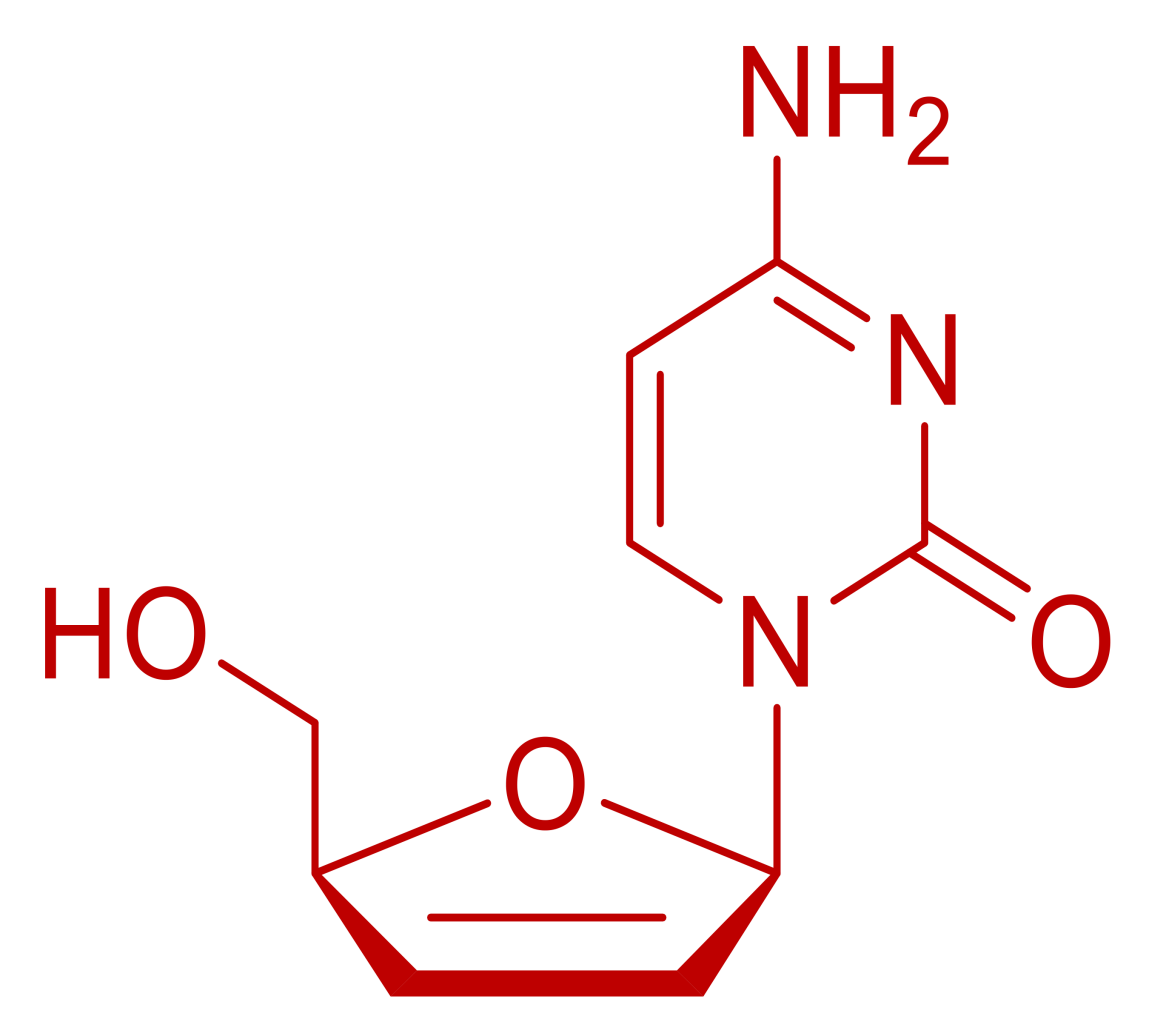	HIV	0.7 ^c^ [97]	22 ^g^ [97]
	HBV	0.008 ^b^ [96]			HBV	n/d	
**43** 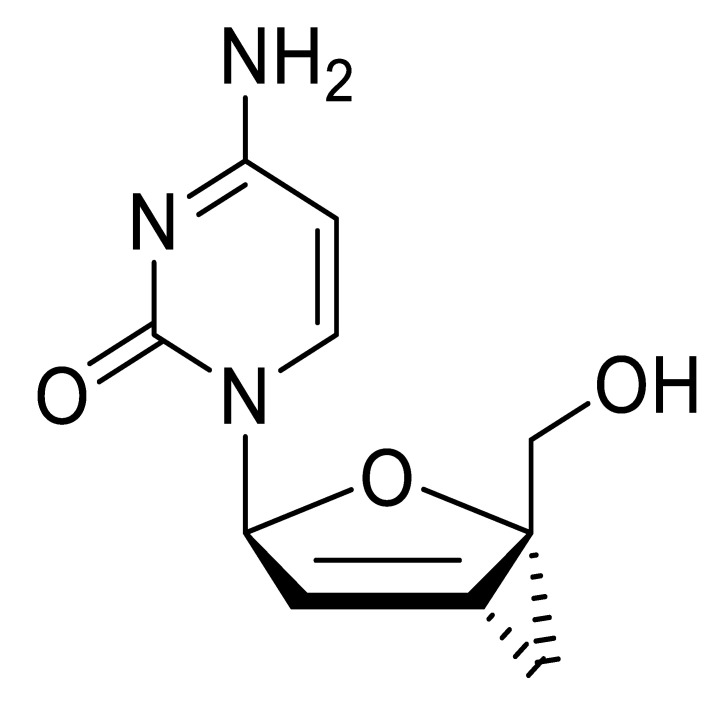	HIV-1	>400 ^d,^*	>100 [98]	**44** 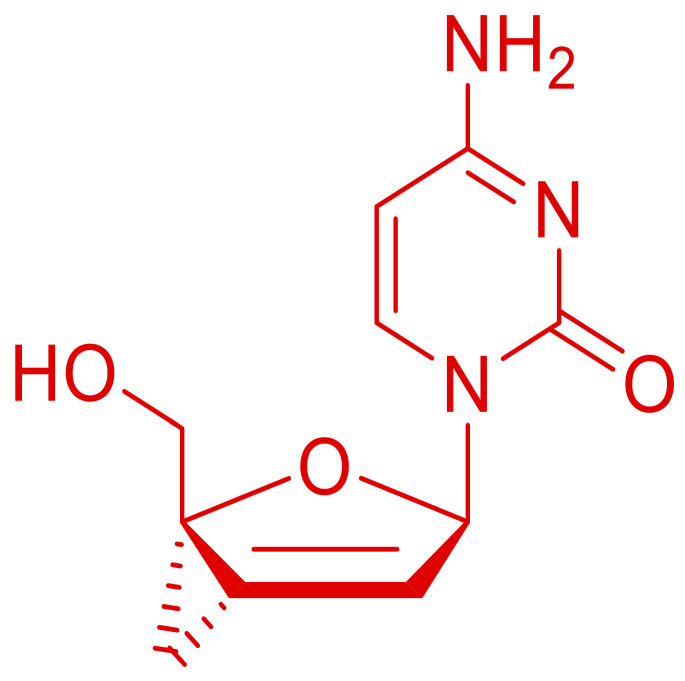	HIV-2	61.8 ^d,^*	>280 ^d,^* [98]
	HIV-2	>400 ^d,^*			HIV-2	61.8 ^d,^*	
**45** 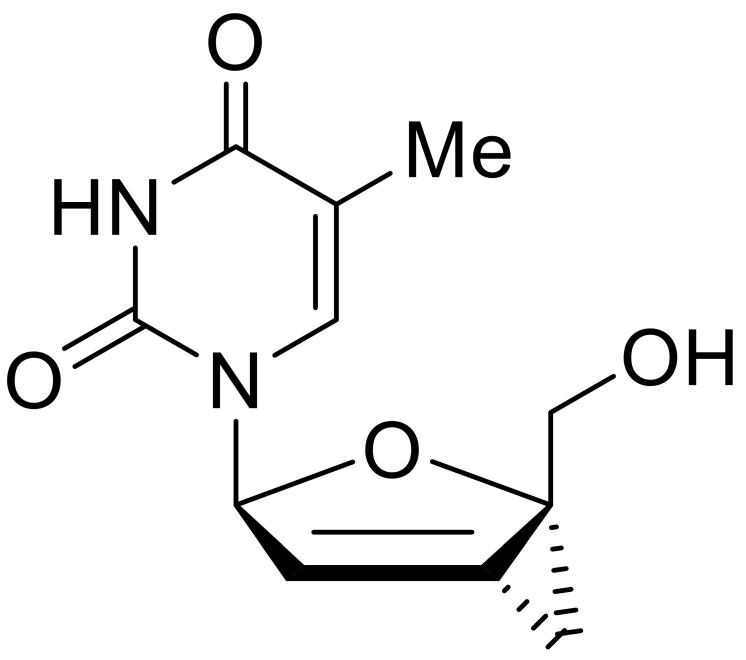	HIV-1	5 ^d,^*	>400 ^d,^* [98]	**46** 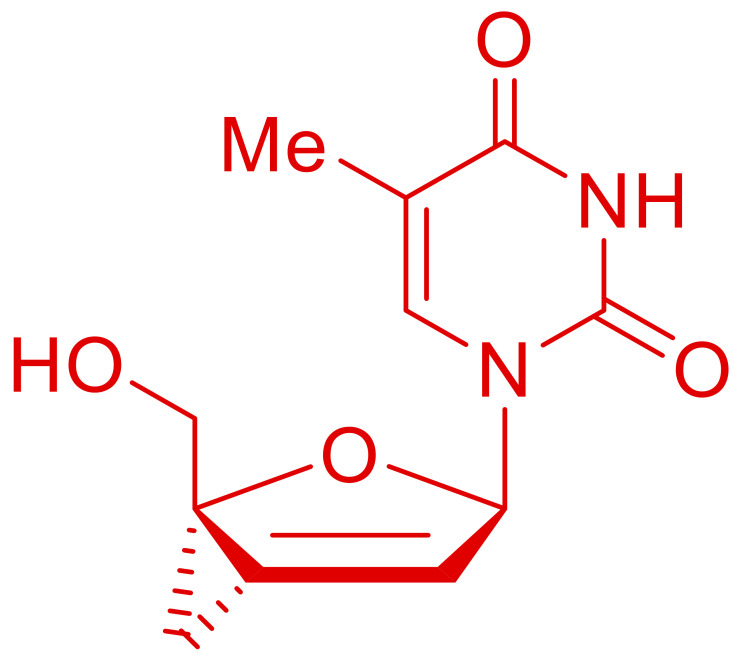	HIV-1	0.14 ^d,^*	154 ^d,^* [98]
	HIV-2	9.62 ^d,^*			HIV-2	0.474 ^d,^*	
**47** 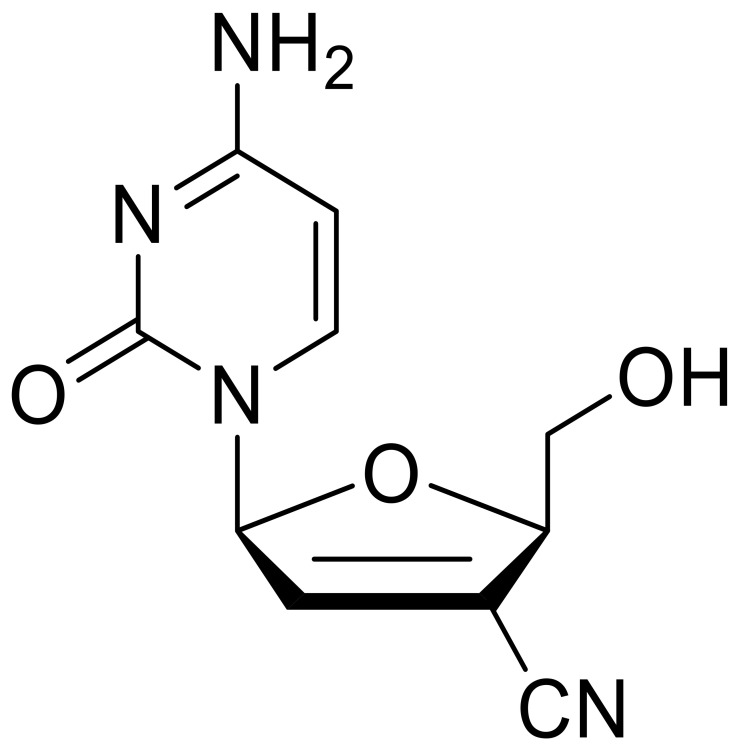	HIV	>400 ^a^ [99]	>400 ^a^ [99]	-	-	-	-
**48** 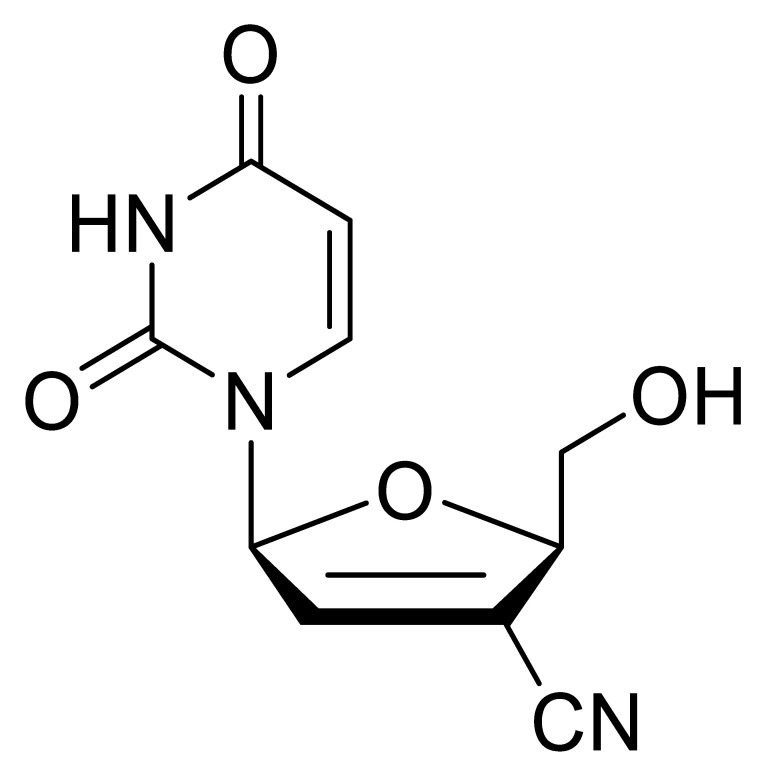	HIV	92.3 ^a^ [99]	>400 ^a^ [99]	-	-	-	-
**49** Elvucitabine (L-d4FC) 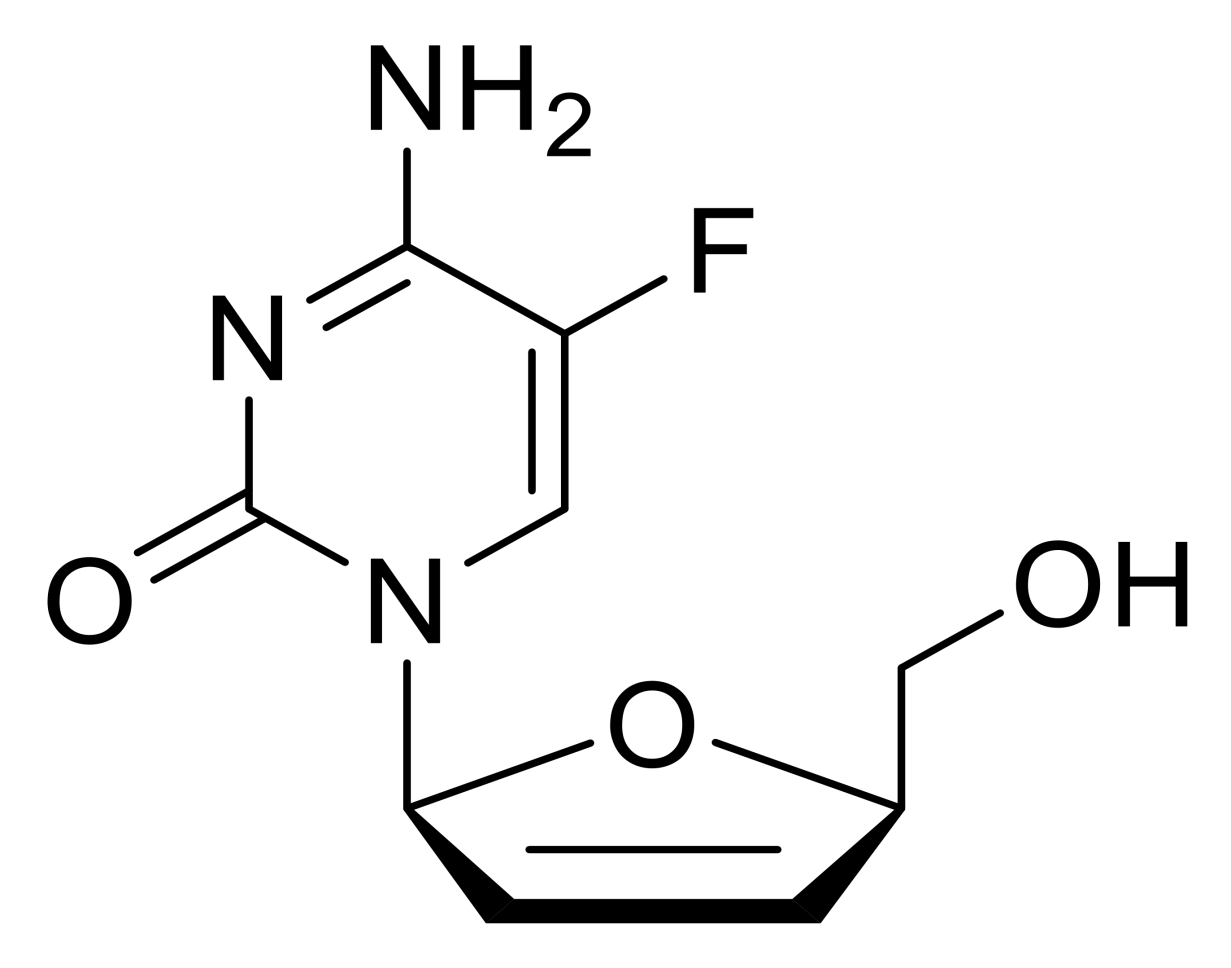	HIV	0.09 ^c^ [96]	7 ^g^ [97]	-	-	-	-
	HBV	0.002 ^b^ [96]			-	-	
**50** Clevudine (L-FMAU) Levovir^®^ Revovir^®^ 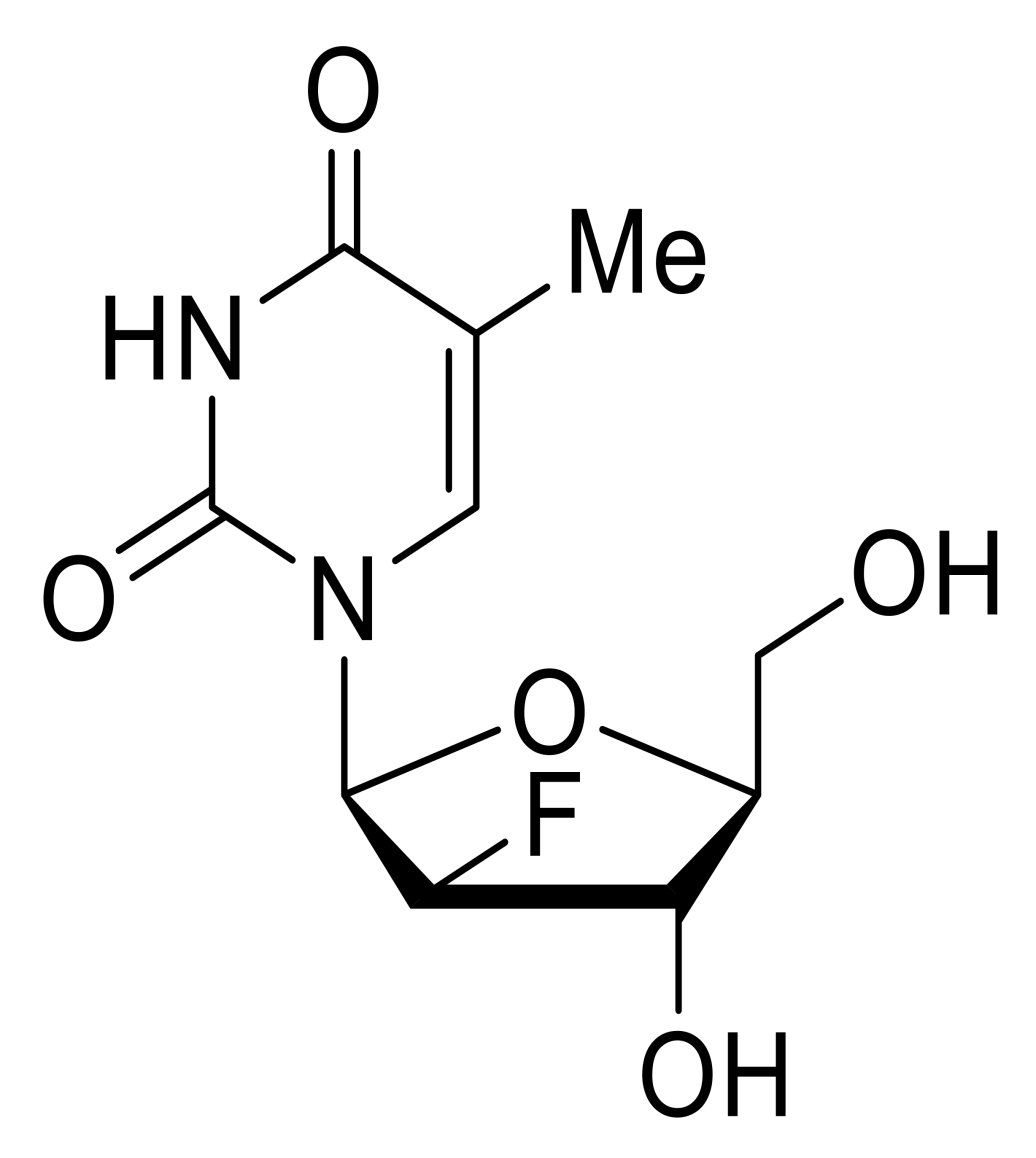	HBV	0.1 ^b^ [100]	>100 [100]	**51** D-FMAU 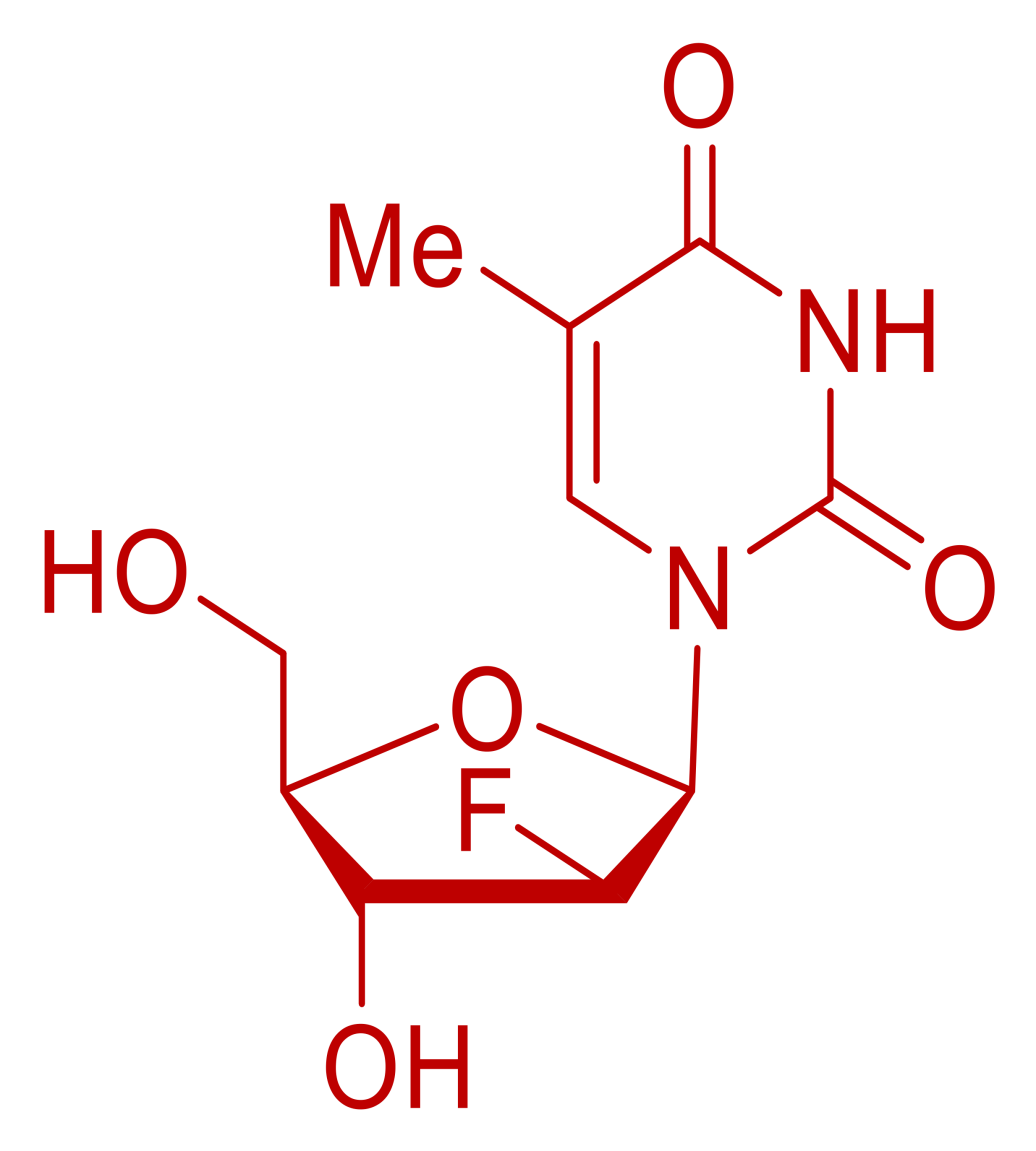	HBV	2.0 ^b^ [100]	0 ^g^ [100] <10 ^i^ [100]
	EBV	5 ± 0.8 ^f^ [101]			EBV	0.1 ± 0.02 ^f^ [101]	
**52** L-Fd4C 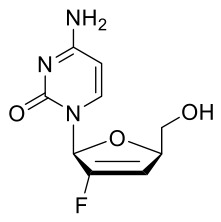	HIV	0.2 [102]	>100 [103]	**53** D-Fd4C 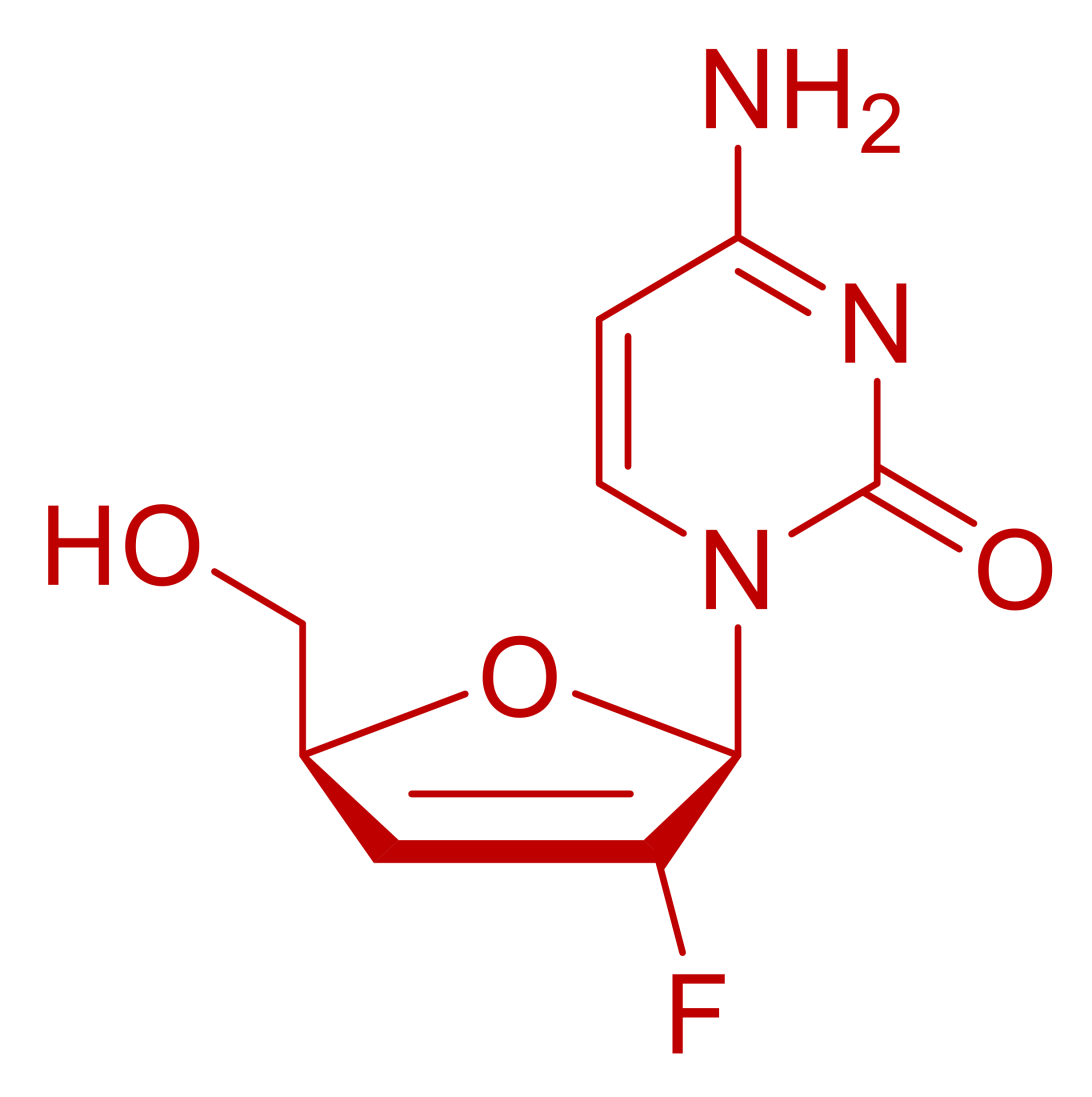	HIV	0.2 [102]	6.5 ^a^ [102]
	HBV	0.002 ^b^ [89]			HBV	>0.3 [102]	
**54** L-Fd4FC 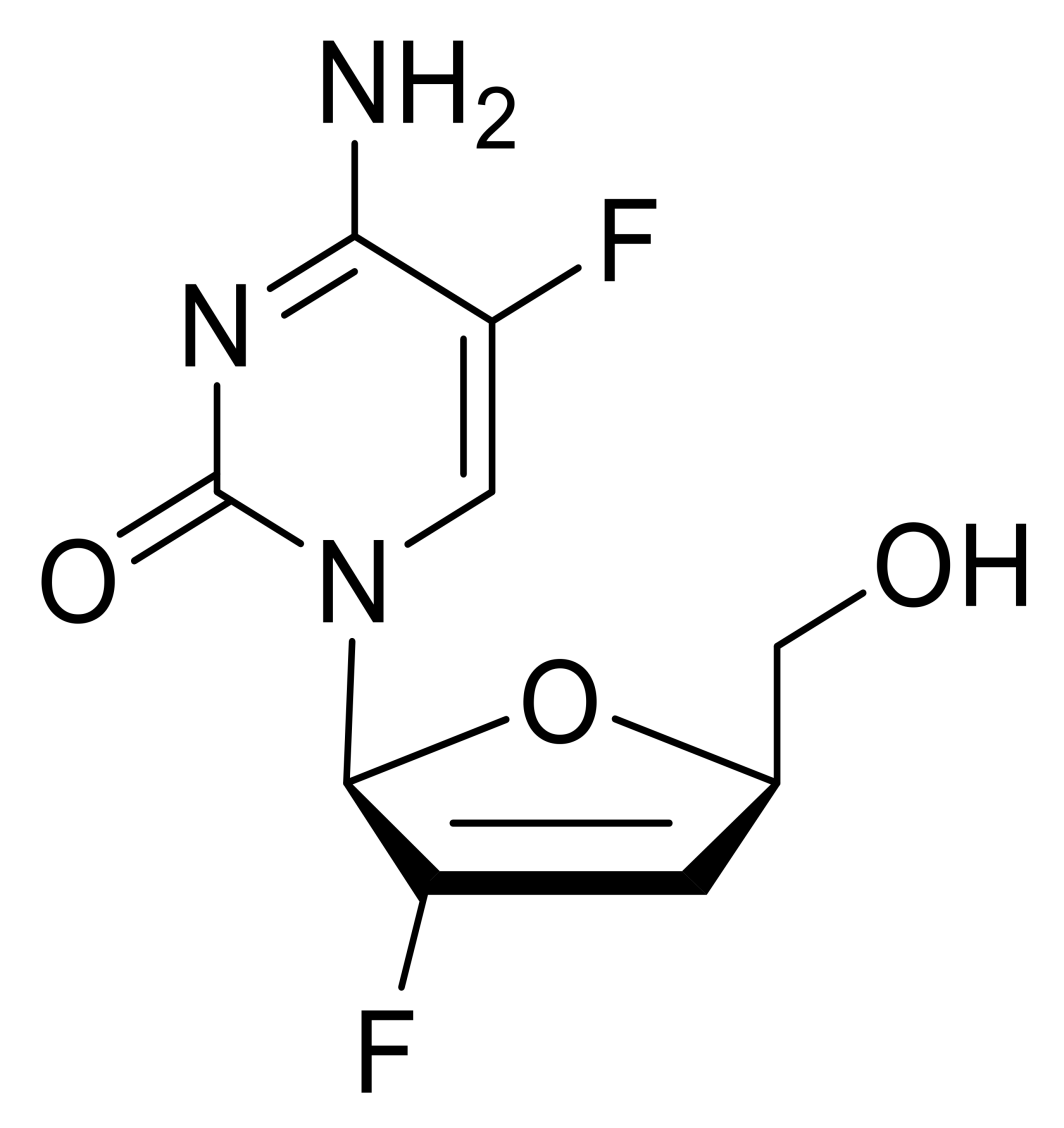	HIV	0.17 ^a^ [103]	>100 [103]	**55** D-Fd4FC 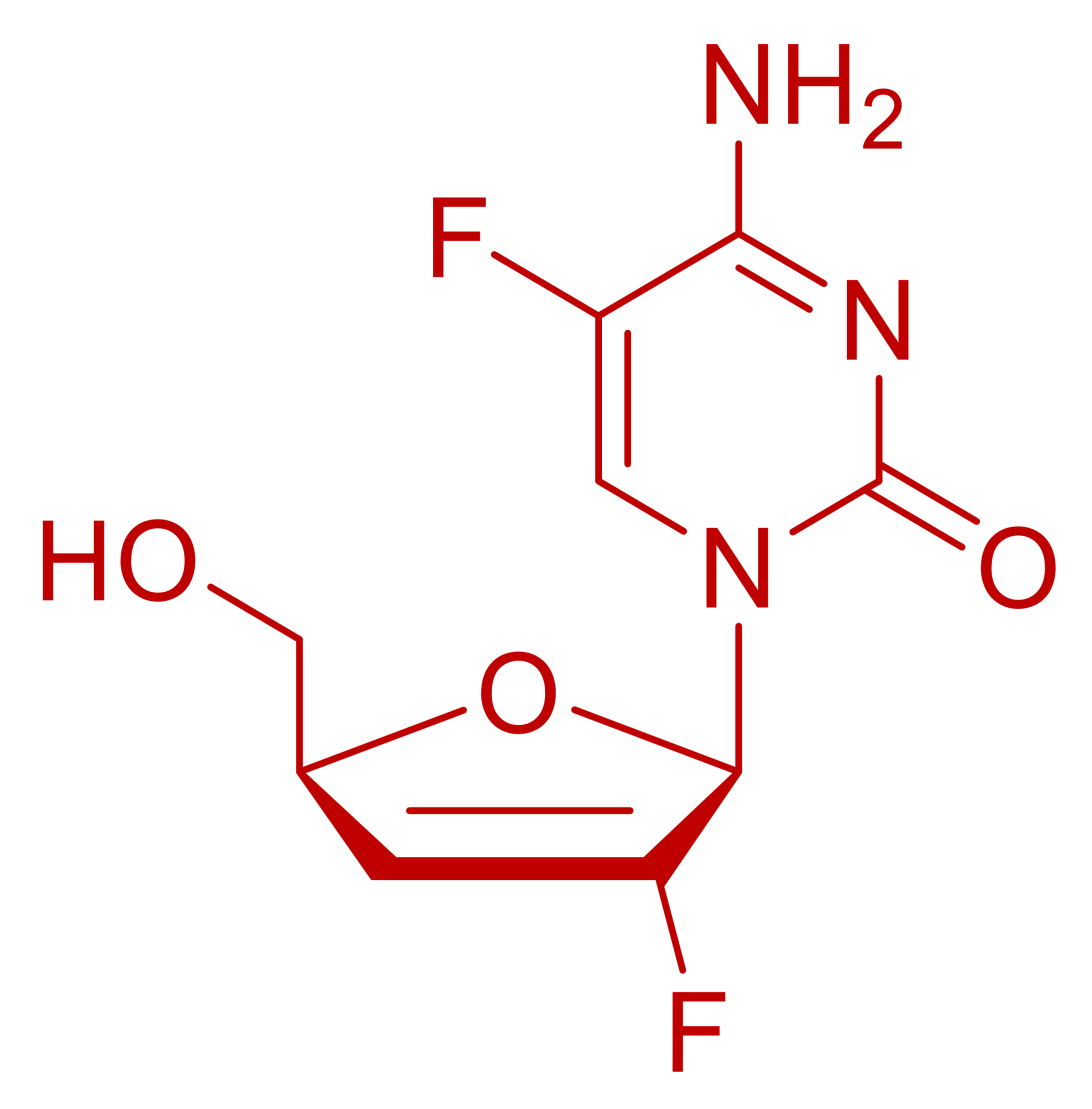	HIV	n/d	
	HBV	0.004 ^b^ [104]			HBV	0.05 [104]	>100 [105]
**56** L-Fd4A 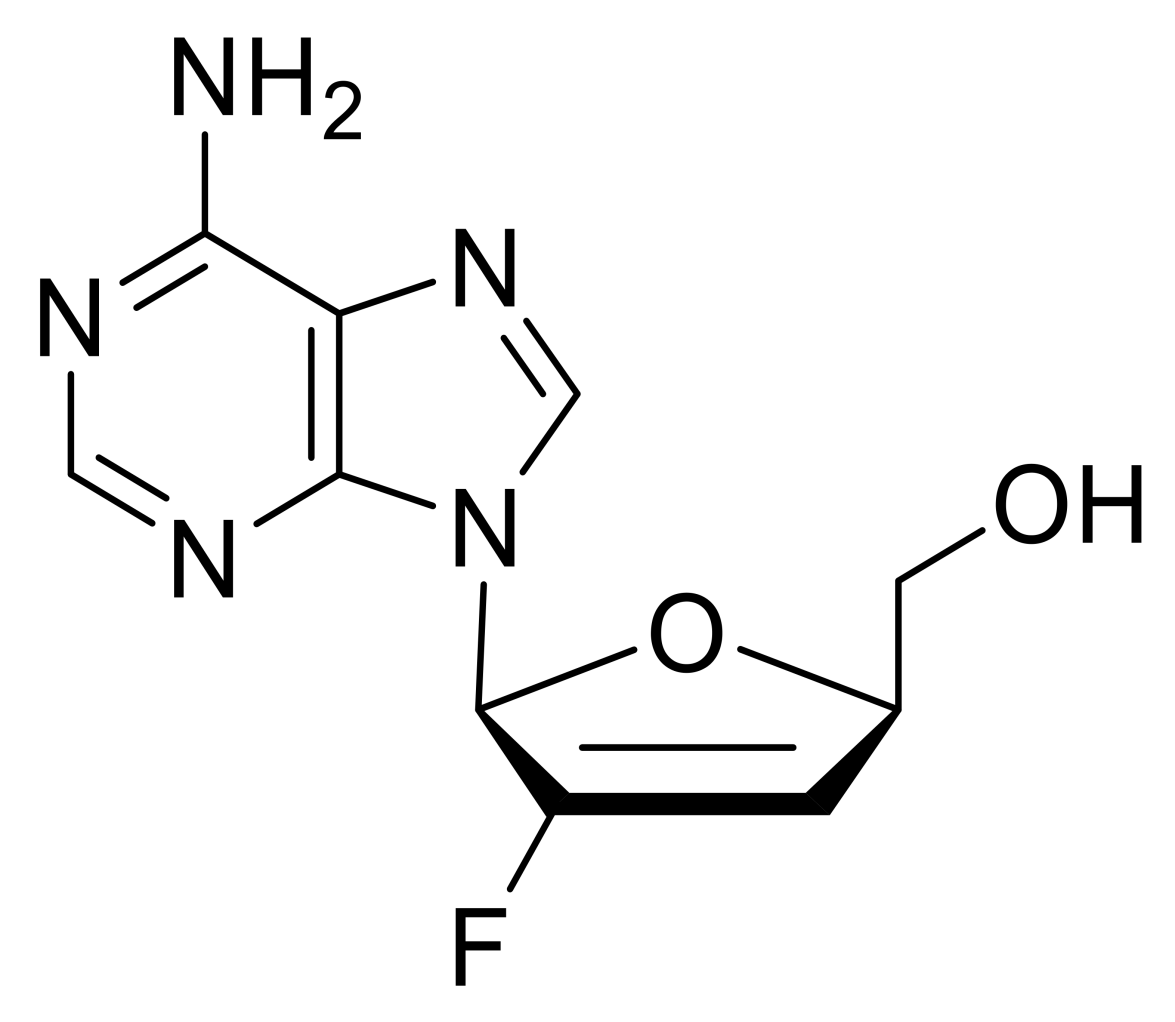	HIV	1.5 ^a^ [103]	>100 [103]	**57** D-Fd4A 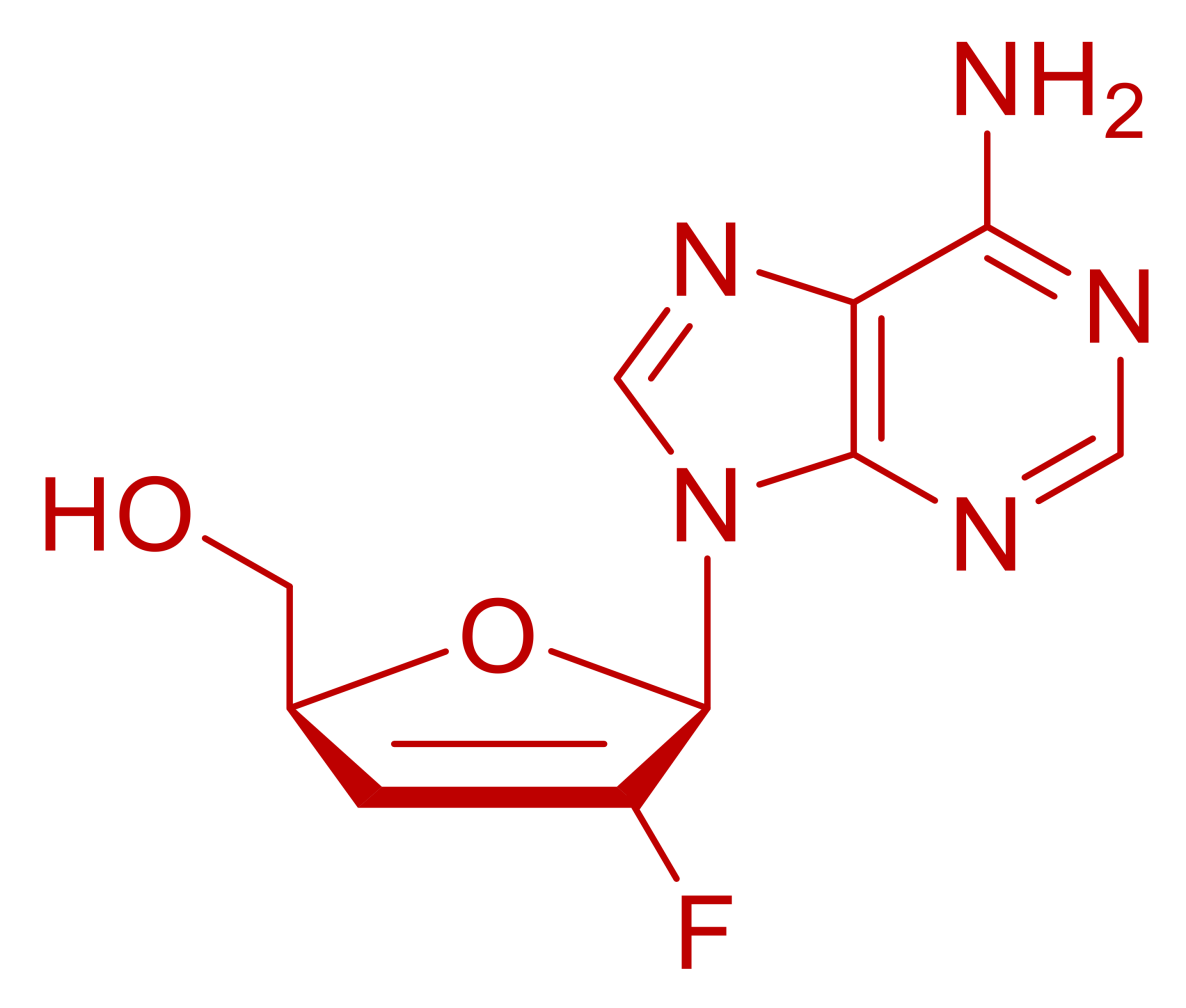	HIV	0.44 [86]	68.3 ^g^ [105]
	HBV	1.7 ^b^ [103]			HBV	n/d	
**58** 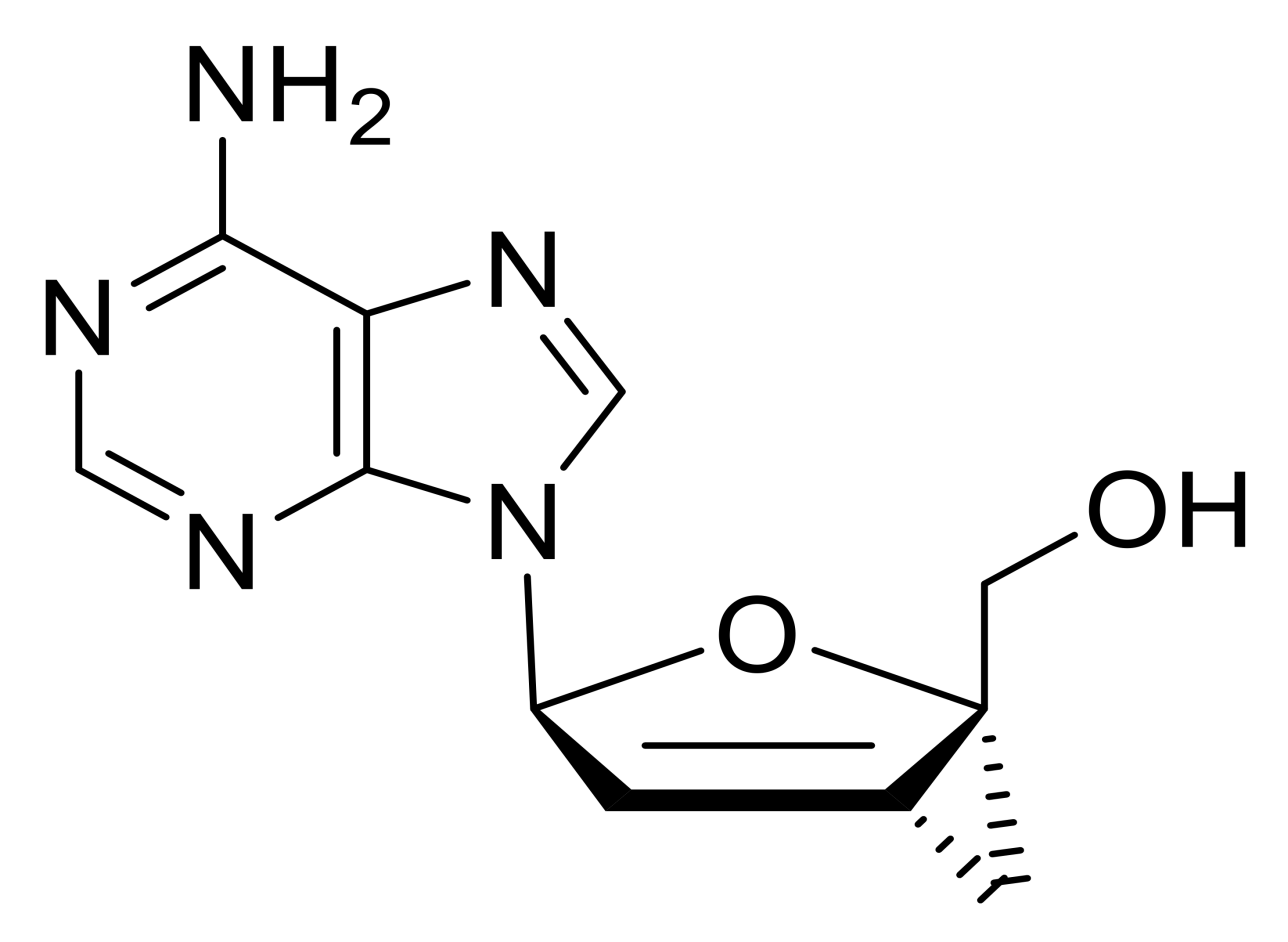	HIV-1	>400 ^d,^*	>400 ^d,^* [98]	**59** 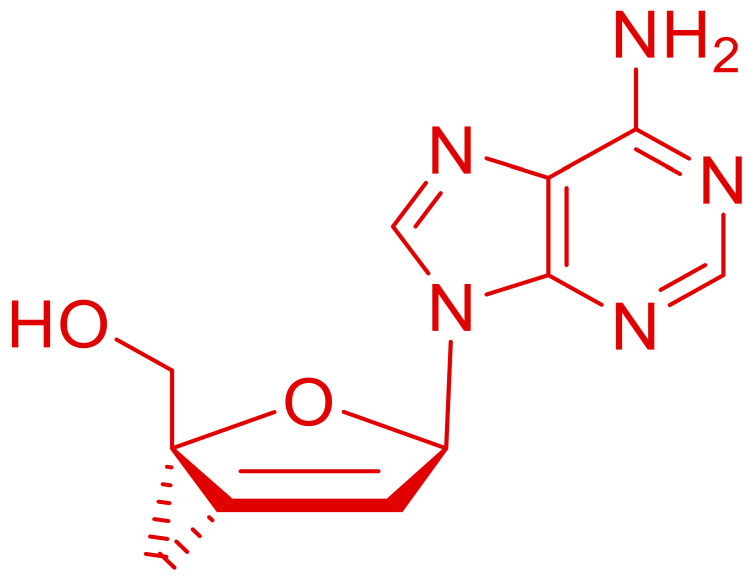	HIV-1	165^d*^	>400 ^d,^* [98]
	HIV-2	>400 ^d,^*			HIV-2	>400 ^d,^*	
**60** 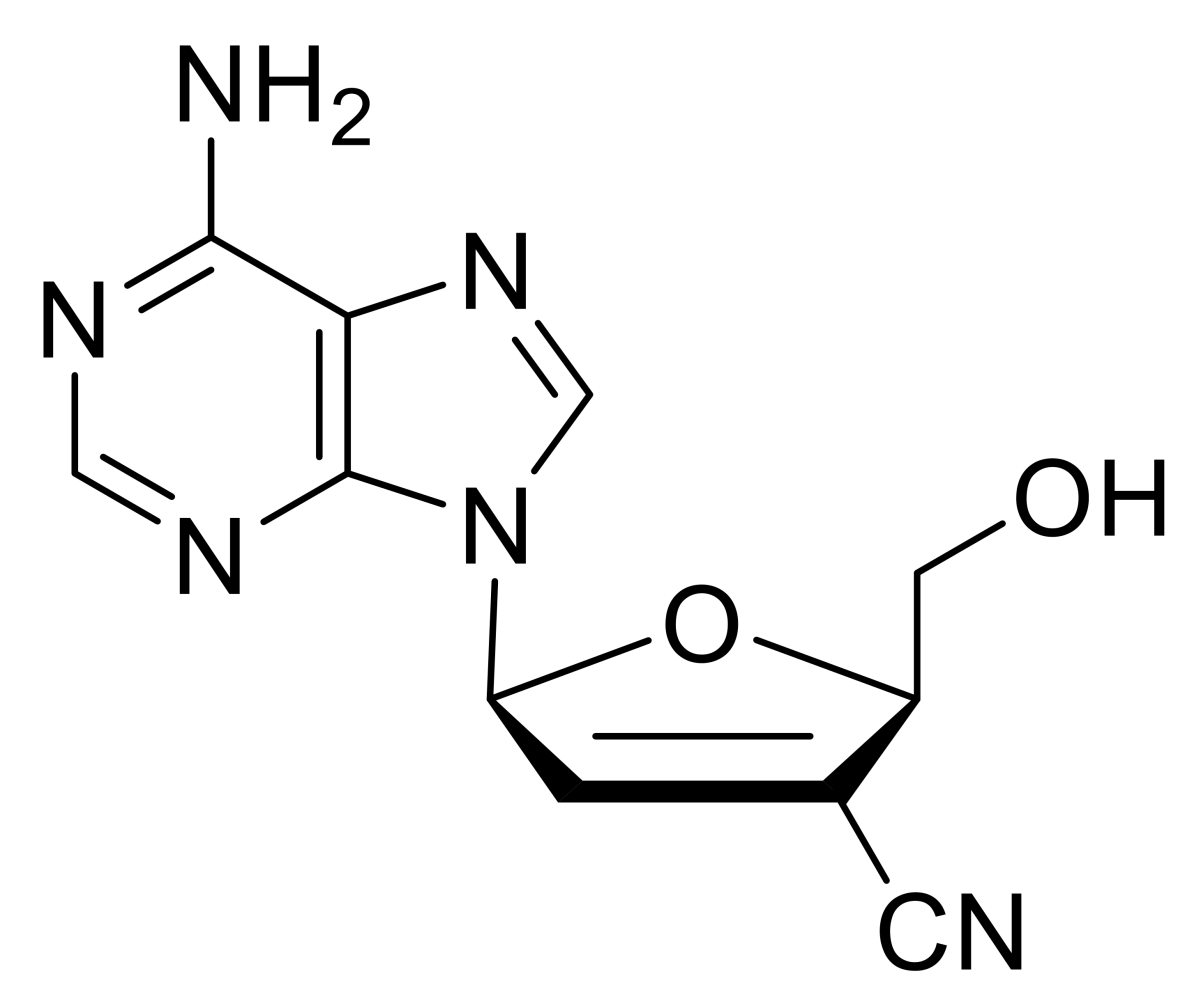	HIV	261 ^a^ [99]	>350 ^a^ [99]	-	-	-	-

^a^ EC_50_ on PBM cells. ^b^ EC_50_ on Hep-G2/2.2.15 cells. ^c^ EC_50_ on CEM cells. ^d^ EC_50_ on MT4 cells. ^e^ IC_50_; the tests were carried out on different lines of carcinoma cancer cells. ^f^ EC_50_ on H1 cells. ^g^ Cytotoxicity was estimated in CEM cells. ^h^ Human hepatoma cells 2.2.15; MT-2. ^i^ Cytotoxicity was estimated in Vero cells. * Recalculated data.

**Table 2:** Nucleoside phosphonate and phosphate prodrugs.

Nucleoside Analogue	Virus	IC_50_, μM	Cytotoxicity, IC_50_, μM
**61** Cidofovir (*(S)*-HPMPC) Vistide^®^ 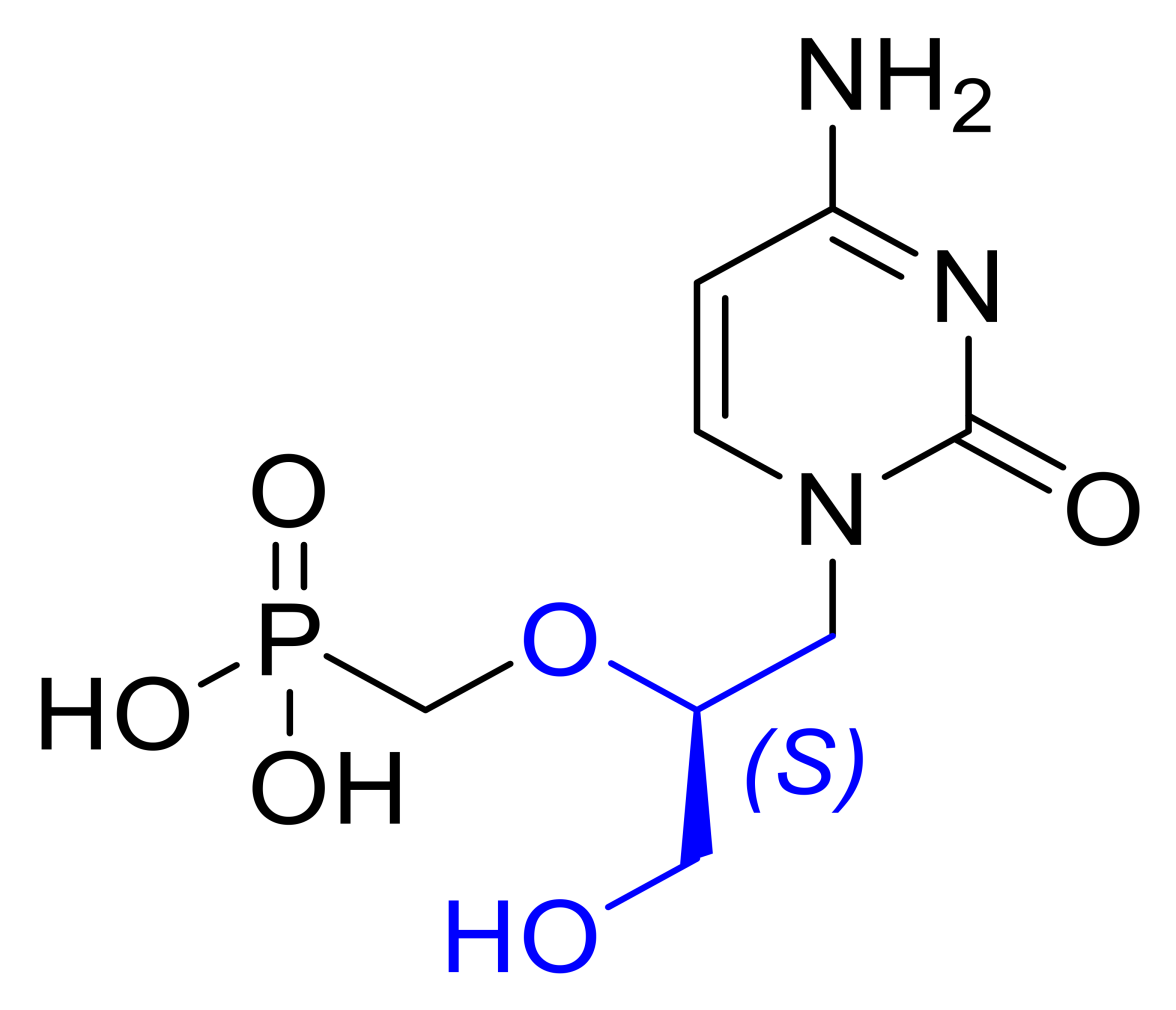	CPXV (Cowpox)	20–30 [115]	≥100 [115]
	HCMV	0.28 [115]	
**62** Adefovir (PMEA) 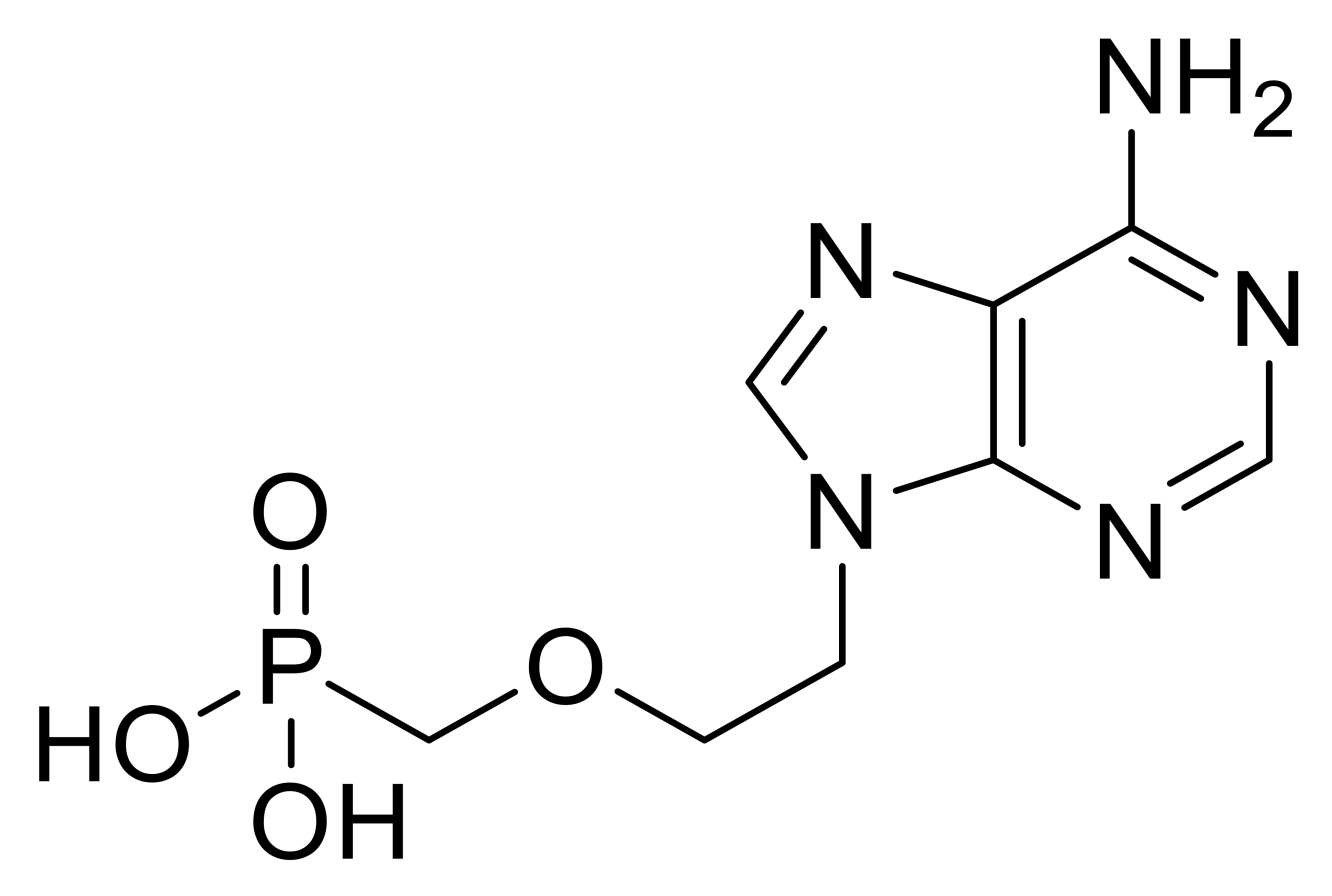	HIV	13.4 ± 4.2 [116]	225 ± 25 [116]
**63** Tenofovir (*(R)*-PMPA) 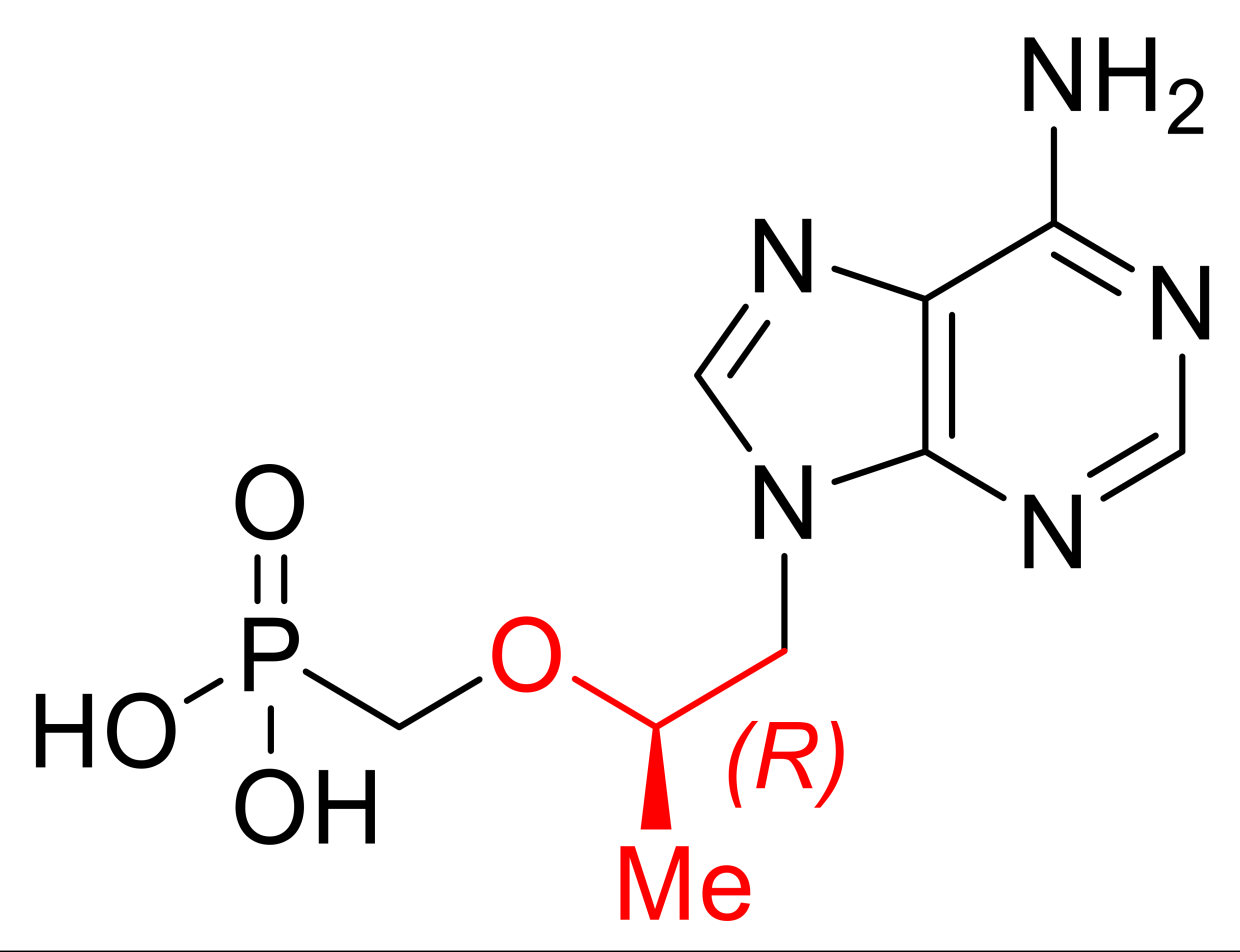	HBV	1.5 ± 0.05 [117]	
	DHBV	0.11 ± 0.02 [118]	>100 [119,120]
	HIV-1	0.5 [118]	
**64** Tenofovir Disoproxil Fumarate (TDF) Viread^®^ 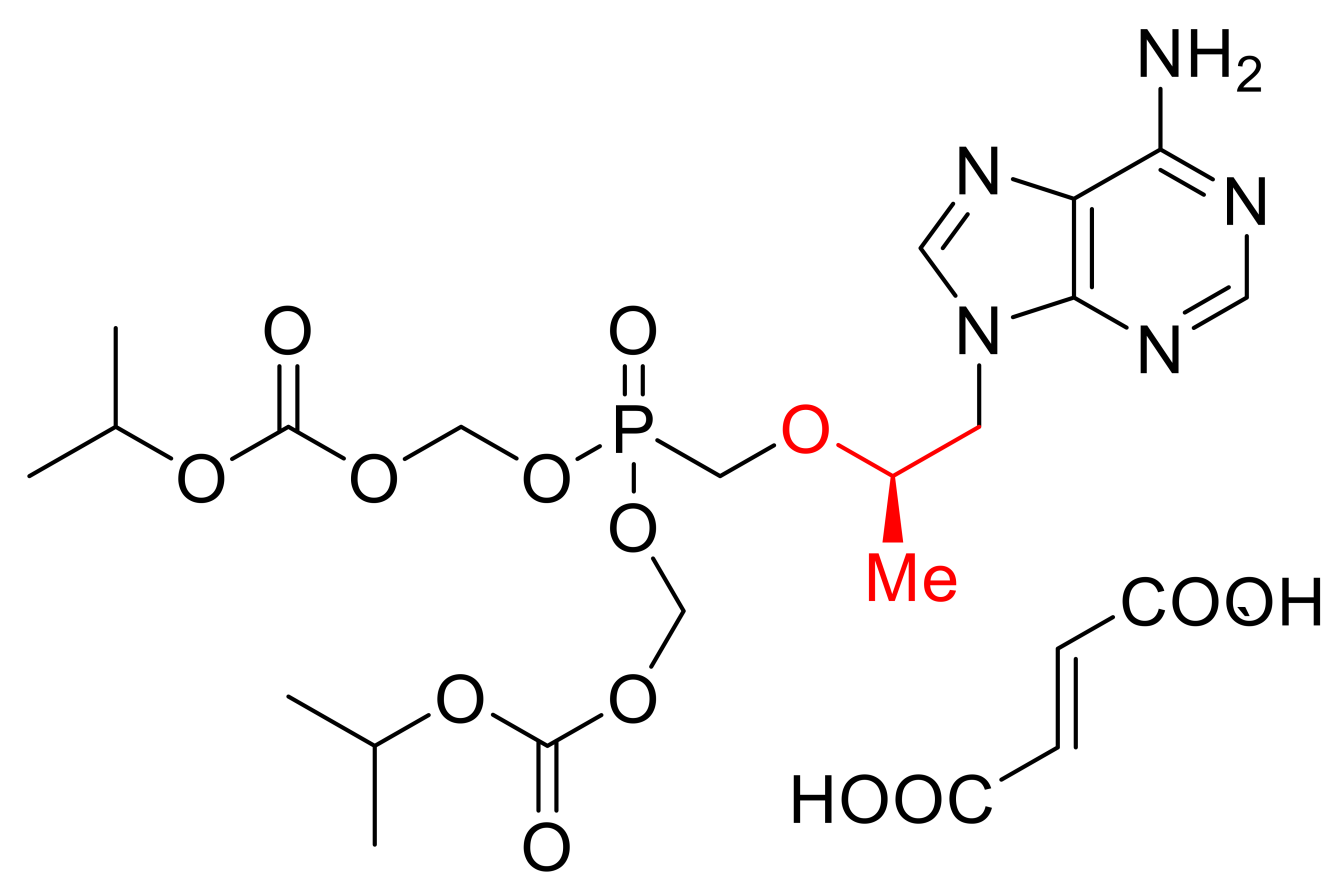	HIV-1	0.003 [118]	50 ^a^ [118]
**65** Tenofovir Alafenamide (TAF) 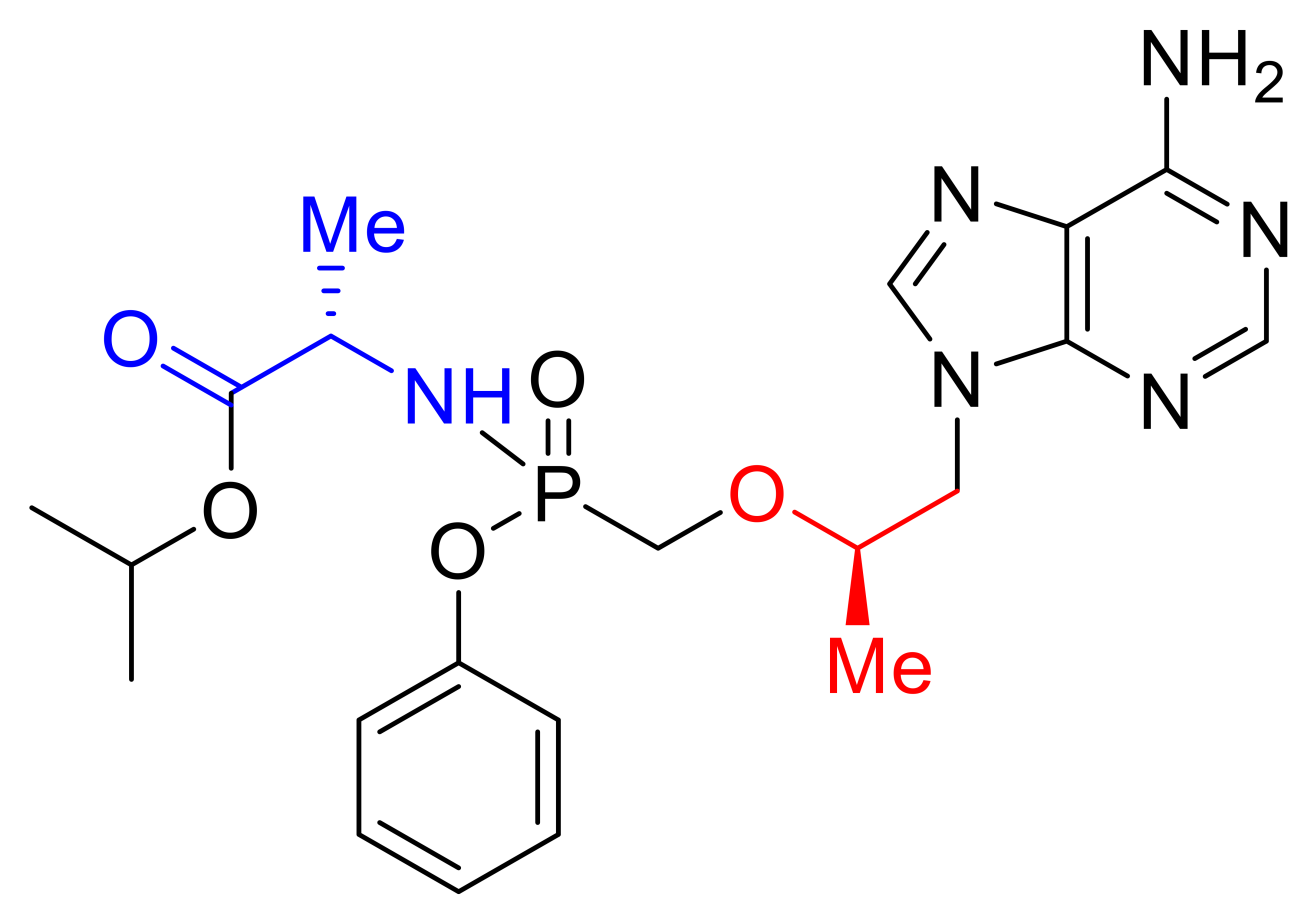	HIV-1	0.005 ± 0.002 ^b^ [119]	42 ^a^ [119]
**66***(S)*-HPMPA 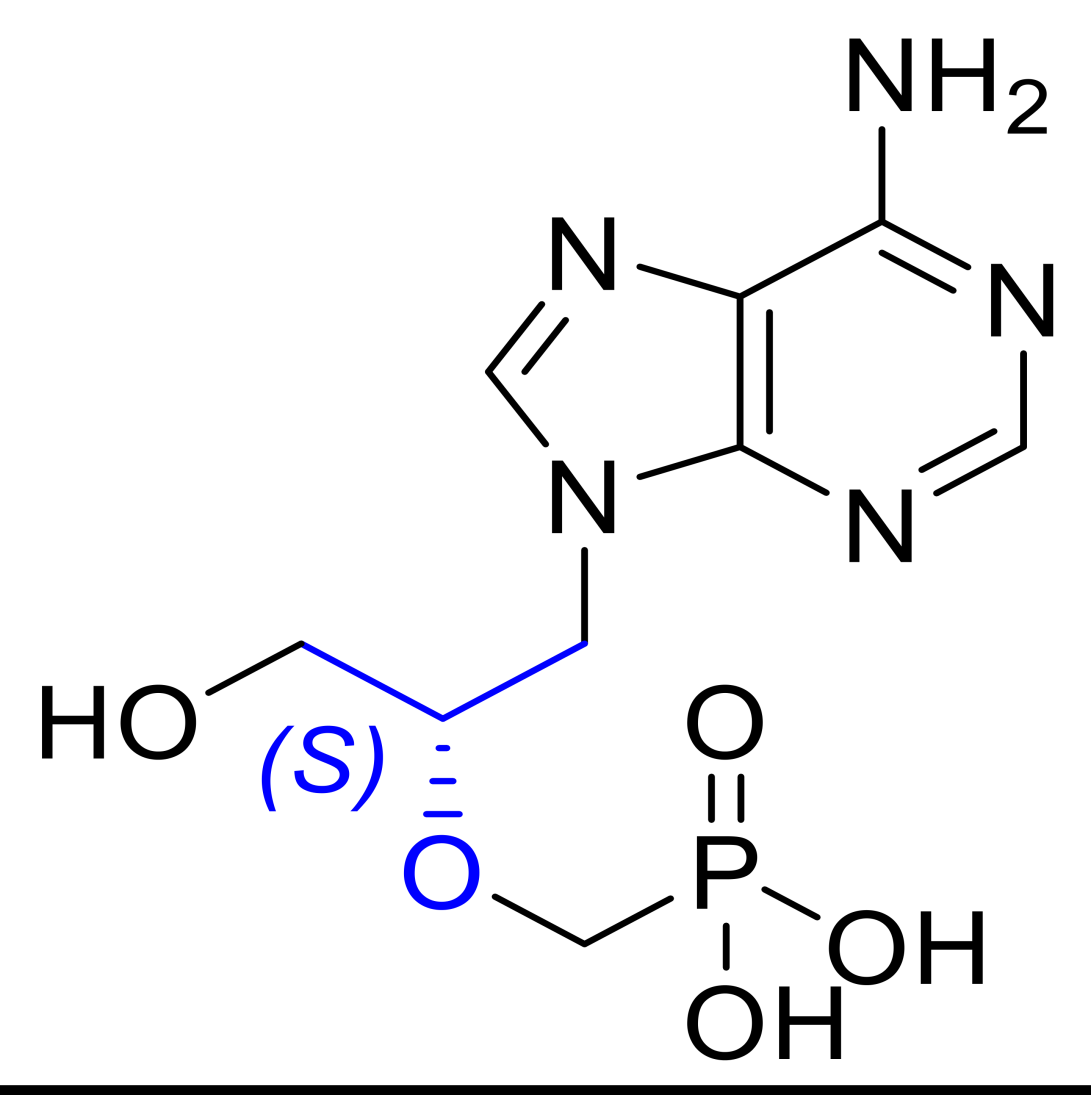	CPXV (Cowpox)	2–4 [115]	≥100 [115]
	HSV-1	35 [115]	
	HCMV	0.41 [115]	

**Table 3:** Comparison of chemical and enzymatic syntheses.

	Chemical Method			Enzymatic Method		
	Nucleoside Type					
	Ribo-	2′-Deoxyribo-	*ara-*	Ribo-	2′-Deoxyribo-	*ara*
Regioselectivity	High	Low-medium ^1^	Low-Medium ^1^	High	High	High
Yields	8–90% [142]	21–91% ^2^ [142]	7–91% ^2^ [142]	60–100% ^3^ [144,145]	20–100% ^3^ [144,145,146]	48–88% ^3^ [144]
Scalability	+	+	+	+	+	+
Protective groups	+	+	+	−	−	−
Number of steps	>1	>1	>1	1 ^4^	1 ^4^	1 ^4^
Cost-effectiveness	+	−	−	+	+	+
Environmental friendliness	−	−	−	+	+	+

^1^ Formation of racemic mixtures; ^2^ β-anomer, depending on protective groups; ^3^ β-anomer, depending on enzyme and concentration of reaction components, maximal yields in diluted solutions [146]; ^4^ *in situ*.

**Table 4:** Triggering biological activity through various chiral groups.

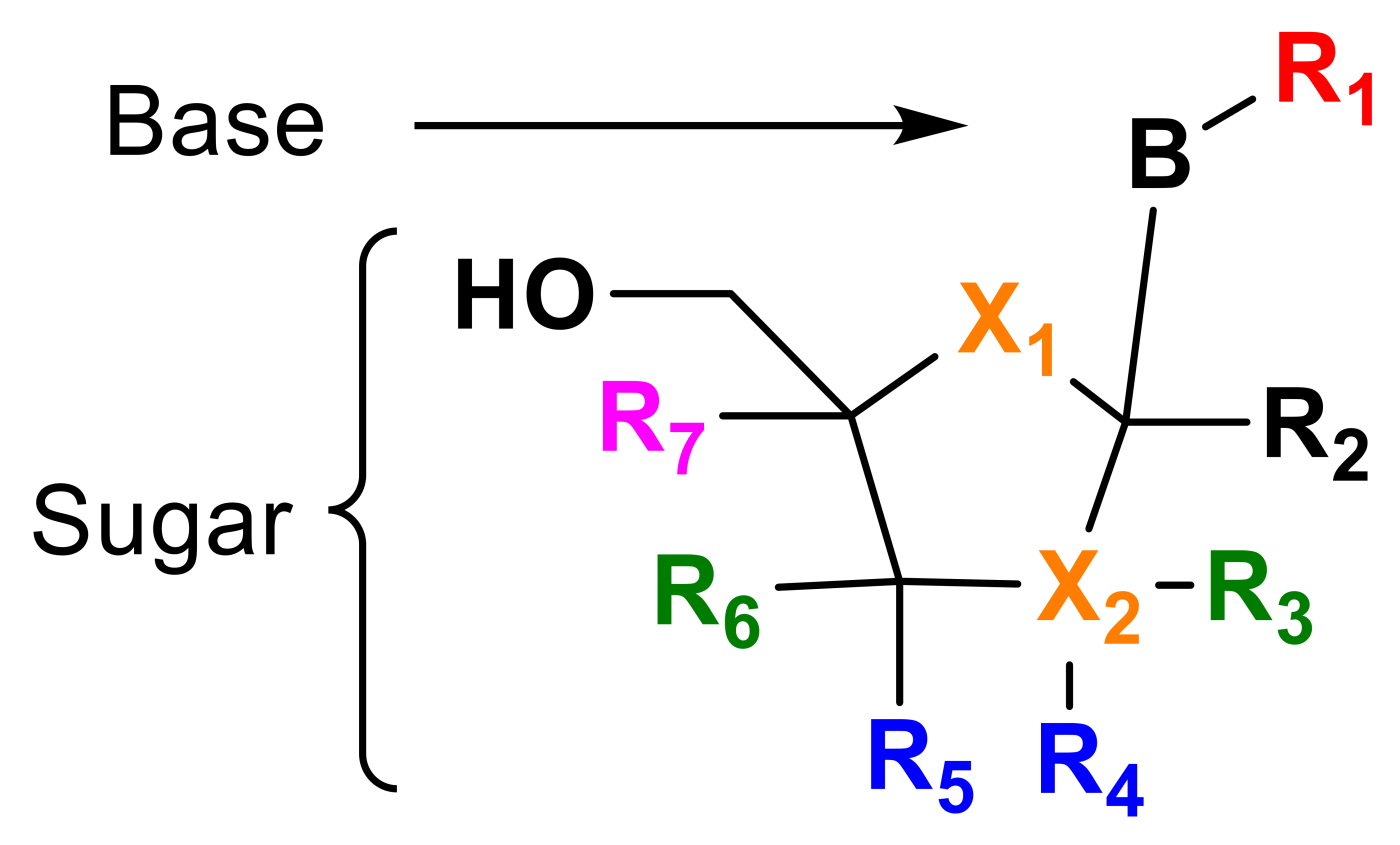												
Base	R1	Sugar	X1	X2	R2	R3	R4	R5	R6	R7	Biological Activity:	
											On	Off
Pyrimidine	-	D-pentose	O	C	H	OH	H	OH	H	H	Anticancer Antiviral	
Purine	-	D-pentose	O	C	H	OH	H	OH	H	H	Anticancer Antiviral	
Pyrimidine	-	D-pentose	O	C	H	F	F	OH	H	H	Anticancer (cytostatic)	
Pyrimidine	-	D-pentose	O	C	H	Me	F	OH	H	CH_2_Cl	Antiviral	
Purine	-	D-pentose	O	C	H	Me	OH	OH	H	H	Antiviral	
Purine	-	D-pentose	O	C	H	H	OH	OH	Me	H	Anticancer	
Purine	Cyclic alkane	D-pentose	O	C	H	H	OH	OH	Me	H	Anticancer	
Purine	Small polar group	D-pentose	O	C	H	H	OH	OH	Me	H	Anticancer	
Purine	*R*-PhEt	D-pentose	O	C	H	Me	OH	OH	H	H	Partial activation of ARs	
Purine	*R*-PhEt	D-pentose	O	C	Me	H	OH	OH	H	H	Partial activation of ARs	
Purine	Alkyl/hetaryl/Cl	Metanocarba-	CH	C	H	H	OH	OH	endo-CH_2_ with X_1_		ARs, DPRs, ORs, SRs	
Purine	Alkyl + Cl	Metanocarba (CH_2_OH subst.CH_2_N_3_/Hal)	CH	C	H	H	OH	OH			Antiviral	Cytotoxicity
Pyrimidine	-	D-pentose	O	S	H	H	H	H	H	H	Antiviral, cytotoxicity	
Pyrimidine	-	L-pentose	O	S	H	H	H	H	H	H	Antiviral	Cytotoxicity
Pyrimidine	-	D-pentose	O	O	H	H	H	H	H	H	Antiviral	Cytotoxicity
Pyrimidine	-	L-pentose	O	O	H	H	H	H	H	H	Antiviral	Cytotoxicity
Pyrimidine	I	D-pentose	O	O	H	H	H	H	H	H	Antiviral, Cytotoxicity	Cytotoxicity
Pyrimidine	I	L-pentose	O	O	H	H	H	H	H	H	High Antiviral	Cytotoxicity
Pyrimidine	-	L-pentose	O	C	H	H	H	OH	H	H	Antiviral	Cytotoxicity
Pyrimidine	-	D-pentose	O	C	H	H	Double bond		H	H	Antiviral, cytotoxicity	
Pyrimidine	-	L-pentose	O	C	H	H	Double bond		H	H	Antiviral	Cytotoxicity
Pyrimidine	-	D-pentose	O	C	H	H	Double bond		endo-CH_2_		Antiviral	Cytotoxicity
Purine	-	D-pentose	O	C	H	H	Double bond		endo-CH_2_			Antiviral, Cytotoxicity
Pyrimidine/Purine	-	L-pentose	O	C	H	H	Double bond		endo-CH_2_			Antiviral, Cytotoxicity
Purine/Pyrimidine	-	L-pentose	O	C	H	H	Double bond		CN	H		Antiviral, Cytotoxicity
Purine	-	L-pentose	O	C	H	F	Double bond		H	H	Antiviral,	Cytotoxicity
Purine	-	D-pentose	O	C	H	F	Double bond		H	H	Antiviral,	Cytotoxicity
Pyrimidine	-	D-pentose	O	C	H	F	H	OH	H	H	Antiviral, Cytotoxicity	
Pyrimidine	-	D-pentose	S	C	H	F	Double bond		H	H	Antiviral	Cytotoxicity
Pyrimidine	-	D-pentose	O	C	H	H	Double bond		F	H	Antiviral	Cytotoxicity
Pyrimidine	-	D-pentose	S	C	H	H	Double bond		F	H	Antiviral	Cytotoxicity
Purine	-	D-pentose	CH_2_	C	H	H	Double bond		F	H	Antiviral	Cytotoxicity
Pyrimidine	-	L-pentose	O	C	H	F	H	OH	H	H	Antiviral	Cytotoxicity
Pyrimidine	-	L-pentose	O	C	H	H	Double bond		F	H	Antiviral	Cytotoxicity
Pyrimidine	-	L-pentose	S	C	H	H	Double bond		F	H	Antiviral	Cytotoxicity
Pyrimidine	-	L-pentose	S	C	H	F	Double bond		H	H	Antiviral	Cytotoxicity
Pyrimidine/Purine	-	L-pentose	O	C	H	F	H	N_3_	H	H		Antiviral Cytotoxicity
Purine	Amino acid	D-pentose	O	C	H	H	H OH	OH	H	H	ARs, kinases	
Purine	Cl, NH_2_	Acyclic chiral	-	-	-	-	-	-	-	-	Antiviral, Cytotoxicity	

## Data Availability

The original contributions presented in this study are included in the article. Further inquiries can be directed to the corresponding author.

## Abbreviations

The following abbreviations are used in this manuscript:

AIDS	Acquired immunodeficiency syndrome
AR	Adenosine receptor
ATP	Adenosine 5′-triphosphate
AZT	2′,3′-Dideoxy-3′-azidothymidine (Zidovudine)
BAR	*N*^6^-Benzyladenosine
cAMP	Cyclic adenosine 5′-monophosphate
CPXV	Cowpox virus
CTP	Cytidine 5′-triphosphate
CTSA	Cathepsin A
ddC	2′,3′-Dideoxycytidine (Zalcitabine)
ddI	2′,3′-Dideoxyinosine (Didanosine)
D-FMAU	1-(β-*D*-2′-Fluoro-2′-deoxyarabinofuranosyl)-5-methyluracil
DNA	Deoxyribonucleic acid
EBV	Epstein–Barr virus
EVA71	Enterovirus A71
FDA	Food and Drug Administration
GSH	Glutathione
HBV	Herpes B virus
HCMV	Human cytomegalovirus
HCV	Hepatitis C virus
HIV	Human immunodeficiency virus
HSV	Herpes simplex virus
HSV	Herpes virus
IMP	Inosine 5′-monophosphate
IPR	*N*^6^-Isopentenyladenosine
KINR	Kinetin riboside, *N*^6^-furfuryladenosine
L-FMAU	1-(β-*L*-2′-Fluoro-2′-deoxyarabinofuranosyl)-5-methyluracil
MBH	Morita–Baylis–Hillman reaction
NA	Nucleic acid
NAD	Nicotinamide adenine dinucleotide
NP	Nucleoside phosphorylase
PROTIDE	Prodrug + nucleotide technology
RR	Ribonucleotidediphosphate reductase
RSV	Respiratory syncytial virus
RT	Reverse transcriptase
SAH	S-adenosylhomocysteine
SAR	Structure-activity relationship
TAF	Tenofovir Alafenamide
TDF	Tenofovir Disoproxil Fumarate
Tdp1	Tyrosyl-DNA phosphodiesterase-1
tRNA	Transfer ribonucleic acid
VZV	Varicella zoster virus
WHV	Woodchuck hepatitis virus
α-Ara-1-P	1-α-D-Arabinofuranosylphosphate
α-Rib-1-P	1-α-D-Ribofuranosylphosphate

## References

[1] Smith H.J., Williams H. (1998). Smith and Williams’ Introduction to the Principles of Drug Design and Action.

[2] Clayden J., Greeves N., Warren S. (2012). Organic Chemistry.

[3] Yu X., Yao Z.-P. (2017). Chiral Recognition and Determination of Enantiomeric Excess by Mass Spectrometry: A Review. Anal. Chim. Acta.

[4] Mathews C.K., Van Holde K.E. (1996). Biochemistry.

[5] Wakabayashi M., Wakabayashi H., Nörenberg S., Kubota K., Engel K. (2015). Comparison of odour thresholds and odor qualities of the enantiomers of 4-mercapto-2-alkanones and 4-acetylthio-2-alkanones. Flavour. Fragr. J..

[6] Zenchenko A.A., Drenichev M.S., Il’icheva I.A., Mikhailov S.N. (2021). Antiviral and Antimicrobial Nucleoside Derivatives: Structural Features and Mechanisms of Action. Mol. Biol..

[7] Murakami H., Sakai K., Hirayama N., Tamura R. (2006). From Racemates to Single Enantiomers—Chiral Synthetic Drugs over the Last 20 Years. Novel Optical Resolution Technologies.

[8] Food and Drug Administration (1992). FDA’S Policy Statement for the Development of New Stereoisomeric Drugs. Chirality.

[9] Woolf A.D., Woolf A., Wexler P., Bateman N., Brent J., Deng J.-F., Smolinske S., Yang C.-C. (2022). Thalidomide tragedy, 1950s. History of Modern Clinical Toxicology.

[10] Nguyen L.A., He H., Pham-Huy C. (2006). Chiral Drugs: An Overview. Int. J. Biomed. Sci..

[11] Cano-Soldado P., Pastor-Anglada M. (2012). Transporters That Translocate Nucleosides and Structural Similar Drugs: Structural Requirements for Substrate Recognition. Med. Res. Rev..

[12] Jordheim L.P., Durantel D., Zoulim F., Dumontet C. (2013). Advances in the Development of Nucleoside and Nucleotide Analogues for Cancer and Viral Diseases. Nat. Rev. Drug Discov..

[13] Meier C. (2017). Nucleoside diphosphate and triphosphate prodrugs—An unsolvable task?. Antivir. Chem. Chemother..

[14] Yates M.K., Seley-Radtke K.L. (2019). The Evolution of Antiviral Nucleoside Analogues: A Review for Chemists and Non-Chemists. Part II: Complex Modifications to the Nucleoside Scaffold. Antivir. Res..

[15] Bergmann W., Feeney R.J. (1951). Contributions to the study of marine products. XXXII. The nucleosides of sponges. I. J. Org. Chem..

[16] Plunkett W., Chubb S., Alexander L., Montgomery J.A. (1980). Comparison of the toxicity and metabolism of 9-beta-D-arabinofuranosyl-2-fluoroadenine and 9-beta-D-arabinofuranosyladenine in human lymphoblastoid cells. Cancer Res..

[17] de Sousa Cavalcante L., Monteiro G. (2014). Gemcitabine: Metabolism and molecular mechanisms of action, sensitivity and chemoresistance in pancreatic cancer. Eur. J. Pharmacol..

[18] Suzuki M., Okuda T., Shiraki K. (2006). Synergistic Antiviral Activity of Acyclovir and Vidarabine against Herpes Simplex Virus Types 1 and 2 and Varicella-Zoster Virus. Antivir. Res..

[19] Yathindra N., Sundaralingam M. (1979). Conformational analysis of arabinonucleosides and nucleotides A comparison with the ribonucleosides and nucleotides. Biochim. Biophys. Acta (BBA) -Nucleic Acids Protein Synth..

[20] Bosc J.J., Latxague L., Léger J.M., Balzarini J., Forfar I., Jarry C., Guillon J. (2010). Synthesis and in vitro cytostatic activity of new beta-D-arabino furan[1′,2′:4,5]oxazolo- and arabino-pyrimidinone derivatives. Eur. J. Med. Chem..

[21] Liu P., Sharon A., Chu C.K. (2008). Fluorinated Nucleosides: Synthesis and Biological Implication. J. Fluor. Chem..

[22] Das G., Harikrishna S., Gore K.R. (2022). Influence of Sugar Modifications on the Nucleoside Conformation and Oligonucleotide Stability: A Critical Review. Chem. Rec..

[23] Mikami A., Erande N., Matsuda S., Kel’in A., Woods L.B., Chickering T., Pallan P.S., Schlegel M.K., Zlatev I., Egli M. (2020). Synthesis, chirality-dependent conformational and biological properties of siRNAs containing 5′-(R)- and 5′-(*S*)-*C*-methyl-guanosine. Nucleic Acids Res..

[24] Eyer L., Fojtíková M., Nencka R., Rudolf I., Hubálek Z., Ruzek D. (2019). Viral RNA-Dependent RNA Polymerase Inhibitor 7-Deaza-2′-*C*-Methyladenosine Prevents Death in a Mouse Model of West Nile Virus Infection. Antimicrob. Agents Chemother..

[25] Clark J.L., Hollecker L., Mason J.C., Stuyver L.J., Tharnish P.M., Lostia S., McBrayer T.R., Schinazi R.F., Watanabe K.A., Otto M.J. (2005). Design, Synthesis, and Antiviral Activity of 2′-Deoxy-2′-Fluoro-2′-*C*-Methylcytidine, a Potent Inhibitor of Hepatitis C Virus Replication. J. Med. Chem..

[26] Suresh R.R., Abuduani T., Kasthuri M., Chen Z., Tber Z., Loubidi M., Zhang H., Zhou L., Zhou S., Li C. (2026). Prodrug strategies in developing antiviral nucleoside analogs. RSC Med. Chem..

[27] Mikhailov S.N., Beigelman L.N., Gurskaya G.V., Padyukova N.S., Yakovlev G.I., Karpeisky M.Y. (1983). Synthesis and Properties of 3′-*C*-Methylnucleosides and Their Phosphoric Esters. Carbohydr. Res..

[28] Franchetti P., Cappellacci L., Pasqualini M., Petrelli R., Vita P., Jayaram H.N., Horvath Z., Szekeres T., Grifantini M. (2005). Antitumor activity of *C*-Methyl-b-D-ribofuranosyladenine Nucleoside Ribonucleotide Reductase Inhibitors. J. Med. Chem..

[29] Cappellacci L., Franchetti P., Vita P., Petrelli R., Lavecchia A., Jayaram H.N., Saiko P., Graser G., Szekeres T., Grifantini M. (2008). Ribose-modified purine nucleosides as ribonucleotide reductase inhibitors. Synthesis, antitumor activity, and molecular modeling of *N*6-substituted 3’-*C*-methyladenosine derivatives. J. Med. Chem..

[30] Matsuda A., Sasaki T. (2004). Antitumor activity of sugar-modified cytosine nucleosides. Cancer Sci..

[31] Cappellacci L., Petrelli R., Franchetti P., Vita P., Kusumanchi P., Kumar M., Jayaram H.N., Zhou B., Yen Y., Grifantini M. (2011). Synthesis and biological activity of novel N6-substituted and 2, *N*^6^-disubstituted adenine ribo-and 3′-*C*-methyl-ribonucleosides as antitumor agents. Eur. J. Med. Chem..

[32] Martin J.C., Hitchcock M.J.M., De Clercq E., Prusoff W.H. (2010). Early Nucleoside Reverse Transcriptase Inhibitors for the Treatment of HIV: A Brief History of Stavudine (D4T) and Its Comparison with Other Dideoxynucleosides. Antivir. Res..

[33] Daly J.W. (1982). Adenosine receptors: Targets for future drugs. J. Med. Chem..

[34] Jacobson K.A., Müller C.E. (2016). Medicinal Chemistry of Adenosine, P2Y and P2X Receptors. Neuropharmacology.

[35] Bahreyni A., Saeedi N., Al-Asady A.M., Soleimani A., Ghorbani E., Khazaei M., Alaei M., Hanaei R., Ryzhikov M., Avan A. (2024). Therapeutic potency of A1 adenosine receptor antagonists in the treatment of cardiovascular diseases, current status and perspectives. Mol. Biol. Rep..

[36] Feoktistov I., Goldstein A.E., Biaggioni I. (1999). Role of p38 mitogen-activated protein kinase and extracellular signal-regulated protein kinase kinase in adenosine A2B receptor-mediated interleukin-8 production in human mast cells. Mol. Pharmacol..

[37] Cappellacci L., Barboni G., Palmieri M., Pasqualini M., Grifantini M., Costa B., Martini C., Franchetti P. (2002). Ribose-Modified Nucleosides as Ligands for Adenosine Receptors: Synthesis, Conformational Analysis, and Biological Evaluation of 1’-*C*-Methyl Adenosine Analogues. J. Med. Chem..

[38] Ballesteros-Yáñez I., Castillo C.A., Merighi S., Gessi S. (2018). The Role of Adenosine Receptors in Psychostimulant Addiction. Front. Pharmacol..

[39] Gessi S., Varani K., Merighi S., Ongini E., Borea P.A. (2000). A_2A_ Adenosine Receptors in Human Peripheral Blood Cells. Br. J. Pharmacol..

[40] Cekic C., Sag D., Li Y., Theodorescu D., Strieter R.M., Linden J. (2012). Adenosine A2B Receptor Blockade Slows Growth of Bladder and Breast Tumors. J. Immunol..

[41] Gao Z.-G., Jacobson K.A. (2019). A2B Adenosine Receptor and Cancer. Int. J. Mol. Sci..

[42] Gao Z.G., Blaustein J.B., Gross A.S., Melman N., Jacobson K.A. (2003). N6-Substituted adenosine derivatives: Selectivity, efficacy, and species differences at A3 adenosine receptors. Biochem. Pharmacol..

[43] Baraldi P.G., Tabrizi M.A., Gessi S., Borea P.A. (2008). Adenosine receptor antagonists: Translating medicinal chemistry and pharmacology into clinical utility. Chem. Rev..

[44] Li B., Rosenbaum P.S., Jennings N.M., Maxwell K.M., Roth S. (1999). Differing roles of adenosine receptor subtypes in retinal ischemia-reperfusion injury in the rat. Exp. Eye Res..

[45] Polska E., Ehrlich P., Luksch A., Fuchsjäger-Mayrl G., Schmetterer L. (2003). Effects of adenosine on intraocular pressure, optic nerve head blood flow, and choroidal blood flow in healthy humans. Investig. Ophthalmol. Vis. Sci..

[46] Zhong Y., Yang Z., Huang W.C., Luo X. (2013). Adenosine, adenosine receptors and glaucoma: An updated overview. Biochim. Biophys. Acta Gen. Subj..

[47] Brown R.A., Spina D., Page C.P. (2008). Adenosine receptors and asthma. Br. J. Pharmacol..

[48] Blad C.C., Von Frijtag Drabbe Künzel J.K., de Vries H., Mulder-Krieger T., Bar-Yehuda S., Fishman P., Ijzerman A.P. (2011). Putative Role of the Adenosine A_3_ Receptor in the Antiproliferative Action of N^6^-(2-Isopentenyl)Adenosine. Purinergic Signal..

[49] Fishman P., Bar-Yehuda S., Synowitz M., Powell J.D., Klotz K.N., Gessi S., Borea P.A., Wilson C.N., Mustafa S.J. (2009). Adenosine Receptors and Cancer. Adenosine Receptors in Health and Disease.

[50] Ashton T.D., Aumann K.M., Baker S.P., Schiesser C.H., Scammells P.J. (2007). Structure–activity relationships of adenosine with heterocyclic *N*6-substituents. Bioorg. Med. Chem. Lett..

[51] Jacobson K.A., Kim H.O., Siddiqi S.M., Olah M.E., Stiles G.L., von Lubitz D.K. (1995). A3-adenosine receptors: Design of selective ligands and therapeutic prospects. Drugs Future.

[52] Peterman C., Sanoski C.A. (2005). Tecadenoson. Cardiol. Rev..

[53] Elzein E., Zablocki J. (2008). A1 adenosine receptor agonists and their potential therapeutic applications. Expert Opin. Investig. Drugs.

[54] Cheung J.W., Lerman B.B. (2003). CVT-510: A selective A1 adenosine receptor agonist. Cardiovasc. Drug Rev..

[55] Wilson C., Mustafa S. (2009). Adenosine Receptors in Health and Disease. Handbook of Experimental Pharmacology.

[56] Drenichev M.S., Borokh V.N. (2024). Purine nucleosides and analogues bearing chiral substituents: Medicinal chemistry and therapeutic perspective. Curr. Med. Chem..

[57] Van Galen P.J.M., van Bergen A.H., Gallo-Rodriguez C., Melman N., Olah M.E., IJzerman A.P., Stiles G.L., Jacobson K.A. (1994). A binding site model and structure-activity relationships for the rat A3 adenosine receptor. Mol. Pharmacol..

[58] Cappellacci L., Franchetti P., Pasqualini M., Petrelli R., Vita P., Lavecchia A., Novellino E., Costa B., Martini C., Klotz K.-N. (2005). Synthesis, biological evaluation, and molecular modeling of ribose-modified adenosine analogues as adenosine receptor agonists. J. Med. Chem..

[59] Man S., Lu Y., Yin L., Cheng X., Ma L. (2021). Potential and promising anticancer drugs from adenosine and its analogs. Drug Discov. Today.

[60] Jacobson K.A., Salmaso V., Suresh R.R., Tosh D.K. (2021). Expanding the repertoire of methanocarba nucleosides from purinergic signaling to diverse targets. RSC Med. Chem..

[61] Tosh D.K., Ciancetta A., Mannes P., Warnick E., Janowsky A., Eshleman A.J., Gizewski E., Brust T.F., Bohn L.M., Auchampach J.A. (2018). Repurposing of a nucleoside scaffold from adenosine receptor agonists to opioid receptor antagonists. ACS Omega.

[62] Tosh D.K., Salmaso V., Campbell R.G., Rao H., Bitant A., Pottie E., Stove C.P., Liu N., Gavrilova O., Gao Z.G. (2022). A3 adenosine receptor agonists containing dopamine moieties for enhanced interspecies affinity. Eur. J. Med. Chem..

[63] Andrei G., Topalis D., Fiten P., McGuigan C., Balzarini J., Opdenakker G., Snoeck R. (2012). In Vitro-Selected Drug-Resistant Varicella-Zoster Virus Mutants in the Thymidine Kinase and DNA Polymerase Genes Yield Novel Phenotype-Genotype Associations and Highlight Differences between Antiherpesvirus Drugs. J. Virol..

[64] Strasfeld L., Chou S. (2010). Antiviral drug resistance: Mechanisms and clinical implications. Infect. Dis. Clin. N. Am..

[65] Hatse S., De Clercq E., Balzarini J. (1999). Role of antimetabolites of purine and pyrimidine nucleotide metabolism in tumor cell differentiation. Biochem. Pharmacol..

[66] Leal J.F., Ferrer I., Blanco-Aparicio C., Hernandez-Losa J., Cajal S.R.Y., Carnero A., Lleonart M.E. (2008). S-adenosylhomocysteine hydrolase downregulation contributes to tumorigenesis. Carcinogenesis.

[67] De Clercq E. (1987). S-Adenosylhomocysteine hydrolase inhibitors as broad-spectrum antiviral agents. Biochem. Pharmacol..

[68] Wei H., Zhang R., Wang C., Zheng H., Li A., Chou K.C., Wei D.Q. (2007). Molecular insights of SAH enzyme catalysis and implication for inhibitor design. J. Theor. Biol..

[69] Yuan C.S., Saso Y., Lazarides E., Borchardt R.T., Robins M.J. (1999). Recent advances in S-adenosyl-L-homocysteine hydrolase inhibitors and their potential clinical applications. Expert Opin. Ther. Pat..

[70] Wang J.F., Yang X.D., Zhang L.R., Yang Z.J., Zhang L.H. (2004). Synthesis and biological activities of 5′-ethylenic and acetylenic modified L-nucleosides and isonucleosides. Tetrahedron.

[71] Ramasamy K.S., Tam R.C., Bard J., Averett D.R. (2000). Monocyclic L-nucleosides with type 1 cytokine-inducing activity. J. Med. Chem..

[72] Eichhorst B.F., Busch R., Hopfinger G., Pasold R., Hensel M., Steinbrecher C., Siehl S., Jäger U., Bergmann M., Stilgenbauer S. (2006). Fludarabine plus cyclophosphamide versus fludarabine alone in first-line therapy of younger patients with chronic lymphocytic leukemia. Blood.

[73] Masho S.W., Wang C.L., Nixon D.E. (2007). Review of tenofovir-emtricitabine. Ther. Clin. Risk Manag..

[74] Qi H., Wang Z., Song X., Wang M., Ding H., Ye H., Meng Z., He S., He Y., Li L. (2026). Efavirenz 400 mg vs. 600 mg Combined with Lamivudine and Tenofovir in treatment-naïve HIV-infected patients in China: A randomized, multi-centered, controlled trial. Int. J. Infect. Dis..

[75] Zakharenko A.L., Luzina O.A., Chepanova A.A., Dyrkheeva N.S., Salakhutdinov N.F., Lavrik O.I. (2023). Natural products and their derivatives as inhibitors of the DNA repair enzyme tyrosyl-DNA phosphodiesterase 1. Int. J. Mol. Sci..

[76] Pommier Y., Huang S.N., Gao R., Das B.B., Murai J., Marchand C. (2014). Tyrosyl-DNA-Phosphodiesterases (TDP1 and TDP2). DNA Repair.

[77] Yang S.W., Burgin A.B., Huizenga B.N., Robertson C.A., Yao K.C., Nash H.A. (1996). A Eukaryotic Enzyme That Can Disjoin Dead-End Covalent Complexes between DNA and Type I Topoisomerases. Proc. Natl. Acad. Sci. USA.

[78] Alagoz M., Gilbert D.C., El-Khamisy S., Chalmers A.J. (2012). DNA Repair and Resistance to Topoisomerase I Inhibitors: Mechanisms, Biomarkers and Therapeutic Targets. Curr. Med. Chem..

[79] Laev S.S., Salakhutdinov N.F., Lavrik O.I. (2016). Tyrosyl-DNA phosphodiesterase inhibitors: Progress and potential. Bioorg. Med. Chem..

[80] Brettrager E.J., van Waardenburg R.C.A.M. (2019). Targeting tyrosyl-DNA phosphodiesterase I to enhance toxicity of phosphodiester linked DNA adducts. Cancer Drug Resist..

[81] Zakharenko A., Dyrkheeva N., Lavrik O. (2019). Dual DNA topoisomerase 1 and tyrosyl-DNA phosphodiesterase 1 inhibition for improved anticancer activity. Med. Res. Rev..

[82] Dyrkheeva N.S., Chernyshova I.A., Ivanov G.A., Porozov Y.B., Zenchenko A.A., Oslovsky V.E., Zakharenko A.L., Nasyrova D.I., Likhatskaya G.N., Mikhailov S.N. (2022). In Vitro and In Silico studies of human tyrosyl-DNA phosphodiesterase 1 (Tdp1) inhibition by stereoisomeric forms of lipophilic nucleosides: The role of carbohydrate stereochemistry in ligand-enzyme interactions. Molecules.

[83] Schinazi R.F., McMillan A., Cannon D., Mathis R., Lloyd R.M., Peck A., Sommadossi J.P., St Clair M., Wilson J., Furman P.A. (1992). Selective Inhibition of Human Immunodeficiency Viruses by Racemates and Enantiomers of Cis-5-Fluoro-1-[2-(Hydroxymethyl)-1,3-Oxathiolan-5-Yl]Cytosine. Antimicrob. Agents Chemother..

[84] Schinazi R.F., Chu C.K., Peck A., McMillan A., Mathis R., Cannon D., Jeong L.S., Beach J.W., Choi W.B., Yeola S. (1992). Activities of the Four Optical Isomers of 2’,3’-Dideoxy-3’-Thiacytidine (BCH-189) against Human Immunodeficiency Virus Type 1 in Human Lymphocytes. Antimicrob. Agents Chemother..

[85] Schinazi R.F., Lloyd R.M., Nguyen M.H., Cannon D.L., McMillan A., Ilksoy N., Chu C.K., Liotta D.C., Bazmi H.Z., Mellors J.W. (1993). Characterization of Human Immunodeficiency Viruses Resistant to Oxathiolane-Cytosine Nucleosides. Antimicrob. Agents Chemother..

[86] Gumina G., Chong Y., Chu C.K., Peters G.J. (2006). L-Nucleosides as Chemotherapeutic Agents. Deoxynucleoside Analogs in Cancer Therapy.

[87] Shelton J., Lu X., Hollenbaugh J.A., Cho J.H., Amblard F., Schinazi R.F. (2016). Metabolism, biochemical actions, and chemical synthesis of anticancer nucleosides, nucleotides, and base analogs. Chem. Rev..

[88] Dantsu Y., Zhang Y., Zhang W. (2022). Advances in therapeutic L-nucleosides and L-nucleic acids with unusual handedness. Genes.

[89] Gumina G., Song G.-Y., Chu C.K. (2001). L-Nucleosides as Chemotherapeutic Agents. FEMS Microbiol. Lett..

[90] Jarvis B., Faulds D. (1999). Lamivudine: A Review of Its Therapeutic Potential in Chronic Hepatitis B. Drugs.

[91] Wang P., Hong J.H., Cooperwood J.S., Chu C.K. (1998). Recent Advances in L-Nucleosides: Chemistry and Biology. Antivir. Res..

[92] Kim T.E., Park S.-Y., Hsu C.-H., Dutschman G.E., Cheng Y.-C. (2004). Synergistic Antitumor Activity of Troxacitabine and Camptothecin in Selected Human Cancer Cell Lines. Mol. Pharmacol..

[93] Luo M.-Z., Liu M.-C., Mozdziesz D.E., Lin T.-S., Dutschman G.E., Gullen E.A., Cheng Y.-C., Sartorelli A.C. (2000). Synthesis and biological evaluation of L-and D-configuration 1, 3-dioxolane 5-azacytosine and 6-azathymine nucleosides. Bioorg. Med. Chem. Lett..

[94] Lin J.-S., Kira T., Gullen E., Choi Y., Qu F., Chu C.K., Cheng Y.-C. (1999). Structure−Activity Relationships of L-Dioxolane Uracil Nucleosides as Anti-Epstein Barr Virus Agents. J. Med. Chem..

[95] Bryant M.L., Bridges E.G., Placidi L., Faraj A., Loi A.-G., Pierra C., Dukhan D., Gosselin G., Imbach J.-L., Hernandez B. (2001). Anti-HBV Specific β-L-2’-Deoxynucleosides. Nucleosides Nucleotides Nucleic Acids.

[96] Dutschman G.E., Bridges E.G., Liu S.-H., Gullen E., Guo X., Kukhanova M., Cheng Y.-C. (1998). Metabolism of 2′,3′-Dideoxy-2′,3′-Didehydro-β-l(−)-5-Fluorocytidine and Its Activity in Combination with Clinically Approved Anti-Human Immunodeficiency Virus β-d(+) Nucleoside Analogs In Vitro. Antimicrob. Agents Chemother..

[97] Lin T.-S., Luo M.-Z., Liu M.-C., Zhu Y.-L., Gullen E., Dutschman G.E., Cheng Y.-C. (1996). Design and Synthesis of 2′,3′-Dideoxy- 2′,3′-Didehydro-β-L-Cytidine (β-L-d4C) and 2′,3′-Dideoxy-2′,3′-Didehydro-β-L-5-Fluorocytidine (β-L-Fd4C), Two Exceptionally Potent Inhibitors of Human Hepatitis B Virus (HBV) and Potent Inhibitors of Human Immunodeficiency Virus (HIV) in Vitro. J. Med. Chem..

[98] Park A.-Y., Kim W.H., Kang J.-A., Lee H.J., Lee C.-K., Moon H.R. (2011). Synthesis of enantiomerically pure D-and L-bicyclo[3.1.0]hexenyl carbanucleosides and their antiviral evaluation. Bioorg. Med. Chem..

[99] Zhu W., Gumina G., Schinazi R.F., Chu C.K. (2003). Synthesis and anti-HIV activity of L-β-3’-C-cyano-2’,3’-unsaturated nucleosides and L-3’-C-cyano-3’-deoxyribonucleosides. Tetrahedron.

[100] Chu C.K., Ma T., Shanmuganathan K., Wang C., Xiang Y., Pai S.B., Yao G.Q., Sommadossi J.P., Cheng Y.C. (1995). Use of 2’-Fluoro-5-Methyl-Beta-L-Arabinofuranosyluracil as a Novel Antiviral Agent for Hepatitis B Virus and Epstein-Barr Virus. Antimicrob. Agents Chemother..

[101] Yao G.-Q., Liu S.-H., Chou E., Kukhanova M., Chu C.K., Cheng Y.-C. (1996). Inhibition of Epstein-Barr Virus Replication by a Novel l-Nucleoside, 2′-Fluoro-5-Methyl-β-l-Arabinofuranosyluracil. Biochem. Pharmacol..

[102] Chen S.-H., Lin S., King I., Spinka T., Dutschman G.E., Gullen E.A., Cheng Y.-C., Doyle T.W. (1998). Synthesis and Comparative Evaluation of Two Antiviral Agents: β-L-Fd4C and β-D-Fd4C. Bioorg. Med. Chem. Lett..

[103] Lee K., Choi Y., Gullen E., Schlueter-Wirtz S., Schinazi R.F., Cheng Y.-C., Chu C.K. (1999). Synthesis and Anti-HIV and Anti-HBV Activities of 2’-Fluoro-2’,3’-Unsaturated l -Nucleosides. J. Med. Chem..

[104] Pai S.B., Pai R.B., Xie M.-Y., Beker T., Shi J., Tharnish P.M., Chu C.K., Schinazi R.F. (2005). Characterization of Hepatitis B Virus Inhibition by Novel 2′-Fluoro-2′,3′-Unsaturated Beta-D- and L-Nucleosides. Antivir. Chem. Chemother..

[105] Lee K., Choi Y., Gumina G., Zhou W., Schinazi R.F., Chu C.K. (2002). Structure− activity relationships of 2’-fluoro-2’,3’-unsaturated D-nucleosides as anti-HIV-1 agents. J. Med. Chem..

[106] Shewach D.S., Liotta D.C., Schinazi R.F. (1993). Affinity of the antiviral enantiomers of oxathiolane cytosine nucleosides for human 2′-deoxycytidine kinase. Biochem. Pharmacol..

[107] Daga P.R., Duan J., Doerksen R.J. (2010). Computational model of hepatitis B virus DNA polymerase: Molecular dynamics and docking to understand resistant mutations. Protein Sci..

[108] Furman P.A., Wilson J.E., Reardon J.E., Painter G.R. (1995). The Effect of Absolute Configuration on the Anti-HIV and Anti-HBV Activity of Nucleoside Analogues. Antivir. Chem. Chemother..

[109] Abbruzzese J.L., Schmidt S., Raber M.N., Levy J.K., Castellanos A.M., Legha S.S., Krakoff I.H. (1989). Phase I Trial of 1-(2′-Deoxy-2′-Fluoro-1-Beta-D-Arabinofuranosyl)-5-Methyluracil (FMAU) Terminated by Severe Neurologic Toxicity. Investig. New Drugs.

[110] Kukhanova M., Lin Z.-Y., Yas’co M., Cheng Y.-C. (1998). Unique Inhibitory Effect of 1-(2′-Deoxy-2′-Fluoro-β-l-Arabinofuranosyl)-5-Methyluracil 5′-Triphosphate on Epstein-Barr Virus and Human DNA Polymerases. Biochem. Pharmacol..

[111] Forsman J.J., Leino R. (2011). L-Pentoses in Biological and Medicinal Applications. Chem. Rev..

[112] Tyzeka: Package Insert/Prescribing Information. https://www.drugs.com/.

[113] Clercq E.D., Holý A. (2005). Acyclic Nucleoside Phosphonates: A Key Class of Antiviral Drugs. Nat. Rev. Drug Discov..

[114] De Clercq E. (2003). Clinical Potential of the Acyclic Nucleoside Phosphonates Cidofovir, Adefovir, and Tenofovir in Treatment of DNA Virus and Retrovirus Infections. Clin. Microbiol. Rev..

[115] Zakharova V.M., Serpi M., Krylov I.S., Peterson L.W., Breitenbach J.M., Borysko K.Z., Drach J.C., Collins M., Hilfinger J.M., Kashemirov B.A. (2011). Tyrosine-Based 1-(*S*)-[3-Hydroxy-2-(Phosphonomethoxy)Propyl]Cytosine and -Adenine ((*S*)-HPMPC and (*S*)-HPMPA) Prodrugs: Synthesis, Stability, Antiviral Activity, and in Vivo Transport Studies. J. Med. Chem..

[116] Mulato A.S., Cherrington J.M. (1997). Anti-HIV Activity of Adefovir (PMEA) and PMPA in Combination with Antiretroviral Compounds: In Vitro Analyses. Antivir. Res..

[117] Heijtink R.A., Kruining J., De Wilde G.A., Balzarini J., De Clercq E., Schalm S.W. (1994). Inhibitory Effects of Acyclic Nucleoside Phosphonates on Human Hepatitis B Virus and Duck Hepatitis B Virus Infections in Tissue Culture. Antimicrob. Agents Chemother..

[118] Arimilli M., Kim C., Dougherty J., Mulato A., Oliyai R., Shaw J., Cundy K., Bischofberger N. (1997). Synthesis, in Vitro Biological Evaluation and Oral Bioavailability of 9-[2-(Phosphonomethoxy)Propyl]Adenine (PMPA) Prodrugs. Antivir. Chem. Chemother..

[119] Callebaut C., Stepan G., Tian Y., Miller M.D. (2015). In Vitro Virology Profile of Tenofovir Alafenamide, a Novel Oral Prodrug of Tenofovir with Improved Antiviral Activity Compared to That of Tenofovir Disoproxil Fumarate. Antimicrob. Agents Chemother..

[120] Holý A. (2006). Antiviral Acyclic Nucleoside Phosphonates Structure Activity Studies. Antivir. Res..

[121] Chapman T.M., McGavin J.K., Noble S. (2003). Tenofovir Disoproxil Fumarate. Drugs.

[122] Ray A.S., Fordyce M.W., Hitchcock M.J.M. (2016). Tenofovir Alafenamide: A Novel Prodrug of Tenofovir for the Treatment of Human Immunodeficiency Virus. Antivir. Res..

[123] De Clercq E. (2018). Role of tenofovir alafenamide (TAF) in the treatment and prophylaxis of HIV and HBV infections. Biochem. Pharmacol..

[124] Seley-Radtke K.L., Yates M.K. (2018). The Evolution of Nucleoside Analogue Antivirals: A Review for Chemists and Non-Chemists. Part 1: Early Structural Modifications to the Nucleoside Scaffold. Antivir. Res..

[125] Hao E.-J., Li G.-X., Liang Y.-R., Xie M.-S., Wang D.-C., Jiang X.-H., Cheng J.-Y., Shi Z.-X., Wang Y., Guo H.-M. (2021). Design, Synthesis, and Activity Evaluation of Novel Acyclic Nucleosides as Potential Anticancer Agents In Vitro and In Vivo. J. Med. Chem..

[126] Drenichev M.S., Oslovsky V.E., Sun L., Tijsma A., Kurochkin N.N., Tararov V.I., Chizhov A.O., Neyts J., Pannecouque C., Leyssen P. (2016). Modification of the Length and Structure of the Linker of N6-Benzyladenosine Modulates Its Selective Antiviral Activity against Enterovirus 71. Eur. J. Med. Chem..

[127] Tararov V.I., Tijsma A., Kolyachkina S.V., Oslovsky V.E., Neyts J., Drenichev M.S., Leyssen P., Mikhailov S.N. (2015). Chemical Modification of the Plant Isoprenoid Cytokinin N6-Isopentenyladenosine Yields a Selective Inhibitor of Human Enterovirus 71 Replication. Eur. J. Med. Chem..

[128] Arita M., Wakita T., Shimizu H. (2008). Characterization of pharmacologically active compounds that inhibit poliovirus and enterovirus 71 infectivity. J. Gen. Virol..

[129] Ottria R., Casati S., Baldoli E., Maier J.A.M., Ciuffreda P. (2010). N6-Alkyladenosines: Synthesis and Evaluation of in Vitro Anticancer Activity. Bioorg. Med. Chem..

[130] Voller J., Béres T., Zatloukal M., Džubák P., Hajdúch M., Doležal K., Schmülling T., Miroslav S. (2019). Anti-cancer activities of cytokinin ribosides. Phytochem. Rev..

[131] Voller J., Zatloukal M., Lenobel R., Doležal K., Béreš T., Kryštof V., Spíchal L., Niemann P., Džubák P., Hajdúch M. (2010). Anticancer activity of natural cytokinins: A structure–activity relationship study. Phytochemistry.

[132] Tosh D.K., Toti K.S., Hurst B.L., Julander J.G., Jacobson K.A. (2020). Structure Activity Relationship of Novel Antiviral Nucleosides against Enterovirus A71. Bioorg. Med. Chem. Lett..

[133] Saleh A., D’Angelo J.G., Morton M.D., Quinn J., Redden K., Mielguz R.W., Pavlik C., Smith M.B. (2011). Synthesis of 3-guaninyl-and 3-adeninyl-5-hydroxymethyl-2-pyrrolidinone nucleosides. J. Org. Chem..

[134] Bras A.P., Sitar D.S., Aoki F.Y. (2001). Comparative Bioavailability of Acyclovir from Oral Valacyclovir and Acyclovir in Patients Treated for Recurrent Genital Herpes Simplex Virus Infection. Can. J. Clin. Pharmacol..

[135] Chamberlain C.E., Penzak S.R., Alfaro R.M., Wesley R., Daniels C.E., Hale D., Kirk A.D., Mannon R.B. (2008). Pharmacokinetics of Low and Maintenance Dose Valganciclovir in Kidney Transplant Recipients. Am. J. Transplant..

[136] Piketty C., Bardin C., Gilquin J., Gairard A., Kazatchkine M.D., Chast F. (2000). Monitoring Plasma Levels of Ganciclovir in AIDS Patients Receiving Oral Ganciclovir as Maintenance Therapy for CMV Retinitis. Clin. Microbiol. Infect..

[137] Berzina M.Y., Eletskaya B.Z., Kayushin A.L., Dorofeeva E.V., Lutonina O.I., Fateev I.V., Paramonov A.S., Kostromina M.A., Zayats E.A., Abramchik Y.A. (2022). Synthesis of 2-Chloropurine Ribosides with Chiral Amino Acid Amides at C6 and Their Evaluation as A1 Adenosine Receptor Agonists. Bioorg. Chem..

[138] Koplūnaitė M., Butkutė K., Stankevičiūtė J., Meškys R. (2024). Exploring the Mutated Kinases for Chemoenzymatic Synthesis of N4-Modified Cytidine Monophosphates. Molecules.

[139] Wang Q., Yang M., Zhang Y., Zhong L., Zheng X. (2019). Novel Combination Oncolytic Adenoviral Gene Therapy Armed with Dm-dNK and CD40L for Breast Cancer. Curr. Gene Ther..

[140] Fillat C., Carrio M., Cascante A., Sangro B. (2003). Suicide Gene Therapy Mediated by the Herpes Simplex Virus Thymidine Kinase Gene/Ganciclovir System: Fifteen Years of Application. Curr. Gene Ther..

[141] Ma S., Zhao L., Zhu Z., Liu Q., Xu H., Johansson M., Karlsson A., Zheng X. (2011). The Multisubstrate Deoxyribonucleoside Kinase of *Drosophila Melanogaster* as a Therapeutic Suicide Gene of Breast Cancer Cells. J. Gene Med..

[142] Vorbruggen H., Ruh-Pohlenz C. (2001). Handbook of Nucleoside Synthesis.

[143] Kore A.R., Yang B., Srinivasan B. (2014). Recent Developments in the Synthesis of Substituted Purine Nucleosides and Nucleotides. Curr. Org. Chem..

[144] Mikhailopulo I.A., Miroshnikov A.I. (2011). Biologically important nucleosides: Modern trends in biotechnology and application. Mendeleev Commun..

[145] Alexeev C.S., Kulikova I.V., Gavryushov S., Tararov V.I., Mikhailov S.N. (2018). Quantitative prediction of yield in transglycosylation reaction catalyzed by nucleoside phosphorylases. Adv. Synth. Catal..

[146] Drenichev M.S., Alexeev C.S., Kurochkin N.N., Mikhailov S.N. (2018). Use of nucleoside phosphorylases for the preparation of purine and pyrimidine 2′-deoxynucleosides. Adv. Synth. Catal..

[147] Eletskaya B.Z., Berzina M.Y., Fateev I.V., Kayushin A.L., Dorofeeva E.V., Lutonina O.I., Zorina E.A., Antonov K.V., Paramonov A.S., Muzyka I.S. (2023). Enzymatic synthesis of 2-chloropurine arabinonucleosides with chiral amino acid amides at the C6 position and an evaluation of antiproliferative activity in vitro. Int. J. Mol. Sci..

[148] Rabuffetti M., Bavaro T., Semproli R., Cattaneo G., Massone M., Morelli C.F., Speranza G., Ubiali D. (2019). Synthesis of ribavirin, tecadenoson, and cladribine by enzymatic transglycosylation. Catalysts.

[149] Cruz G., Acosta J., Del Arco J., Clemente-Suarez V.J., Deroncele V., Fernández-Lucas J. (2022). Enzyme-mediated synthesis of Molnupiravir: Paving the way for the application of biocatalysis in pharmaceutical industry. ChemCatChem.

[150] Veres Z., Szabolcs A., Szinai I., Dénes G., Kajtár-Peredy M., Ötvös L. (1985). 5-Substituted-2,2′-Anhydrouridines, Potent Inhibitors of Uridine Phosphorylase. Biochem. Pharmacol..

[151] Gissot A., Massip S., Barthélémy P. (2020). Intramolecular Michael Additions in Uridine Derivatives: Isolation of the Labile 5′O-C6 Cyclonucleoside. ACS Omega.

[152] Cheng Y., Zheng W., Dong X., Sun T., Xu M., Xiang L., Li J., Wang H., Jian X., Yu J. (2025). Design and Development of a Novel Oral 4’-Fluorouridine Double Prodrug VV261 against SFTSV. J. Med. Chem..

[153] Zhang Y., Tang S., Chen L., Song S., Cao J., Tian G., Xiao G., Shen J., Zhang L. (2026). Investigation of a clinical trial drug VV261 as a potent antiviral candidate against Chikungunya virus. Signal Transduct. Target. Ther..

[154] Meier C., Balzarini J., Meerbach A., Peters G.J. (2006). The *cyclo*Sal-nucleotide Delivery System. Development of Chemical Trojan Horses as Antiviral Agents. Deoxynucleoside Analogs in Cancer Therapy.

[155] WHO Solidarity Trial Consortium (2021). Repurposed antiviral drugs for COVID-19—Interim WHO solidarity trial results. N. Engl. J. Med..

[156] Zhang Q. (2026). Acyclic Nucleoside Phosphonates: The Art of the Prodrug. Pharm. Front..

[157] Liang L., Meng Y., Chang X., Li E., Huang Y., Yan L., Lou Z., Peng Y., Zhu B., Yu W. (2025). Discovery of a 2’-α-Fluoro-2’-β-C-(fluoromethyl) Purine Nucleotide Prodrug as a Potential Oral Anti-SARS-CoV-2 Agent. J. Med. Chem..

[158] Elfiky A.A., Azzam E.B., Shafaa M.W. (2022). The anti-HCV, Sofosbuvir, versus the anti-EBOV Remdesivir against SARS-CoV-2 RNA dependent RNA polymerase in silico. Mol. Div..

[159] Zhang H.-W., Detorio M., Herman B.D., Solomon S., Bassit L., Nettles J.H., Obikhod A., Tao S.-J., Mellors J.W., Sluis-Cremer N. (2011). Synthesis, antiviral activity, cytotoxicity and cellular pharmacology of L-3’-azido-2’,3’-dideoxypurine nucleosides. Eur. J. Med. Chem..

[160] Wengel J., Lau J., Pederson E.B., Nielson C.M. (1991). Synthesis of L-3’-azido-3’-deoxythymidine and its stereoisomers. J. Org. Chem..

[161] Butora G., Qi N., Fu W., Nguyen T., Huang H., Davies I.W. (2014). Cyclic-Disulfide-Based Prodrugs for Cytosol-Specific Drug Delivery. Angew. Chem. Int. Ed..

